# Best Disease: Global Mutations Review, Genotype–Phenotype Correlation, and Prevalence Analysis in the Israeli Population

**DOI:** 10.1167/iovs.65.2.39

**Published:** 2024-02-27

**Authors:** Avigail Beryozkin, Ifat Sher, Miriam Ehrenberg, Dinah Zur, Hadas Newman, Libe Gradstein, Francis Simaan, Ygal Rotenstreich, Nitza Goldenberg-Cohen, Irit Bahar, Anat Blumenfeld, Antonio Rivera, Boris Rosin, Iris Deitch-Harel, Ido Perlman, Hadas Mechoulam, Itay Chowers, Rina Leibu, Tamar Ben-Yosef, Eran Pras, Eyal Banin, Dror Sharon, Samer Khateb

**Affiliations:** 1 Department of Ophthalmology, Hadassah Medical Center, Faculty of Medicine, The Hebrew University of Jerusalem, Jerusalem, Israel; 2 Department of Ophthalmology, University of Pittsburgh, Pittsburgh, Pennsylvania, United States; 3 Goldschleger Eye Institute, Sheba Medical Center, Faculty of Medicine, Tel Aviv University, Tel Aviv, Israel; 4 Ophthalmology Unit, Schneider Children's Medical Center, Faculty of Medicine, Tel Aviv University, Tel Aviv, Israel; 5 Ophthalmology Division, Tel Aviv Medical Center, Faculty of Medicine, Tel Aviv University, Tel Aviv, Israel; 6 Department of Ophthalmology, Soroka Medical Center and Clalit Health Services, Faculty of Health Sciences, Ben-Gurion University, Be'er Sheva, Israel; 7 Department of Ophthalmology, Assaf Harofeh Medical Center, Zerifin, Israel; 8 Department of Ophthalmology, Bnai Zion Medical Center, Haifa, Israel; 9 Rappaport Faculty of Medicine, Technion-Israel Institute of Technology, Haifa, Israel; 10 Ophthalmology Department and Laboratory of Eye Research, Felsenstein Medical Research Center, Rabin Medical Center, Petach Tikva, Israel; 11 Department of Ophthalmology, Rambam Health Care Center, Haifa, Israel; 12 Faculty of Medicine, Tel Aviv University, Tel Aviv, Israel

**Keywords:** BEST1, macular degeneration, prevalence, genotype-phenotype correlation, mutations review

## Abstract

**Purpose:**

To review all reported disease-causing mutations in *BEST1*, perform genotype–phenotype correlation, and estimate disease prevalence in the Israeli population.

**Methods:**

Medical records of patients diagnosed with Best disease and allied diseases from nine Israeli medical centers over the past 20 years were collected, as were clinical data including ocular findings, electrophysiology results, and retina imaging. Mutation detection involved mainly whole exome sequencing and candidate gene analysis. Demographic data were obtained from the Israeli Bureau of Statistics (January 2023). A bibliometric study was also conducted to gather mutation data from online sources.

**Results:**

A total of 134 patients were clinically diagnosed with Best disease and related conditions. The estimated prevalence of Best disease was calculated to be 1 in 127,000, with higher rates among Arab Muslims (1 in 76,000) than Jews (1 in 145,000). Genetic causes were identified in 76 individuals (57%), primarily showing autosomal-dominant inheritance due to *BEST1* mutations (58 patients). Critical conserved domains were identified consisting of a high percentage of dominant missense mutations, primarily in transmembrane domains and the intracellular region (Ca^2+^ binding domain) of the BEST1 protein.

**Conclusions:**

This study represents the largest cohort of patients with Best disease reported in Israel and globally. The prevalence in Israel is akin to that in Denmark but is lower than that in the United States. Critical conserved domains within the BEST1 protein are pivotal for normal functioning, and even minor missense alterations in these areas lead to a dominant disease manifestation. Genetic testing is indispensable as the gold standard for Best disease diagnosis due to the variable clinical presentation of the disease.

Best vitelliform macular dystrophy (BVMD, MIM#153700), known as Best disease, is a progressive macular degeneration first described in 1905 and usually caused by heterozygous mutations in *BEST1* (VMD2).^1^^–^^3^ Clinically, it is characterized by several retinal changes, including normal fundus appearance in early stages and typical “egg-yolk” macular lesion (see Supplementary Fig. S1A), reflecting abnormal accumulation of lipofuscin within and beneath the retinal pigment epithelium (RPE) in later stages.^4^^,^^5^ Disruption of this lesion is accompanied by a visual acuity drop, culminating in macular atrophy in late adolescence or adulthood. RPE responses, measured by electrooculography (EOG), are markedly affected, manifesting in a severely reduced Arden ratio accompanied by normal or mildly reduced full-field electroretinographic (ffERG) responses. BVMD can also be inherited in an autosomal-recessive (AR)^6^^–^^10^ or autosomal-dominant (AD) manner with reduced penetrance.^11^^,^^12^

*BEST1* encodes a 585-amino acid (AA) protein named bestrophin-1 (BEST1). Several studies strongly indicate that BEST1 is a Cl^−^ channel activated by Ca^2^^+^ or a regulator of ion transport, or both,^13^ and probably forms dimers or tetramers/pentamers.^14^^–^^16^ Heterozygous mutations in *BEST1* were reported to cause adult-onset vitelliform macular dystrophy (AVMD)^17^ and AD vitreoretinochoroidopathy (ADVIRC).^18^ The clinical features of AVMD partially overlap those of BVMD; the symptoms appear at an advanced age (fourth or fifth decade), and the EOG is within the normal to subnormal range. The clinical features of ADVIRC are completely different, including retinal and vitreal involvement, as well as ocular developmental abnormalities such as nanophthalmos, microcornea, closed-angle glaucoma, and congenital cataract^18^ (see Supplementary Fig. S1B). Biallelic mutations of *BEST1* lead to a distinct retinopathy termed AR bestrophinopathy, characterized by reduced ffERG responses, severe reduction of the EOG light rise, and smaller “egg-yolk” lesions extending outside of the macula^19^^–^^21^ (see Supplementary Fig. S1C). Recently, *BEST1* missense mutations have also been reported to cause retinitis pigmentosa (RP) inherited in an AD or AR fashion^22^ (see Supplementary Fig. S1D).

The current study focused on analyzing the spectrum of reported *BEST1* mutations and resulting phenotypes, as well as summarizing the genetic and clinical data of diagnosed Israeli patients with Best disease in order to determine the prevalence of the disease.

## Methods

### Subjects

This study adhered to the tenets of the Declaration of Helsinki. Prior to a blood sample being drawn for DNA analysis, informed consent was obtained from all patients and family members who participated in this study. Ethical approval was obtained from an ethics committee of each institute.

### Molecular Genetic Analysis

Genomic DNA was extracted from peripheral blood using the FlexiGene DNA Kit (QIAGEN, Hilden, Germany). Primers for exons 1 to 3 and 5 to 11 of *BEST1* were previously reported.^5^ Primers for exon 4 (forward: 5′AGAAAGCTGGAGGAGCCGA3′; reverse: 5′-TCCACCCATCTTCCATTCCTGC3′) were designed using Primer3 software (http://frodo.wi.mit.edu/cgi-bin/primer3/primer3_www.cgi) to screen the *BEST1* exons and exon–intron boundaries (National Center for Biotechnology Information [NCBI] reference sequence NM_004183.4). PCR testing was performed, and the mutations were analyzed by direct sequencing of the PCR products. Inclusion criteria for novel variants were mutation type, extremely low frequency in the Genome Aggregation Database (gnomAD; less than 0.5% for recessive variants and 0.05% for dominant variants), cosegregation in the family (when possible), and high scores of pathogenicity by in silico prediction tools. To estimate the frequency of c.967G>A (p.Asp323Asn) in the Jewish population, a set of 114 ethnicity-matched normal controls was used. The possible pathogenicity of novel missense changes was evaluated using PolyPhen-2 (http://genetics.bwh.harvard.edu/pph2/), MutationTaster (http://www.mutationtaster.org/), and SIFT (http://sift.jcvi.org/). American College of Medical Genetics and Genomics guidelines were used to evaluate the possible pathogenicity of novel variants.

### Bioinformatics Analysis

Protein structures, protein domains, and functional sites were identified and sketched using various online platforms: ProSite (https://prosite.expasy.org/), Pfam (http://pfam.xfam.org/), HaMap (https://hamap.expasy.org/), UniProt (https://www.uniprot.org/help/motif), and Motif Scan (https://myhits.sib.swiss/cgi-bin/motif_scan). Variants altering splice sites were identified with the Splicing Prediction Pipeline (SPiP; https://sourceforge.net/projects/splicing-prediction-pipeline/). Sliding window analysis was performed as previously described,^23^ with a 30-AA interval between the human *BEST1* sequence and each representative ortholog.

The AA sequences of *BEST1* orthologs were extracted from the NCBI HomoloGene database (http://www.ncbi.nlm.nih.gov/homologene/). The following sequences were used for the analysis: human (NP_004174.1), chimpanzee (XP_001151529.1), macaque (XP_014969466.2), horse (XP_014585095.1), cow (XP_015316720.1), dog (XP_038279399.1), rabbit (XP_017206051.1), rat (XP_038964543.1), mouse (NP_036043.2), chicken (XP_421055.3), frog (XP_040184347.1), zebrafish (ENSDART00000193249.1), and fruit fly (NP_001247018.1). AA sequences were aligned with the European Bioinformatics Institute ClustalW2 multiple sequence alignment tool (http://www.ebi.ac.uk/Tools/clustalw2/index.html).

### Pathogenicity Analysis

To better assess the pathogenicity of the novel missense variant c.967G>A (p.Asp323Asn), we used a set of tools to study the evolutionary conservation of the affected AA and examined the minor allele frequency in the gnomAD database, including the next-generation sequencing data of more than 140,000 individuals (see Supplementary Table S1). The results indicate that the novel missense mutation is likely pathogenic.

### Establishment of *BEST1* Pathogenic Variants Database

Pathogenic variants were extracted from the most updated databases: Leiden Open Variation Database 3 (LOVD3; https://databases.lovd.nl/shared/genes/BEST1), Human Gene Mutation Database (HGMD; https://www.hgmd.cf.ac.uk/ac/gene.php?gene=BEST1), and PubMed (https://pubmed.ncbi.nlm.nih.gov/) in January 2023. Because several previous studies have shown that not all variants classified as pathogenic in the HGMD necessarily cause disease,^24^^,^^25^ we used several filtering steps. The NCBI reference sequence NM_004183.4 was used in the current study. When mutations were reported previously using other reference sequences, they were adapted to the current reference sequences either by using the online program FRANKLIN (https://franklin.genoox.com/) or manually. Excluded from analysis were variants with high allele frequency (>0.005) according to one of the following: gnomAD (https://gnomad.broadinstitute.org/), 4.7KJPN (https://jmorp.megabank.tohoku.ac.jp/), GenomeAsia (https://www.genomeasia100k.org/), or the Greater Middle East (GME) Variome Project (http://igm.ucsd.edu/gme/). Variants that were identified in one patient without segregation analysis and/or without well-established clinical characteristics were excluded, as well. Silent variants were not included in this research. For additional methods, see Supplementary Methods.

## Results

### Genetics of BVMD in the Israeli Population

To identify the majority of patients diagnosed with Best disease in Israel, we recruited 134 affected individuals diagnosed clinically and electrophysiologically from nine different centers. The number of patients recruited by each center is shown in Supplementary Figure S2. Aiming to identify the genetic cause of Best disease in the 134 patients recruited to this study, we screened all 11 *BEST1* exons and exon–intron boundaries in 56 index cases and identified likely pathogenic mutations in 43 (∼77%) of the families (see Supplementary Fig. S3). In nine families, no mutation was identified; in four additional families, pathogenic mutations were identified using whole exome sequencing in *PRPH2* and *IMPG2*, which were previously reported to cause retinal degenerations similar to the BVMD phenotype (see Supplementary Table S2).^26^^,^^27^ Among the genetically solved patients, 76 patients were identified as harboring disease-causing mutations in *BEST1*, and AD mutations were identified in 58 of them, confirming the more common inheritance pattern of BVMD in the Israeli population. Four mutations were novel; all of them cause AR bestrophinopathy: missense (c.967G>A), nonsense (c.970G>T), splicing (c.1740-1G>C), and small deletion (c.1622del). Evaluation of pathogenicity was done as described in the Methods and Supplementary Table S1. Among the 20 mutations identified in our cohort, six were recurrent (see Supplementary Table S3) in families who shared the same origin and are therefore likely to result from a founder effect.

### Clinical Characterization of Families With *BEST1* Mutations

We identified a wide spectrum of phenotypes among the 76 patients with *BEST1* mutations, with inter- and intrafamilial variability, as detailed in Supplementary Table S2, Supplementary Table S4, and Supplementary Results.

### BVMD Prevalence: Genetic and Phenotypic

Aiming to estimate the prevalence of BVMD in Israel, we created a database based on fundus findings and EOG results (see Supplementary Table S4). A total of 134 patients (72 families) were recruited, and 76 patients (from 43 families) harbored *BEST1* mutations. Therefore, the estimated upper limit for BVMD was 1 in 72,000 individuals. The upper limit was calculated by dividing the population size (9,656,000 in January 2023, based on the Israeli Bureau of Statistics) by 134 cases with BVMD. Similarly, the prevalence on *BEST1* cases was set to 1 in 127,000 individuals. The lower limit was calculated by dividing population size by 76. The lower limit includes only those who were clinically diagnosed with BVMD and genetically diagnosed with *BEST1* mutations (76 patients). The prevalence among the Jewish population is estimated to range from 1 in 83,600 to 1 in 145,000; among Arab Muslims, from 1 in 46,250 to 1 in 76,000.

**Figure 1.**
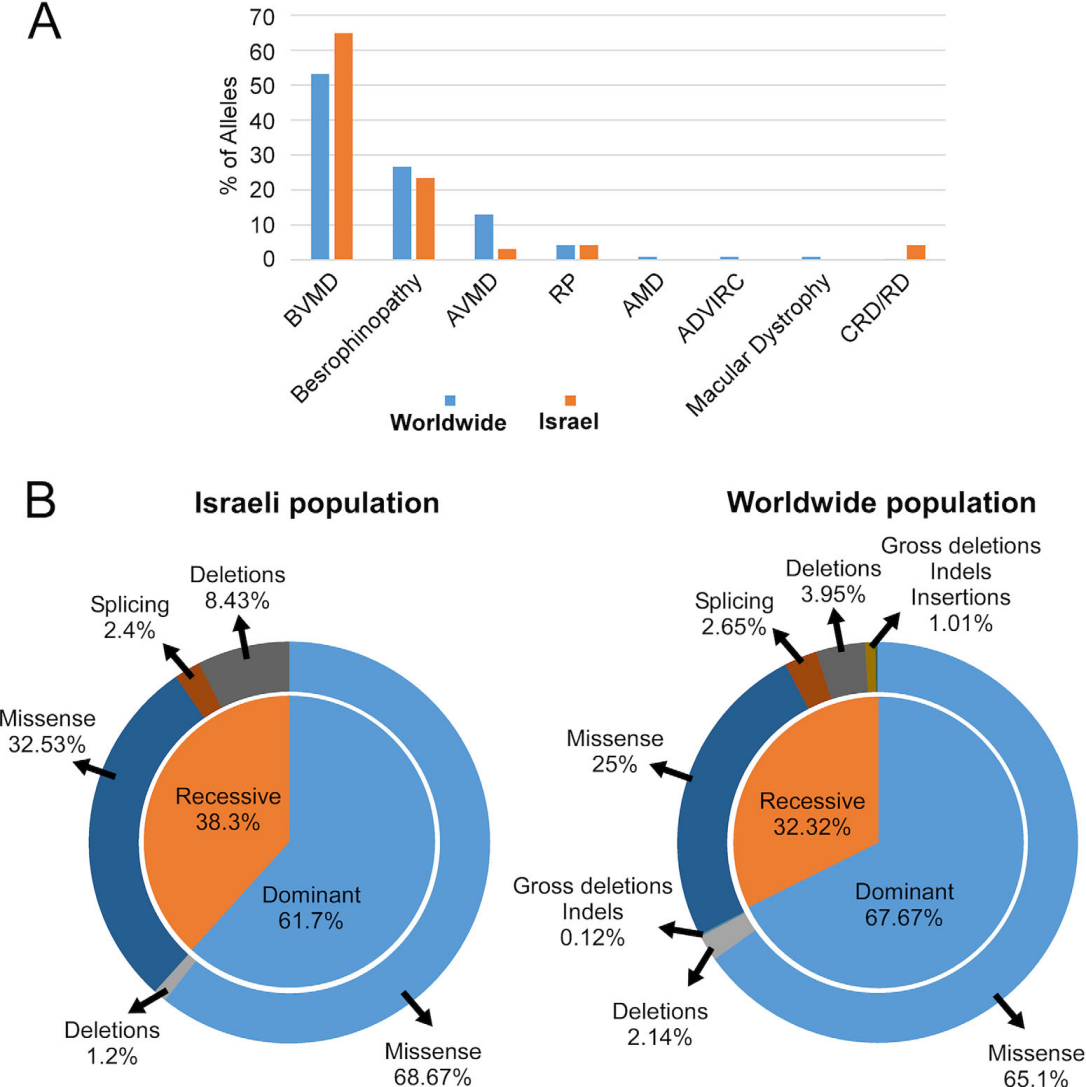
Distribution of patients and mutated *BEST1* alleles in the Israeli population compared to reported mutations in large databases. (**A**) The distribution of *BEST1* mutated alleles in the studied set of Israeli patients compared to the alleles reported previously. The data were calculated and are presented in percent (%). Each *bar* represents the percentage of alleles associated with a certain clinical diagnosis among the Israeli cohort of *BEST1* (*orange*) and worldwide (*blue*). (**B**) The distribution of mutated *BEST1* alleles identified in patients from the Israeli cohort (*left pie chart*) and alleles reported worldwide (*right pie chart*) based on recessive/dominant alleles (*inner circle*) and the type of the mutation (*outer circle*).

As expected, most of the patients were diagnosed with BVMD, as it is the most frequent phenotype in the worldwide population as well (Fig. 1A).^28^ The prevalence of alleles responsible for BVMD in the Israeli population was notably higher at 65% compared to the 53% reported in a comprehensive mutations analysis. This discrepancy may arise from the fact that patients who are clinically diagnosed with conditions that can resemble the initial or final stages of Best disease, such as age-related macular degeneration, RP, cone–rod dystrophies, and AVMD, are typically not suspected to have Best disease and consequently do not undergo genetic testing for mutations in *BEST1* (Fig. 1A; Supplementary Tables S2, S5), which could result in an underdiagnosis of *BEST1* mutations. We subsequently compared the percentage of dominant and recessive alleles among our population (Fig. 1B, left panel) to the percentage of all reported alleles in the literature (Fig. 1B, right panel). The percentage of recessive alleles is slightly higher in the Israeli population (38.3%) compared to the worldwide incidence (32.32%). This finding might be influenced by the high rate of consanguinity and intracommunity marriages in the Israeli population. In addition, most of the dominant and recessive alleles were missense in both groups, whereas gross deletions, indels, and insertions were not identified among our cohort.

### BEST1: Structure and Mutation Analysis

**Figure 2.**
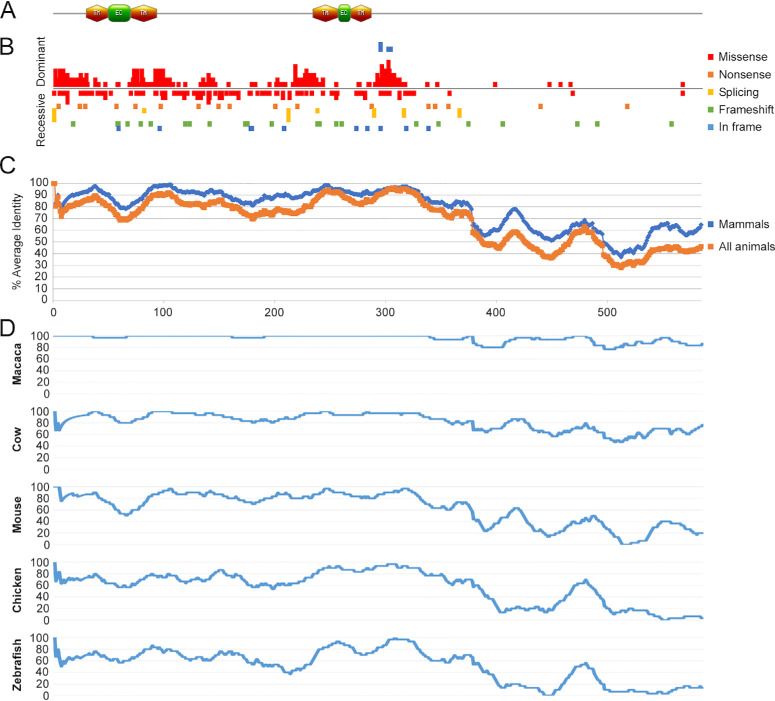
Protein domains and evolutionary analysis of BEST1. (**A**) A schematic representation of the BEST1 protein. *Orange hexagons* represent the transmembrane domain (TM), *green rectangles* represent extracellular regions (ECs), and *gray* *lines* represent the intracellular regions. The *x*-axis is AA number. (**B**) Presentation of the distribution of all of the reported mutations. Different mutation types are presented in different colors (missense in *red*, nonsense in *orange*, splicing in *yellow*, frameshift in *green*, and in-frame deletions, insertions, or indel mutations in *blue*). All mutations were also sorted according to the inheritance manner (dominant above the line and recessive under the line). The *x*-axis is AA number. (**C**) An AA sliding window comparing the human protein sequence to orthologs of all animals (*orange*), including chimp, macaque, dog, horse, cow, rabbit, rat, mouse, chicken, frog, zebrafish, and fruit fly, and orthologs of mammals only (*blue*). The *x*-axis is AA number; the *y*-axis is the percentage of AA identity in a 30-AA window. The average percentages of AA identity for each studied sequence were as follows: chimp, 100%; macaque, 97%; horse, 82%; cow, 80%; dog, 79%; rabbit, 71%; rat, 60%; mouse, 64%; chicken, 56%; frog, 51%; zebrafish, 51%; and fruit fly, 35%. (**D**) An AA sliding window comparing the human protein sequence to each of the representative orthologs (macaque, cow, mouse, chicken, and zebrafish).

BEST1 is a calcium-activated chloride channel, which consists of four to six transmembrane domains,^17^^,^^29^^–^^34^ with at least two short extracellular regions and several long cytoplasmic regions. We used five different online platforms to predict protein domains, structure, and topology (Fig. 2A). A comprehensive analysis of reported mutations was performed including data collection of pathogenic variants reported previously, sorted according to mutation type and inheritance pattern and presented according to the location along the protein sequence (Fig. 2B).

Most of the mutations reported so far are missense, predicted to affect the structure and/or the function of the protein (351 different mutations representing 2141 alleles). Most of the missense mutations are inherited in a dominant manner (251 mutations, compared to 100 recessive). The remaining mutations are nonsense (17 mutations, 74 alleles), splicing (16 mutations, 63 alleles), frameshift (27 mutations, 87 alleles), and in-frame deletions or insertions (16 mutations, 85 alleles). Nonsense, splicing, and frameshift pathogenic variants cause a recessive disease. An interesting exception are in-frame mutations, which usually cause a recessive disease, but, if the deletion alters AAs 295 to 304, then the inheritance tends to be dominant (Fig. 2B).

The analysis shows that nonsense, splicing, frameshift, in-frame deletions/insertions, and indel mutations are scattered along the protein, whereas missense mutations are concentrated mainly in the N-terminal region of the protein. Several areas along the protein are less tolerant to changes, mainly N-terminal regions that are predicted to be transmembranal (AAs 31–50, 71–94, 235–257, and 269–287) or intracellular (AAs 1–30 and 95–234 and the first ∼35 AAs of the last intracellular region 288–585). Surprisingly, areas between the transmembrane domains that are predicted to be extracellular (AAs 51–70 and 258–268) are much more tolerant to changes, and only five missense mutations were found there (Fig. 2B).

A sliding window analysis of *BEST1* orthologs was performed aiming to present the conservation pattern of *BEST1* with evolution (Figs. 2C, 2D). The analysis highlights a few highly conserved regions that correspond to protein domains, including the two N-terminal intracellular regions, the four transmembrane domains, and the following short sequence of the intracellular region. These regions correspond to rich missense mutations areas, indicating that any change, including missense, is unlikely to be tolerated. The two non-conserved extracellular domains, in comparison, contain a much lower number of mutations, thus indicating probably more tolerable changes.

**Figure 3.**
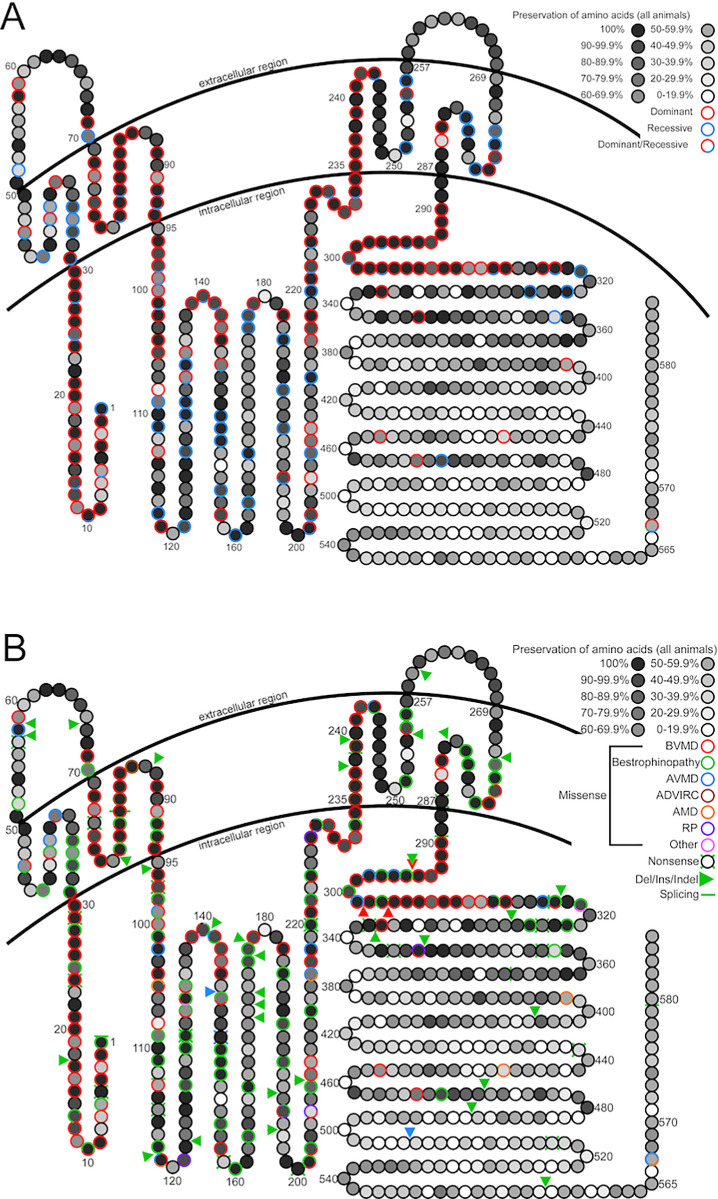
BEST1 protein model (adapted from Refs. 32, 51, and 52). (**A**) Preservation of AAs and inheritance pattern of missense mutations. The preservation of each AA among all examined animals is presented. *Darker circles* represent more preserved AAs; the level of preservation is interpreted in the *upper right corner* of the figure. Dominant inheritance is indicated by a *red ring*, recessive inheritance is indicated by a blue ring, and missense pathogenic variants in a specific AA that can be inherited in a dominant or recessive manner are indicated by *red* and *blue rings*. (**B**) Preservation of AAs and disease type associated with different mutations in *BEST1*. Mutations associated with specific clinical diagnosis are shown in different colors: *red* for BVMD, *green* for bestrophinopathy, *blue* for AVMD, *brown* for ADVIRC, *orange* for age-related macular degeneration, *purple* for RP, and *pink* for other diseases such as macular degeneration, retinopathies, and cone–rod dystrophies. *Colorful rings* represent missense mutations. The *ring* with an X underneath it represents nonsense mutations; the *triangle* represents insertions, deletions, or indels; and the *short line* represents splicing mutations.

Subsequently, we examined whether the conservation level of a specific AA affects the inheritance pattern or disease type. Compatible with our finding that nonsense, splicing, frameshift, and most of the in-frame mutations cause a recessive disease (Fig. 2B), we examined whether the location along the protein or the conservation of a specific AA correlates with the inheritance pattern of missense mutations. Hence, we plotted the conservation level of each AA and the inheritance pattern of missense mutations only (Fig. 3A) and the disease type of all mutations (Fig. 3B). We identified several regions that were extremely conserved (Fig. 3A, Supplementary Table S6): the first intracellular region (IC1); the second, third, and the fourth transmembranal regions (TM2, TM3, and TM4); and part of the last intracellular region (IC3) that is adjacent to the last transmembranal region. The calculated mutation prevalence (number of missense mutations divided by the number of AAs in each region) demonstrated three regions with high missense mutation prevalence, partially correlating with the conservation levels: IC1, TM2, and IC3. Among them, more than 90% of the missense mutations cause a dominant disease. Surprisingly, two less conserved regions (TM1 and TM4) had high percentages of missense mutations causing a recessive disease (Fig. 3A, Supplementary Table S6).

Similarly, by analyzing all mutation types, we identified three regions with high mutation prevalence (Fig. 3B): IC1, TM2, and IC3. In these regions, more than 80% of the mutations cause BVMD. The IC2 domain consists of the largest number of mutations causing recessive disease (*n* = 71). In addition, three regions had a high prevalence of recessive mutations (>75%): TM1, EC1, and TM4 (Fig. 3B, Supplementary Table S7).

## Discussion

The first prevalence estimation of all macular dystrophies was carried out in northern Sweden and reported a prevalence of 2 in 10,000. This study was performed before the identification of *BEST1*^2^ based on 95 individuals, 75 of whom belonged to a single family, a fact that may be affected by the presence of founder mutations.^35^ Later, the prevalence of BVMD was estimated in Denmark as ranging from 0.8 to 1.5 in 100,000, based on data from 45 individuals identified clinically and genetically with *BEST1* mutations.^36^ Additional research conducted in Olmsted County, Minnesota, estimated the prevalence of vitelliform dystrophies as 1 in 5500, and the prevalence of BVMD ranged from 1 in 16,500 to 1 in 21,000. This research was based on 79 clinically diagnosed patients, only two of whom were also genetically diagnosed.^37^ Because of the low genetic diagnosis rate, this might represent an overestimation. In the current study, the calculated prevalence (0.78–1.38 in 100,000) is likely to serve as an accurate prevalence assessment in Israel, similar to the reported disease prevalence in Denmark.^38^^,^^39^ Because our consortium includes all electroretinographic centers and inherited retinal disorder research genetic laboratories in Israel, it is reasonable to assume that our cohort indeed includes the vast majority of BVMD cases in the country. It is possible, though, that some individuals with late-onset symptoms or those who did not seek medical treatment might have been missed from our calculations; therefore, the prevalence of BVMD might be a bit higher than the one predicted here.

AR diseases are predicted to be more common in populations with high rates of consanguinity and intracommunity marriages. Hence, we expected a higher prevalence of BVMD and mainly AR bestrophinopathies among Arab Muslims (consanguinity rate, 42%) compared to Jews (consanguinity rate, ≤13.6%).^38^ According to our data, the overall prevalence of BVMD was higher among Arab Muslims (1 in 46,250 to 1 in 76,000) compared to Jews (1 in 83,600 to 1 in 145,000). Surprisingly, the rate of recessive disease only was lower than expected (14 out of 43 families). Subsequently, we compared the percentage of recessive and dominant alleles among the Israeli population to the percentage of worldwide reported alleles, and we identified a higher percentage of recessive alleles in our population (38.3% compared to 32.32%), which can be explained by the high consanguinity rates mentioned earlier.

In the current study we identified a total of 20 mutations, four of which were novel (c.967G>A, c.970G>T, c.1740-1G>C, and c.1622del), in 76 out of 134 patients with BVMD (56%). Of note, most mutations were identified using whole exome sequencing excluding the presence of large deletions or insertions. Because of the fact that the Jewish population consists of several subpopulations that immigrated from other countries, many of the disease-causing mutations can also be found in other populations, mostly European, North African, and Middle Eastern.^2^^,^^36^^,^^40^^–^^43^ Six mutations were recurrent (c.887A>G, c.908A>T, c.653G>A, c.404G>A, c.294G>C, and c.74G>A) and were identified in several families from the same origin, indicating a possible founder effect.

Analysis of the mutation type in the Israeli population revealed that most of the mutations are missense (84 alleles, which are 89.3% of all alleles). This value is lower than the worldwide one reported in the current study (90.1%) and is also lower than the worldwide percentage reported previously (92%).^28^ Most of the missense alleles worldwide (72%) and in the Israeli population (68%) are dominant. Missense mutations are predicted to result in a production of abnormal or non-functioning protein that interferes with the normal protein and causes BVMD in a dominant-negative mechanism.^28^ Five of the mutations that were reported as missense are predicted to affect splicing, alter the exonic splicing regulatory regions, and create new splice sites (c.248G>A, c.256G>A, c.704T>C, c.707A>G, and c.715G>A), and as a consequence to cause ADVIRC. These mutations are predicted (and most of them have also been proved) to produce several different transcripts, among them an allele with a missense mutation and in-frame deletion upon exon skipping.^18^ Hence, possible treatment for patients with AD BVMD or ADVIRC due to missense mutation (the vast majority of patients with Best disease) is targeted editing of the missense allele, using a CRISPR–Cas9 system, for example, to abolish the mutated allele or to turn it into a recessive one.^44^ Additional therapeutic options might be short antisense oligonucleotides, which can modify gene expression and mRNA splicing,^45^ or different RNA editing methods.^46^

An interesting feature of *BEST1* is the effect of recessive mutations. Mutations that are expected to be null, such as nonsense, splicing, small insertions, and the vast majority of deletions, always cause a recessive disease. Missense mutations are not consistent, as almost 23% have been reported so far to cause a recessive disease. Interestingly, about 10% of the missense mutations can cause either recessive or dominant disease, possibly due to the influence of other modifying genes or promoter variations. In addition, the same *BEST1* mutations can cause variable phenotypes, most of which involve the macula, but they can also be multifocal. It has been previously reported that the level of *BEST1* is higher in extramacular RPE cells compared to macular RPE cells; hence, it has been speculated that the relative insufficiency of wild-type BEST1 in the macula is the reason for the macular phenotype of BVMD.^47^ We suspect that variants in the promoter region or other modulator genes of *BEST1*, such as *OTX1*, *OTX2*, and *CRX*,^48^ might affect the level of protein expression such that expression outside the macula can be low enough to cause multifocal disease in patients with other mutations in *BEST1* but will not affect healthy retinas. Stability of the protein might also influence inheritance type or disease severity. Previous studies have identified pathogenic mutations in the N-terminus *BEST1* affecting protein stability. Mutated BEST1 proteins harboring AR mutations degrade faster, as they are recognized by the endoplasmic reticulum (ER), whereas those with AD mutations escape ER quality control and degrade at the Golgi complex.^32^ In addition, environmental factors can also affect the disease severity and age of onset.^49^

Conservation analysis revealed four conserved regions that are rich with dominant mutations. Approximately the same regions were previously described as clusters of hot spots defined for AD BVMD (AA 6–30, 80–104, 221–243, and 293–312 regions),^28^ indicating that these regions are extremely important for protein function and structure. An interesting observation is that of an intracellular region (IC3) in the C-terminus of BEST1, which is in close proximity to the transmembrane region of AAs 288 to 323, with average conservation of 94.21%. This region includes a high rate of mutations (an average of two mutations per each AA) and a low percentage of recessive mutations: four recessive mutations (1%) and five mutations that can cause recessive or dominant disease (1.2%), compared to 33% and 9% in the entire protein, respectively. An additional interesting observation is that frameshift mutations (*n* = 4) cause a dominant disease if they appear in this region but cause recessive disease if they appear elsewhere (*n* = 24). These results indicate that this region is extremely crucial for the proper function of a protein, and they are supported by other findings regarding the structure of this region.^50^

Bestrophin-1 was reported previously to act as a Ca^2^^+^-activated Cl^−^ channel gated by intracellular binding of Ca^2+^ by the C-terminal region located between AAs 312 and 323. This region is referred to as an EF hand—that is, a Ca^2^^+^-binding, helix–loop–helix structural protein domain. Two additional C-terminal domains are probably required for this Ca^2^^+^-regulated Cl^−^ channel gating. One of them is an acidic AA-rich region (AAs 293–308), which is located in the C-terminal region close to the last transmembranal domain.^50^ Experiments performed in human embryonic kidney 293 (HEK-293) cells examined *BEST1* mutations in the C-terminal regions crucial for Ca^2+^ binding (AAs 293–308 and 312–323) and revealed that mutations in these regions disrupt normal Ca^2+^ binding, causing reduction or even abolishment of the Cl^−^ current.^50^

In summary, we performed a retrospective study on 76 patients with BVMD from nine clinical centers in Israel. To our knowledge, this is the largest cohort of patients with Best disease reported to date in the Israeli population and worldwide. Based on those findings, we were able to calculate the maximum and the minimum prevalence of Best disease in the Israeli population and different subpopulations that reside in Israel. In addition, after reviewing previously published papers, it appears that mutations in *BEST1* can cause a broad clinical spectrum of macular dystrophies, which suggests a multifunctional role of the protein in the retina. Our analysis revealed several conserved regions along the protein that are very sensitive to AA alterations and where even minor missense mutations can cause AD disease. Those findings might shed light on *BEST1* regions that are crucial for its wild-type structure and function. We also evaluated the percentage of dominant and recessive alleles and percentage of different mutation types that are involved in each inheritance pattern. This information may help in evaluating the potential number of *BEST1* patients that might benefit from the development of gene augmentation therapy or RNA editing therapy for those diseases. The findings from this research will enable us and other researchers to better understand the range of mutations that cause the disease, identify crucial functional domains, and explore possible correlations between genotypes and phenotypes. Ultimately, this knowledge will contribute to unraveling the molecular mechanisms underlying BVMD and guide the development of novel treatments.

## Supplementary Material

Supplement 1
