# Supplementary material for: Best Disease: Global Mutations Review, Genotype–Phenotype Correlation, and Prevalence Analysis in the Israeli Population
Source: Invest Ophthalmol Vis Sci. 2024 Feb 27;65(2):39. doi: 10.1167/iovs.65.2.39 (PMC10910552; doi:10.1167/iovs.65.2.39)
Supplement: Supplement 1 [file iovs-65-2-39_s001.pdf]

Supplemental material

Best Disease: Global Mutations Review, Genotype-Phenotype Correlation, and Prevalence Analysis in the Israeli Population

Avigail Beryozkin[1,2]; Ifat Sher[3]; Miriam Ehrenberg[4]; Dinah Zur[5]; Hadas Newman[5]; Libe Gradstein[6]; Francis Simaan[7]; Ygal Rotenstreich[3]; Nitza Goldenberg-Cohen[8,10]; Irit Bahar[5,9]; Anat Blumenfeld[1]; Antonio Rivera[1]; Boris Rosin[1]; Iris Deitch-Harel[9]; Ido Perlman[5,10]; Hadas Mechoulam[1]; Itay Chowers[1]; Rina Leibu[11]; Tamar Ben-Yosef[10]; Eran Pras[7,12]; Eyal Banin[1]; Dror Sharon[1]; Samer Khateb[1]

[1]Department of Ophthalmology, Hadassah Medical Center, Faculty of Medicine, The Hebrew University of Jerusalem, Jerusalem, Israel.
[2]Department of Ophthalmology, University of Pittsburgh, Pittsburgh, United States.
[3]Goldschleger Eye Institute, Sheba Medical Center, Faculty of Medicine, Tel Aviv University, Tel Aviv, Israel.
[4]Ophthalmology Unit, Schneider Children's Medical Center, Faculty of Medicine, Tel Aviv University, Tel Aviv, Israel.
[5]Ophthalmology Division, Tel Aviv Medical Center, Faculty of Medicine, Tel Aviv University, Tel Aviv, Israel.
[6]Department of Ophthalmology, Soroka Medical Center and Clalit Health Services, Faculty of Health Sciences, Ben-Gurion University, Be’er Sheva, Israel.
[7]Department of Ophthalmology, Assaf Harofeh Medical Center, Zerifin, Israel.
[8]Department of Ophthalmology, Bnai Zion Medical Center, Haifa, Israel.
[9]Ophthalmology Department and Laboratory of Eye Research Felsenstein Medical Research Center, Rabin Medical Center, Petach Tikva, Israel.
[10]Rappaport Faculty of Medicine, Technion-Israel Institute of Technology, Haifa, Israel.
[11]Department of Ophthalmology, Rambam Health Care Center, Haifa, Israel.
[12]Faculty of Medicine, Tel Aviv University, Tel Aviv, Israel.

Supplementary Figures:

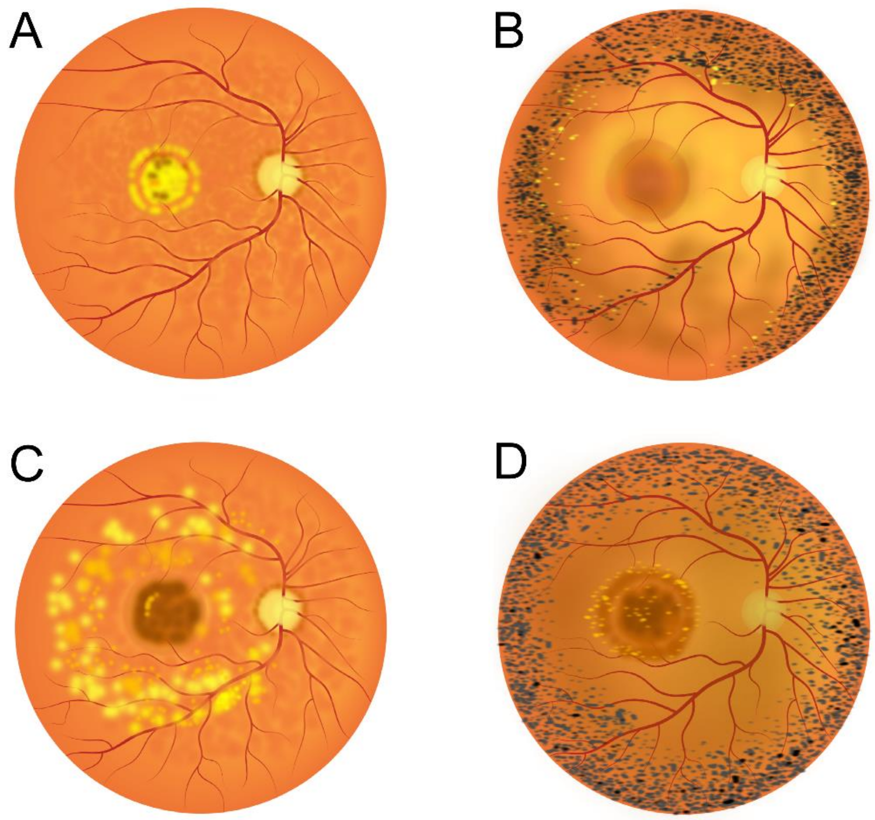

Supplementary Figure S1: Fundus appearance associated with mutations in *BEST1*. Adapted from and inspired by Singh Grewal (2021) [1]. **A.** AD Best vitelliform macular dystrophy (BVMD), characterized by "egg-yolk" macular lesions. **B.** AD vitreoretinochoroidopathy (ADVIRC), characterized by dark pigmentation in the periphery, sometimes appears together with small "egg-yolk" pigments. **C.** AR Bestrophinopathy, characterized by multifocal "egg-yolk" lesions around and beyond the macula, with or without macular involvement. **D.** *BEST1* associated RP, characterized by typical bone spicule pigmentation in the periphery, sometimes associated with small "egg-yolk" lesions in the macular area.

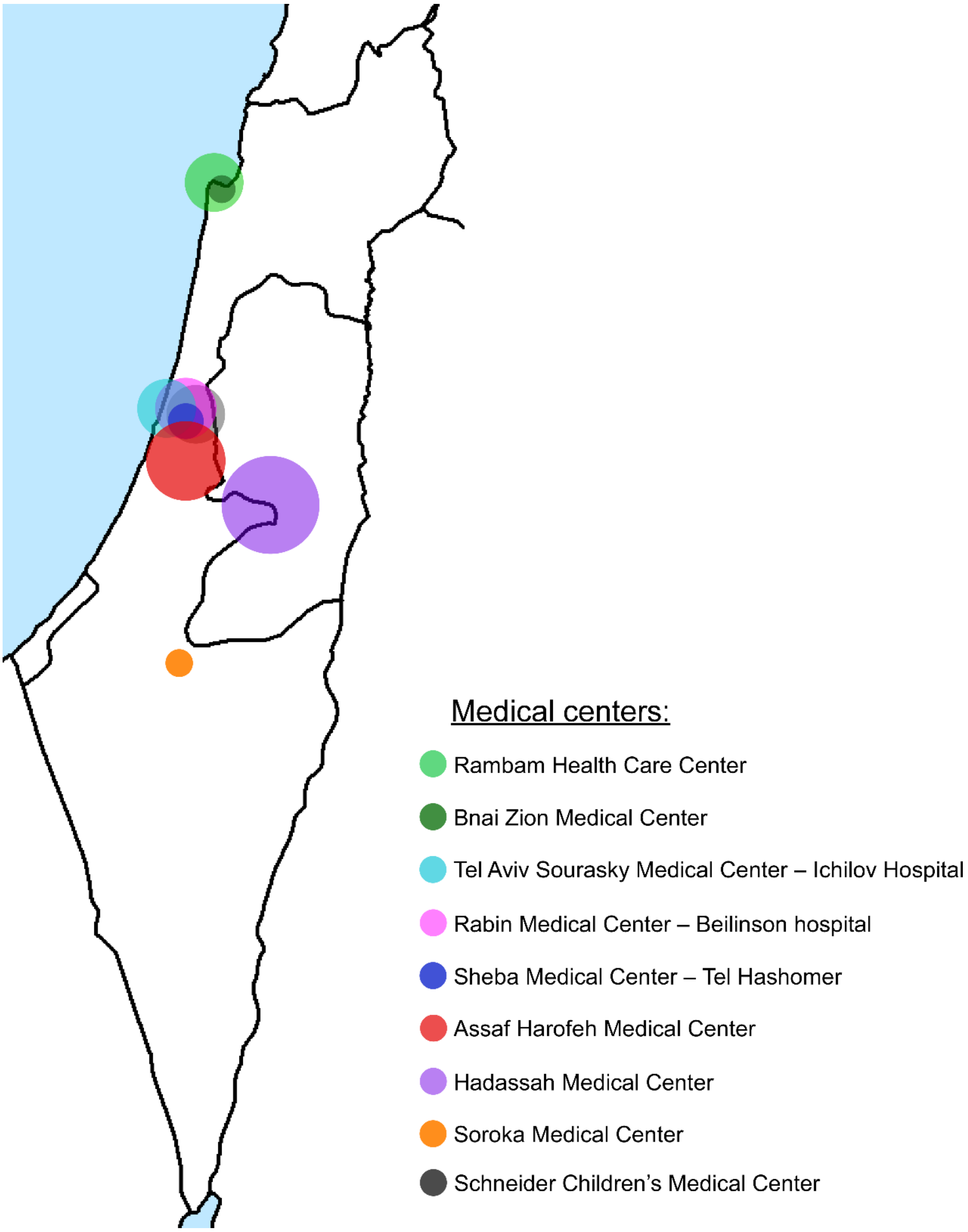

Supplementary Figure S2: The distribution of *BEST1* patients recruited by each medical center. Each medical center is represented by a circle and the size of the circle demonstrates the relative number of patients recruited in a specific medical center.

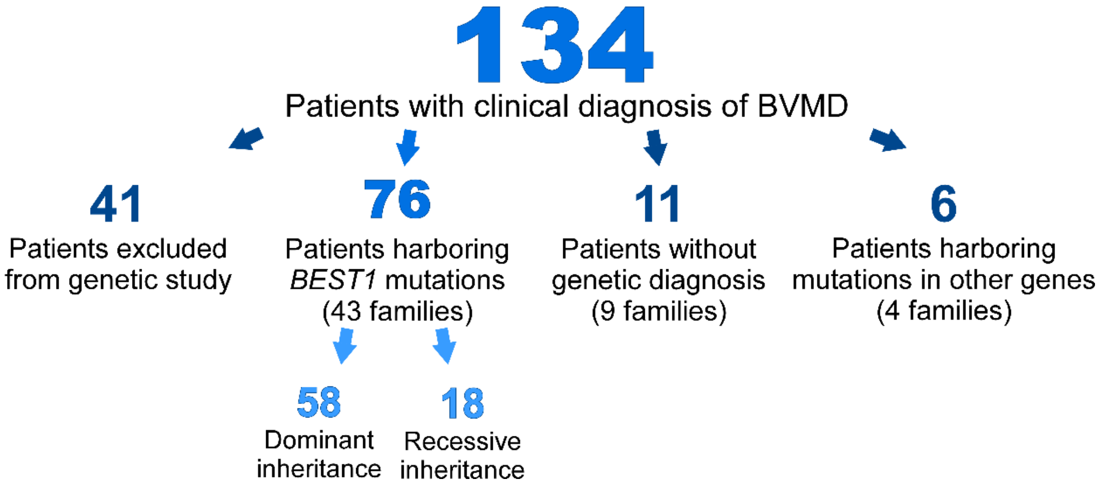

Supplementary Figure S3: Distribution of patients included in the study.

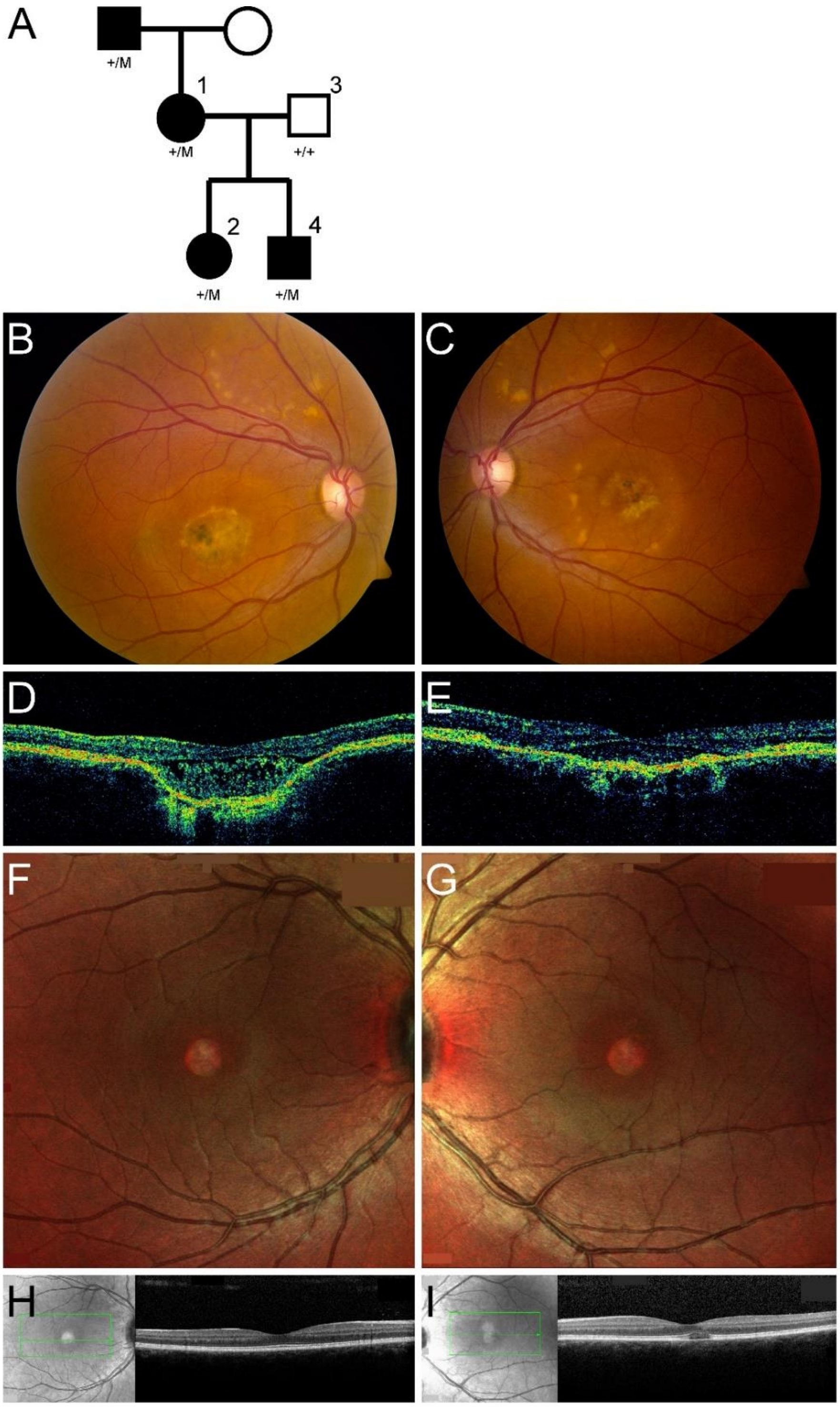

Supplementary Figure S4: Intrafamilial heterogeneity of typical AD Best disease. **A.** Pedigree of MOL0335. Squares represents males, circles represent females, black shapes represent affected family members carrying heterozygous p.Glu98Asp mutation. **(B-C)** Fundus images show scattered multifocal subrtetinal lesions and maculopathy and **(D-E)** OCT horizontal cross-sections show subfoveal scarring of the index subject MOL0335-1 at age of 40 years. (**F-G)** Fundus images of MOL0335-4 subject show classical foveal vitelliform lesion as demonstrated in **(H-I)** OCT images of at the age of 19 years. Preserved retinal layering in the RE and small subfoveal hyporeflective lesion in the LE.

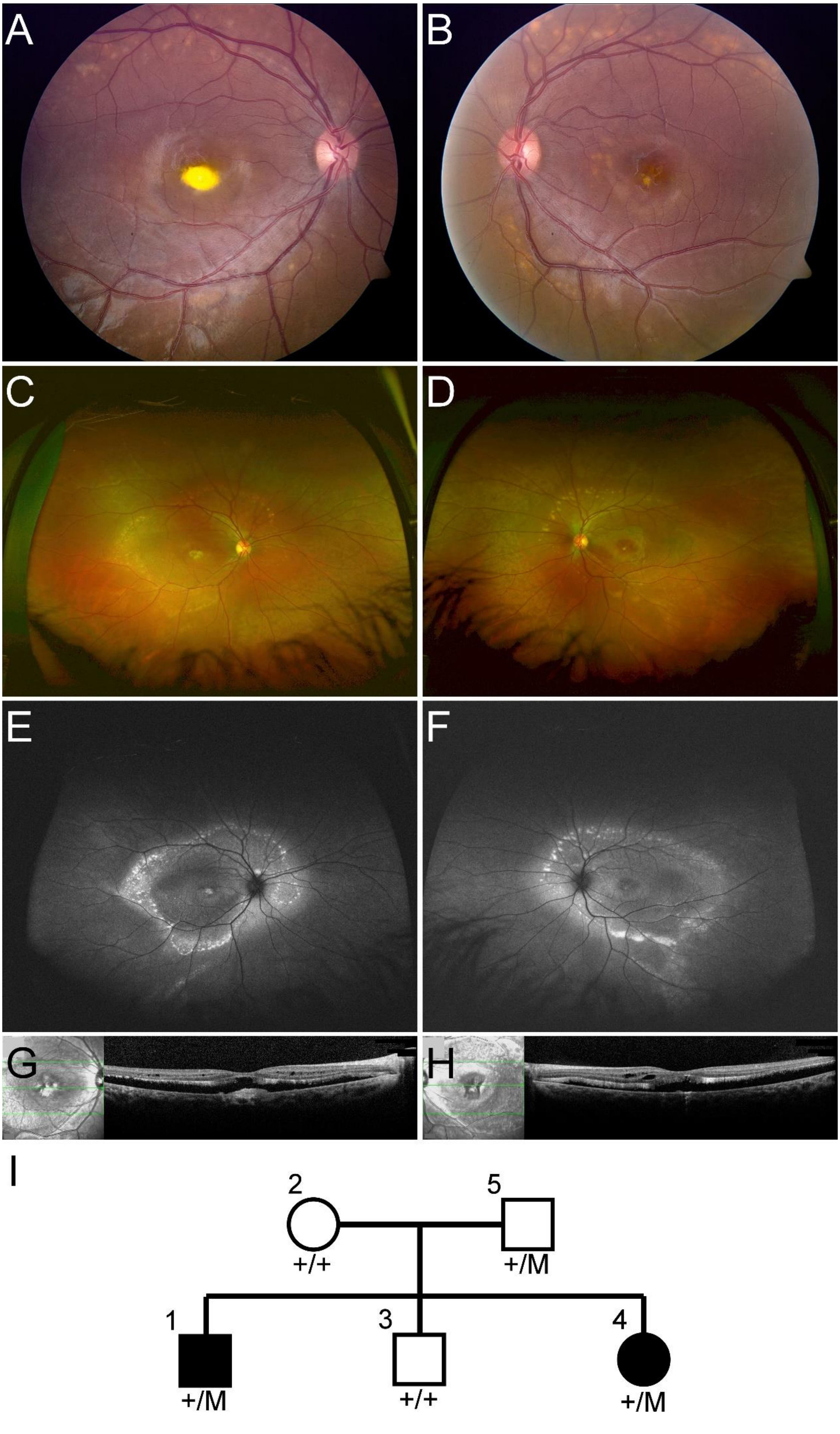
A
B
C
D
E
F
G
H
I
2
5
+/+
+/M
1
3
4
+/M
+/+
+/M

Supplementary Figure S5: Family with multifocal BVMD. **(A-B)** Representative color fundus images of MOL0443-1 harboring c.404G>A heterozygous mutation at the age of 9 years old show large yellowish vitelliform lesion in the RE and heterogeneous foveal reflex with extramacular small subretinal lesions in BE. Follow-up **(C-D)** color fundus, **(E-F)** autofluorescent and **(G-H)** OCT images of same subject at the age of 16 years old show peripheral retinal atrophy compatible with hyperfluorescent retina encroaching the macular area delimited with hyperfluorescent ring. OCT horizontal cross-sections demonstrate significant SRF and mild IRF cysts. **(I)** Pedigrees of MOL0443 family. Squares represents males, circles represent females, black shapes represent affected family members carrying c.404G>A heterozygous mutation.

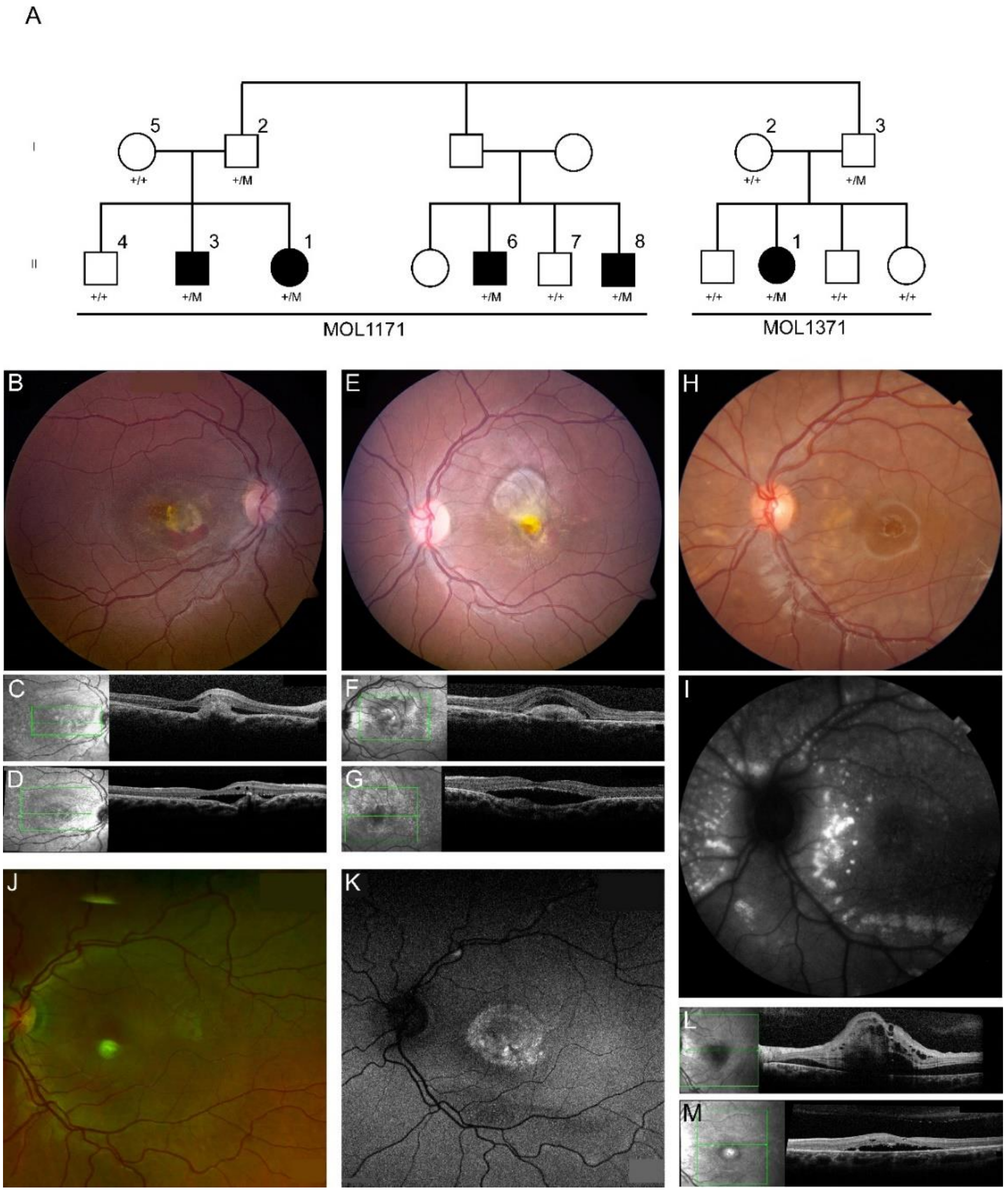

Supplementary Figure S6: AD Best disease with variable penetrance and severity. **A.** Pedigrees of MOL1171 and MOL1371 families. Squares represents males, circles represent females, black shapes represent affected family members carrying c.908A>T heterozygous mutation. **(B)** Color fundus photo (RE) of MOL1171-1 at the age of 9 years show vitelliform lesion and CNV complicated with subretinal hemorrhage. **(C-D)** OCT Horizontal cross-sections of MOL1171-1 at the age of 9 and 12 years, respectively, show CNV and subretinal fluid in the RE. **(E)** Color fundus image (LE) of MOL1171-3 at the age of 9 years demonstrate subretinal fibrosis and subretinal hemorrhage in the inferior margin. **(F)** OCT Horizontal cross-sections of MOL1171-3 at the age of 9 show CNV with SRF which disappeared at 13 years old **(G)** following intravitreal anti-VEGF injections. **(H-I)** Color fundus and FAF images (RE) of MOL1171-6 at the age of 14 years representing vitelliform lesion. (**J-K)** Color fundus and FAF images (LE) of MOL1371-1 at the age of 12 years show vitelliform lesion and heterogeneous hyperfluorescent fovea, respectively. **(L-M)** OCT Horizontal cross-sections of MOL1371-1 at the age of 11 and 13 years, respectively, showing subretinal hemorrhage transformed into subretinal scar following intravitreal anti-VEGF injections.

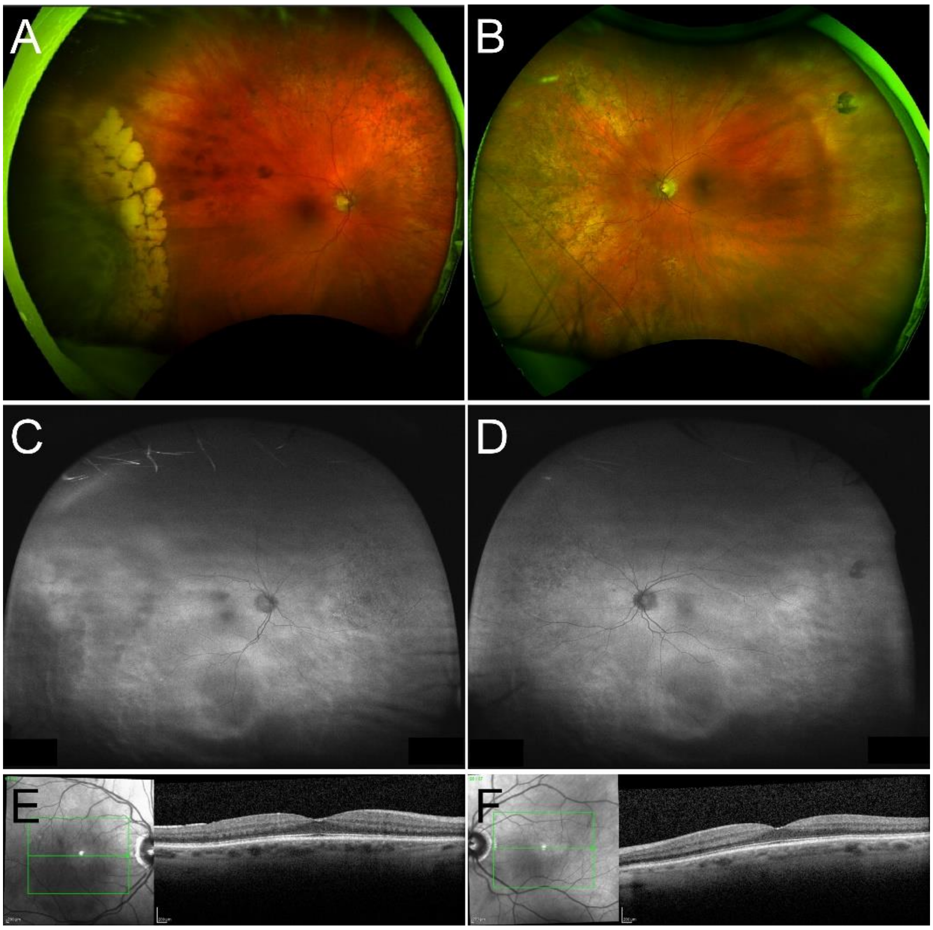

Supplementary Figure S7: Patient MOL1735-1 (72 years-old), with AD RP/AVMD due to *BEST1* heterozygous c.1415delT mutation. **(A-B)** Color fundus images demonstrating peripheral retinal atrophy and BSPs. On note, cobble-stone degeneration is seen in the temporal side of the RE. **(C-D)** FAF images of MOL1735-1 at the age of 72 years, of both eyes show diffuse heterogenous fluorescence. **(E-F)** OCT Horizontal cross-sections of MOL1735-1 at the age of 72 years old, show central preservation of the ellipsoid zone layer and photoreceptors compatible with typical RP phenotype.

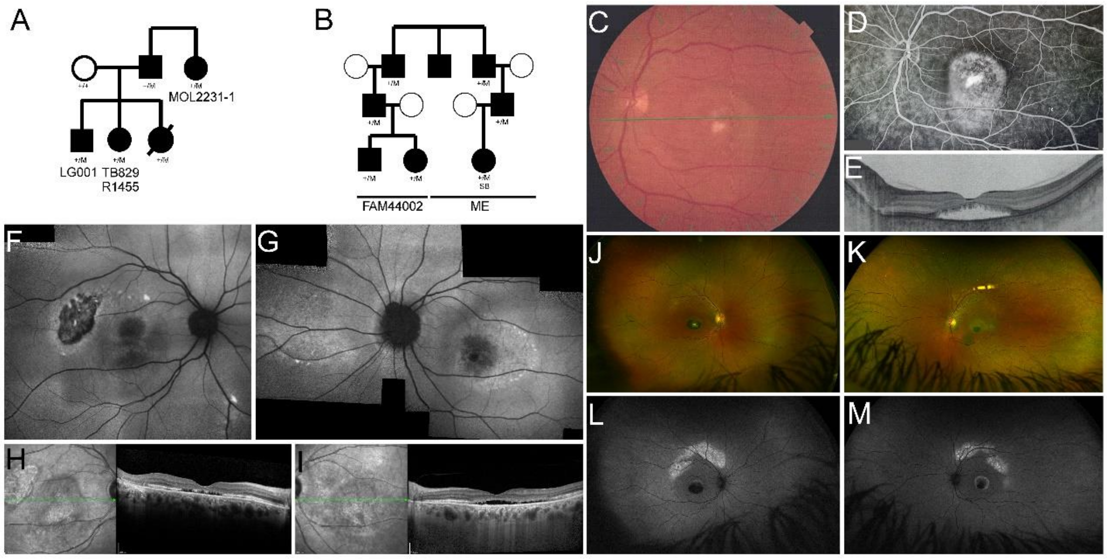

Supplementary Figure S8: Families with BVMD due to *BEST1* c.887A>G heterozygous mutation, with variable diagnosis and severity. **A-B.** Pedigrees. Squares represents males, circles represent females, black shapes represent affected family members harboring c.887A>G heterozygous mutation. **C-E.** Color fundus, FA and SD-OCT images (left eye) of LG001 at the age of 42 years with compatible findings of choroidal neovascularization and subretinal fluid. **F-I.** FAF and SD-OCT scans of BE of MOL2231-1 patient at the age of 70 years, respectively. **J-M.** Color fundus and FAF of ME-001SB, at the age of 16 years, carrying c.887A>G heterozygous mutation showing macular atrophy and scarring.

Supplementary Tables:

Supplementary Table S1: Pathogenicity evaluation of novel disease-causing variants

Mutation	Type	Frequency in control alleles	PolyPhen2	MutationTaster	SIFT	ACMG classification	Effect on protein	Population data	MAF in gnomAD
c.970G>T (p.Glu324*)	Nonsense					Likely pathogenic	PVS1	PM2	1.37e-6
c.1622del (p.Leu541fs)	Deletion					Pathogenic	PVS1	PM2	0
c.1740-1G>C (IVS10-1G>C)	Splicing					Likely pathogenic	PVS1	PM2	2.56e-6
c.967G>A (p.Asp323Asn)	Missense	0:114	probably damaging (0.971)	Disease causing (0.9999)	not tolerated	Likely pathogenic	PVS1	PM2	3.19e-5

Evolutionary conservation of the affected amino acid (p.Asp323Asn)

Human	RNLQVSLLAVDEMHQDLPRME
Chimp	RNLQVSLLAVDEMHQDLPRME
Macaca	RNLQVSLLAVDEMHQDLPRME
Dog	RSLQVSLLAVDEMHQDLPPME
Horse	RSLQVSLLAVDEMHQDLPPME
Cow	RSLQVSLLAVDEMHQDLPPME
Rabbit	RNLQVSLLSVDNMHQDLPPLE
Rat	RNLQVSLLSVDGMHHDVPPLE
Mouse	RNLQVSLLSVDGMHQNLPPME
Chicken	RNLQVSLMAVDEMHQDLPILE
Frog	RNFQVSLIAVDEMHQNLPPLE
Zebrafish	RNLQVSLLSVDEMYDLVPLVE
Fruit Fly	RNLQVSYLIVDEMHHDHPELL

Supplementary Table S2: Genetic Table

Patient ID	Gene	Diagnosis	Mutation C. (Mutation P.)	Inheritance	Zygosity	Consanguinity	Novel/ known	Previously published	Sex, YOB	Origin (ethnic group)
MOL1086-1	*BEST1*	BVMD	c.29C>T (p.Ala10Val)	AD	Het	None	[2]		M 1952	Jew (Unknown)
MOL0433-3	*BEST1*	Bestrophinopathy	c.74G>A (p.Arg25Gln)	AR	Hom	Yes	[3]		F 2000	Arab (Muslim)
MOL0433-4	*BEST1*	Bestrophinopathy	c.74G>A (p.Arg25Gln)	AR	Hom	Yes	[3]		M 2003	Arab (Muslim)
TB762/R1357	*BEST1*	Bestrophinopathy	c.74G>A (p.Arg25Gln)	AR	Hom	Yes	[3]		F 2014	Arab (Muslim)
MEZN	*BEST1*	Bestrophinopathy	c.74G>A (p.Arg25Gln)	AR	Hom	Yes	[3]		F 2015	Arab (Muslim)
SZ	*BEST1*	BVMD	c.74G>A (p.Arg25Gln)	AR	Hom	yes	[3]		F 2016	Arab (Muslim)
MOL1450-1	*BEST1*	BVMD	c.218T>A (p.Ile73Asn)	AD	Het	None	[4]		F 2008	Jew (Morrocan)
MOL1450-2	*BEST1*	BVMD	c.218T>A (p.Ile73Asn)	AD	Het	None	[4]		F 1971	Jew (Morrocan)
SP44012	*BEST1*	BVMD	c.277T>C (p.Trp93Arg)	AD	Het	None	[5]		M	Jew (Russian)
MOL0335-1	*BEST1*	BVMD	c.294G>C (p.Glu98Asp)	AD	Het	None	[6]	[7]	F 1968	Jew (Algeria)
MOL0335-2	*BEST1*	BVMD	c.294G>C (p.Glu98Asp)	AD	Het	None	[6]		M 1997	Jew (Algeria)
MOL0335-4	*BEST1*	BVMD	c.294G>C (p.Glu98Asp)	AD	Het	None	[6]		F 1994	Jew (Algeria)
MOL0335-5	*BEST1*	BVMD	c.294G>C (p.Glu98Asp)	AD	Het	None	[6]		M 1939	Jew (Algeria)
MOL1342-1/ SP44018	*BEST1*	BVMD	c.294G>C (p.Glu98Asp)	AD	Het	None	[6]		F 2008	Jew (Algeria)
MOL1342-2	*BEST1*	BVMD	c.294G>C (p.Glu98Asp)	AD	Het	None	[6]		F 1976	Jew (Algeri)
TB837/R1468	*BEST1*	AVMD	c.297C>A (p.Asn99Lys)	AD	Het	None	[8]		M 1961	Jew (Ashkenazi,

										Ukraine non Jewish)
MOL0160-1	*BEST1*	BVMD	c.324C>G (p.Ser108Arg)	AD	Het	None	[6]		F 1981	Jew (South Africa, Lita)
MOL0160-2	*BEST1*	BVMD	c.324C>G (p.Ser108Arg)	AD	Het	None	[6]		M 1955	Jew (South Africa, Lita)
MOL0443-1	*BEST1*	BVMD	c.404G>A (p.Gly135Asp)	AD	Het	None	[6]		M 1998	Jew (Iraq, Iran, Yemen)
MOL0443-4	*BEST1*	BVMD	c.404G>A (p.Gly135Asp)	AD	Het	None	[6]		F 2008	Jew (Iraq, Iran, Yemen)
MOL1567-1	*BEST1*	CRD	c.404G>A (p.Gly135Asp)	AR	Hom	Yes	[6]		M1972	Jew (Syria)
MOL1567-2	*BEST1*	CRD	c.404G>A (p.Gly135Asp)	AR	Hom	Yes	[6]		F 1971	Jew (Syria)
MOL2066-1	*BEST1*	RP	c.404G>A (p.Gly135Asp)	AR	Hom	Yes	[6]		M 1961	Jew (Iraq)
TB1209	*BEST1*	RP	c.404G>A (p.Gly135Asp)	AR	Hom	Yes	[6]		M 1948	Jew (Iraq)
TB380/R778	*BEST1*	Bestrophinopathy	c.404G>A (p.Gly135Asp) c.967G>A (p.Asp323Asn)	AR	Comp Het	None	[6]/Novel		M 2002	Jew (Morocco, Syria, Egypt, Iraq)
TB442/R872	*BEST1*	Bestrophinopathy	c.620T>A (p.Leu207His)	AR	Hom	Yes	[6]		M 1992	Arab (Muslim)
TB1332/R2123	*BEST1*	Bestrophinopathy	c.620T>A (p.Leu207His)	AR	Hom	Unknown	[6]		M	Arab (Muslim)
DZ01	*BEST1*	Bestrophinopathy	c.620T>A (p.Leu207His)	AR	Hom	Yes	[6]		M 1996	Arab (Muslim)
FAM44001-1	*BEST1*	BVMD	c.653G>A (p.Arg218His)	AD	Het	None	[4]	[7]	F 1988	Arab (Christian)
FAM44001-2	*BEST1*	BVMD	c.653G>A (p.Arg218His)	AD	Het	None	[4]		M 1965	Arab (Christian)
MOL1933-1	*BEST1*	BVMD	c.653G>A (p.Arg218His)	AD	Het	Yes	[4]		M 1982	Arab (Muslim)
MOL1512-1	*BEST1*	BVMD	c.653G>A (p.Arg218His)	AD	Het	None	[4]		M 2008	Arab (Muslim)
TB1328/R2114	*BEST1*	BVMD	c.653G>A (p.Arg218His)	AD	Het	Unknown	[4]		M 2012	Arab (Muslim)

TB429/R854	*BEST1*	BVMD	c.665G>T (p.Gly222Val)	AD	Het	None	9		M 1987	Jew (Kavkaz)
TB429/R853	*BEST1*	BVMD	c.665G>T (p.Gly222Val)	AD	Het	None	9		F 1980	Jew (Kavkaz)
TB429/R852	*BEST1*	BVMD	c.665G>T (p.Gly222Val)	AD	Het	None	9		M 1952	Jew (Kavkaz)
TB223/R544	*BEST1*	BVMD	c.874G>A (p.Glu292Lys)	AD	Het	None	10	7	M 1976	Jew (Ashkenazi)
MOL0444-1	*BEST1*	BVMD	c.887A>G (p.Asn296Ser)	AD	Het	None	4		F 1981	Jew (Bulgaria, Poland)
MOL0863-1	*BEST1*	BVMD	c.887A>G (p.Asn296Ser)	AD	Het	None	4		M 2004	Jew (Yemen, Turkey)
MOL0863-2	*BEST1*	BVMD	c.887A>G (p.Asn296Ser)	AD	Het	None	4		M 1969	Jew (Yemen, Turkey)
MOL1494-1	*BEST1*	BVMD	c.887A>G (p.Asn296Ser)	AD	Het	None	4	7	M 1991	Jew (Ashkenazi)
FAM44004-1	*BEST1*	BVMD	c.887A>G (p.Asn296Ser)	AD	Het	None	4		M 1992	Jew (Bulgaria, Turkey)
FAM44002-1	*BEST1*	BVMD	c.887A>G (p.Asn296Ser)	AD	Het	None	4		M	Jew (Turkey)
FAM44002-2	*BEST1*	BVMD	c.887A>G (p.Asn296Ser)	AD	Het	None	4		M	Jew (Turkey)
FAM44002-3	*BEST1*	BVMD	c.887A>G (p.Asn296Ser)	AD	Het	None	4		F	Jew (Turkey)
FAM44002-4	*BEST1*	BVMD	c.887A>G (p.Asn296Ser)	AD	Het	None	4		F	Jew (Turkey)
TB944/R1611	*BEST1*	BVMD	c.887A>G (p.Asn296Ser)	AD	Het	None	4		M 1974	Jew (Paras, Lebanon, Hungary, Poland)
MEGG128	*BEST1*	BVMD	c.887A>G (p.Asn296Ser)	AD	Het	None	4		M 2006	Jew (Iran, Lebanon, Hungary, Poland)
MESB	*BEST1*	BVMD	c.887A>G (p.Asn296Ser)	AD	Het	None	4		F 2003	Jew (Turkey, Ashkenazi)

MESR60	*BEST1*	BVMD	c.887A>G (p.Asn296Ser)	AD	Het	None	4	7	M 1967	Jew (Turkey)
MESSH5	*BEST1*	BVMD	c.887A>G (p.Asn296Ser)	AD	Het	None	4	7	M 1933	Jew (Turkey)
TB829/R1455	*BEST1*	BVMD	c.887A>G (p.Asn296Ser)	AD	Het	None	4		F 1983	Jew (Tunisia, Turkey)
LG001	*BEST1*	BVMD	c.887A>G (p.Asn296Ser)	AD	Het	None	4		M 1980	Jew (Tunisia, Turkey)
TB829/ MOL2213-1	*BEST1*	AVMD	c.887A>G (p.Asn296Ser)	AD	Het	None	4		F 1943	Jew (Tunisia, Turkey)
MEAS	*BEST1*	BVMD	c.905A>C (p.Asp302Ala)	AD	Het	None	11		F 2007	Jew (Turkey, Poland, Morocco)
MEADAV	*BEST1*	BVMD	c.905A>C (p.Asp302Ala)	AD	Het	None	11		M 2004	Jew (Turkey, Poland, Morocco)
MEAD	*BEST1*	BVMD	c.905A>C (p.Asp302Ala)	AD	Het	None	11	7	F 1977	Jew (Turkey, Poland, Morocco)
MOL0141-1	*BEST1*	BVMD	c.908A>T (p.Asp303Val)	AD (reduced penetrance)	Het	None	6		M 1983	Arab (Muslim)
MOL0141-2	*BEST1*	BVMD	c.908A>T (p.Asp303Val)	AD	Het	None	6		F 1991	Arab (Muslim)
MOL0141-5	*BEST1*	BVMD	c.908A>T (p.Asp303Val)	AD	Het	None	6		M 2014	Arab (Muslim)
MOL0141-6	*BEST1*	BVMD	c.908A>T (p.Asp303Val)	AD	Het	None	6		F 2013	Arab (Muslim)
MOL1171-1	*BEST1*	BVMD	c.908A>T (p.Asp303Val)	AD	Het	None	6		F 2003	Arab (Muslim)
MOL1171-3	*BEST1*	BVMD	c.908A>T (p.Asp303Val)	AD	Het	None	6		M 2004	Arab (Muslim)
MOL1171-6	*BEST1*	BVMD	c.908A>T (p.Asp303Val)	AD	Het	None	6		M 2002	Arab (Muslim)

MOL1171-8	*BEST1*	BVMD	c.908A>T (p.Asp303Val)	AD	Het	None	[6]		M 2005	Arab (Muslim)
MOL1316-1	*BEST1*	BVMD	c.908A>T (p.Asp303Val)	AD	Het	None	[6]		F 2010	Arab (Muslim)
MOL1316-3	*BEST1*	BVMD	c.908A>T (p.Asp303Val)	AD	Het	None	[6]		F 1982	Arab (Muslim)
MOL1316-4	*BEST1*	BVMD	c.908A>T (p.Asp303Val)	AD	Het	None	[6]		M 2010	Arab (Muslim)
MOL1316-5	*BEST1*	BVMD	c.908A>T (p.Asp303Val)	AD	Het	None	[6]		F 2017	Arab (Muslim)
MOL1371-1	*BEST1*	BVMD	c.908A>T (p.Asp303Val)	AD	Het	Unknown	[6]		F 2003	Arab (Muslim)
TB1008/R1703	*BEST1*	RP/ Bestrophinopathy	c.970G>T (p.Glu324*), c.1622del (p.Leu541fs)	AR	Comp Het	None	Novel/ Novel		F 1982	Jew (Ethiopian)
MOL0632-1	*BEST1*	BVMD	c.1415delT (p.Leu472Profs*10)	AR	Hom	None	[12]	[12]	F 1999	Jew (Ashkenazi)
MOL0632-7	*BEST1*	BVMD	c.1415delT (p.Leu472Profs*10)	AR	Hom	None	[12]	[12]	M 2005	Jew (Ashkenazi)
MOL1735-1	*BEST1*	RP/AVMD	c.1415delT (p.Leu472Profs*10)	AD	Het	None	[12]		F 1946	Jew (Ashkenazi)
TB917/R1573	*BEST1*	Bestrophinopathy	c.1415delT (p.Leu472Profs*10)	AR	Hom	None	[12]		M 1999	Jew (Ashkenazi)
TB85/R260	*BEST1*	Bestrophinopathy	c.1740-1G>C (IVS10-1G>C)	AR	Hom	Yes	Novel		F 1987	Arab (Muslim)
**Patients Harboring Mutations in Other Genes**										
MOL0092-1	PRPH2	AVMD	c.635G>C (p.Ser212Thr)	AD	Het	None			F 1947	Jew (Rhodus)
MOL1601-1	PRPH2	BVMD	c.441del (p.Gly148Alafs*5)	AD	Het	Unknown			F 1972	Jew (Moroccan)
MOL1726-1	PRPH2	AVMD	c.811_813del (p.Leu271del)	AD	Het	Unknown			M 1965	Jew (Moroccan)
MOL1726-2	PRPH2	AVMD	c.811_813del (p.Leu271del)	AD	Het	Unknown			M 1962	Jew (Moroccan)
MOL1726-3	PRPH2	AVMD	c.811_813del (p.Leu271del)	AD	Het	Unknown			F 1968	Jew (Moroccan)
TB850/R1485	IMPG2	AVMD	c.911G>A (p.Gly304Asp)	AD	Het	None			M 1990	Jew (Ashkenazi)

**Patients Without Genetic Diagnosis**										
MOL1368-1	Mutation not found	BVMD multifocal		Isolate		None			M 1983	Jew (Ashkenazi)
MOL1502-1	Mutation not found	BVMD		Isolate		Yes			F 2008	Arab (Muslim)
MOL1904-1	Mutation not found	AVMD		Isolate		Yes			F 1965	Jew (Moroccan)
SP44011	Mutation not found	BVMD		Isolate		None			M	Jew (Ethiopian)
EPET001-1	Mutation not found	BVMD		AD		Unknown			M	Arab (Muslim)
EPET001-2	Mutation not found	BVMD		AD		Unknown			F	Arab (Muslim)
EPFT001-3	Mutation not found	BVMD		AD		Unknown			F	Arab (Muslim)
OG023	Mutation not found	BVMD		Isolate		Unknown			M 1981	Jew (Ashkenazi)
LG003	Mutation not found	BVMD		Isolate		Yes			F 2015	Bedouin
RLIM	Mutation not found	BVMD		Isolate		Unknown			M 2011	Arab (Muslim)
DAH	Mutation not found	BVMD		Isolate		Unknown			M 2014	Jew
**Patients Excluded From The Genetic Study**			**Suspected**							
MOL0092-2		AVMD	c.635G>C (p.Ser212Thr)	AD	Het	None			F	Jew (Rhodus)
MOL0092-3		AVMD	c.635G>C (p.Ser212Thr)	AD	Het	None			F	Jew (Rhodus)
ZX		BVMD	c.324C>G (p.Ser108Arg)	AD	Het	None			M	Jew (South Africa, Lita)
AS		BVMD	c.324C>G (p.Ser108Arg)	AD	Het	None			F	Jew (South Africa, Lita)
QW		BVMD	c.324C>G (p.Ser108Arg)	AD	Het	None			M	Jew (South Africa, Lita)
MOL1342-3		BVMD	c.294G>C (p.Glu98Asp)	AD	Het	None			F	Jew (Algeria)

ER		BVMD	c.294G>C (p.Glu98Asp)	AD	Het	None			F	Jew (Algeria)
DF		BVMD	c.294G>C (p.Glu98Asp)	AD	Het	None			M	Jew (Algeria)
CV		BVMD	c.294G>C (p.Glu98Asp)	AD	Het	None			M	Jew (Algeria)
BN		BVMD	c.294G>C (p.Glu98Asp)	AD	Het	None			F	Jew (Algeria)
GH		BVMD	c.294G>C (p.Glu98Asp)	AD	Het	None			M	Jew (Algeria)
TY		BVMD	c.294G>C (p.Glu98Asp)	AD	Het	None			M	Jew (Algeria)
YU		BVMD	c.653G>A (p.Arg218His)	AD	Het	None			M	Arab (Christian)
HJ		BVMD	c.653G>A (p.Arg218His)	AD	Het	None			M	Arab (Christian)
NM		BVMD	c.653G>A (p.Arg218His)	AD	Het	None			F	Arab (Christian)
MZ		BVMD	c.887A>G (p.Asn296Ser)	AD	Het	None			M	Jew (Turkey)
MEALAT		BVMD		AD		None			F	Jew (Russia, Poland)
JK		Bestrophinopathy	c.1740-1G>C (IVS10-1G>C)	AR	Homo	yes			M	Arab (Muslim)
KL		Bestrophinopathy	c.1740-1G>C (IVS10-1G>C)	AR	Homo	yes			F	Arab (Muslim)
UI		BVMD		AD					M	Arab (Muslim)
IO		BVMD		AD					F	Arab (Muslim)
OP		BVMD		AD					F	Arab (Muslim)
QP		BVMD		AD					F	Arab (Muslim)
WO		AVMD							M	Jew
EI		AVMD							M	Jew
RU		AVMD							M	Arab (Muslim)

AL		AVMD							M	Arab (Muslim)
LG002		BVMD	c.887A>G (p.Asn296Ser)	AD	Het	None			M	Jew (Tunisia, Turkey)
FAM44004-2		BVMD	c.887A>G (p.Asn296Ser)	AD	Het	None			M	Jew (Bulgaria, Turkey)
FAM44004-3		BVMD	c.887A>G (p.Asn296Ser)	AD	Het	None			M	Jew (Bulgaria, Turkey)
FAM44004-4		BVMD	c.887A>G (p.Asn296Ser)	AD	Het	None			M	Jew (Bulgaria, Turkey)
AK		BVMD				yes			F	Bedouin
SK		BVMD				yes			M	Bedouin
SK		BVMD							M	Arab (Muslim)
DJ		BVMD							F	Arab (Muslim)
FH		BVMD							F	Arab (Muslim)
ZM		BVMD							F	Arab (Muslim)
XN		BVMD							M	Jew (Algeria)
CB		BVMD							M	Jew (North Africa)
MQ		BVMD							F	Jew (Turkey)
ZO		BVMD							M	Jew (Ashkenazi)

Supplementary Table S3: Recurrent mutations

Mutation name	Origin	# of families	# of patients
c.887A>G (p.Asn296Ser)	Jews from mixed origin (Turkey, Bulgaria, Iran and Lebanon)	9	17 (additional 5 affected family members excluded from the study)
c.908A>T (p.Asp303Val)	Arab Muslim origin from vicinity of Jerusalem	4	13
c.653G>A (p.Arg218His)	Arab Muslims and Arab Christians	4	5 (additional 5 affected family members excluded from the study)
c.404G>A (p.Gly135Asp)	Jews from mixed origin (Iran, Yemen, Iraq and Syria)	4	7
c.294G>C (p.Glu98Asp)	Algerian Jews	2	6 (additional 7 affected family members excluded from the study)
c.74G>A (p.Arg25Gln)	Arab Muslims	3	4

Supplementary Table S4: Clinical Table

Patient ID	Age (years)	BCVA (LogMAR)	Refraction	ERG			EOG*	Additional comments
				Cone Flicker 30Hz (uV, mseq)	Mixed Cone-Rod Response (a-wave, b-wave, uV)	Rod response-Blue light (b,uV)		
MOL1086-1	29	0.552	-	-	-	480	100	
MOL0433-3	20	0.096	-	106;31	218,576	431.5	106	
MOL0433-4	22	0	-	103;30	174.5,534.5	510.5	Very low amplitudes	Ichthyosis
TB762/R1357	8	0.488	1.75	-	-	-	-	strabismus, subretinal fluids
MEZN	7	0.602	2	-	-	-	-	Avastin injections
MOL1450-1	7	0.154	-	-	-	-	121	
MOL1450-2	42	ND	-	-	-	-	106.5	
SP44012		ND	-	-	-	-	-	
MOL0335-1	11	1	-	60;30 (21)	175,500	275	125	
MOL0335-2	7	0.022	-	74;28.5	239,431	310	102	
MOL0335-4	10	0	-	104.5;31	392.5,612.5	444.5	108	

MOL0335-5	70	0.698	6	44;34.5	91.5,248	160	Very low amplitudes	
MOL1342-1/ SP44018	12	0.134	1	80.5;29	125,326	329	112	
MOL1342-2	41	0.273	0.75	102.5;27.5	228.5,565	385.5	131	
TB837/R1468	49	0.067	-	104;30	183,298	126	185	
MOL0160-1	24	0.234	3.2	143;29.65	383,805.5	510.5	144	
MOL0160-2	56	0.259	3	70;32	187,347	242	104	
MOL0443-1	12	0.301	4.7	99;28 (13)	148,330	206.5	106	
MOL0443-4	8	0.585	-	-	-	-	-	
MOL1567-1		-	-	-	-	-	-	
MOL1567-2		-	-	-	-	-	-	
MOL2066	60	0.823	2.75	-	-	-	-	
TB1209	75	1.301	-	-	-	-	-	FFERG: rod-cone dystrophy, RE: macular atrophy and atrophy in the inferior periphery, LE: macular atrophy, atrophy in the inferior periphery and subretinal fluids
TB380/R778	19	0.227	0.5	-	-	-	127	
TB442/R872	23	0.455	0	35;32	181.5,204	48	105	

TB1332/R2123		-	-	-	-	-	-	
DZ01	27	0.346		Normal	Normal	Normal	Low amplitudes	Hyperopia, angle closure glaucoma
FAM44001-1	19	0.234	-	-	-	-	-	
FAM44001-2		-	-	-	-	-	-	
MOL1933-1	38	1	-	58;29 (38)	186,276	265	125	
MOL1512-1	8	0.823	3.85	38;33 (7)	92,262	205	122	
TB1328/R2114	11	0.397	-0.81	164;24.5(11)	231,369	156	-	OCT: RE subretinal thickening (scar), LE: minimal central RPE changes
Tb429/R854	29	0.221	-	56;31.5 (29)	55.5,170	85.5	190	
Tb429/R853		-	-	-	-	-	-	
Tb429/R852		-	-	-	-	-	-	
TB223/R544	28	1.124	0	55.5;30.5	244.5;460.5	236	135	
MOL0444-1	16	-	-	-	-	-	110	
MOL0863-1		-	-	-	-	-	-	
MOL0863-2		-	-	-	-	-	-	
MOL1494-1	11	0.397	-	-	-	-	110	
FAM44004	12	0.204	-	-	-	-	-	
FAM44002-1		-	-	-	-	-	-	

FAM44002-2		-	-	-	-	-	-	
FAM44002-3		-	-	-	-	-	-	
FAM44002-4		-	-	-	-	-	-	
TB944/R1611		-	-	-	-	-	-	
MEGG128	15	0	2.12	-	-	-	Very low amplitudes	
MESB	16	0.079	2.25	82.5;26.5	199,391	227.5	110	Extrafoveal involvement, outside the arcades
MESR60	30	0.747	-	96.5;54.5	404.5,456.5	196	100	Involvement of the periphery, not symmetrical
MESSH5	51	0.397	-	-	-	-	100	Atrophic
TB829/R1455	33	0.22	-	-	-	-	-	
LG001	42	0.175	-	77.5;27 (42)	232.5,316	318	135	
TB829, MOL2213-1	80	0.397	4.75	-	-	-	-	
MEAS	14	0.571	2.37	-	-	-	-	Avastin injections
MEADAV	18	0.045	-	33;34 (10)	99,333	210	127	Avastin injections
MEAD	33	0.134	-	-	-	-	137	
MOL0141-1	38	-	-	-	-	-	117	
MOL0141-2	24	0.236	-	-	-	-	123	

MOL0141-5	5	0.397	2	133;30 (4)	-	-	Very low amplitudes	
MOL0141-6	6	0.397	0.25	118;33	-	-	181	
MOL1171-1	9	0.346	-	106.5;27	246.5,382	295.5	111	
MOL1171-3	13	0.301	1	64;27 (8)	162,294	192.5	-	
MOL1171-6	14	0.096	-	-	-	-	109	
MOL1171-8		-	-	-	-	-	-	
MOL1316-1	10	0.346	5.25	72.5;30	174,481	382	135	
MOL1316-3		-	-	-	-	-	-	
MOL1316-4		-	-	-	-	-	-	
MOL1316-5		-	-	-	-	-	-	
MOL1371-1	14	0.455	7	133.5;28	235,454	302.5	135	
TB1008/R1703	36	1.337	2.25	23;35	53,97	59	-	Broad retinal involvement, not only foveal
MOL0632-1	18	0.045	2	108;29.6(8.5)	338,792	540	100	
MOL0632-7	8	0.397	1.25	131;32.2(2.5)	-	-	125	
MOL1735-1	61	0.397	-	40;28 (72)	118,218	135	133	
TB917/R1573	20	0	0	66.5;27.5	193.5,365	110.5	141	
TB85/R260	21	0.455	1.25	6;37	11,38	92	-	
SZ	5	0.726	6.5	94;27	245,495	357	-	

In case the age of ERG test is different from the age of VA test, the age appears in brackets near the ERG result. EOG, Electro-oculogram: Normal Arden light peak-to-dark through ratio (%) > 250%.

Supplementary Table S5: Worldwide Reported Mutations

Mutation C.	Mutation P.	Inheritance	Diagnosis	Location	Effect on protein	Reference	Comments
c.1-?_152+?del	Ex1del	recessive	Bestrophinopathy			[13]	
	EX1-2 del	recessive	Bestrophinopathy			[14]	
c.-37+1G>T		recessive	Bestrophinopathy			[14–18]	Also published as c.-29+1G>T
c.(-37+1_-36-1)_(152+1_153-1)del	EX1-2 del	recessive	Bestrophinopathy			[16]	The exact break point of the exon 2 deletion was not identified.
c.-37+5G>A		recessive	Bestrophinopathy			[19–21]	
c.1A>G	p.Met1?	recessive	Bestrophinopathy	Ex 1	Missense	[22]	
c.4A>G	p.Thr2Ala	dominant	BVMD	Ex 1	Missense	[23]	
c.5C>A	p.Thr2Asn	dominant	BVMD	Ex 1	Missense	[24]	
c.5C>T	p.Thr2Ile	dominant	BVMD	Ex 1	Missense	[25]	
c.5C>G	p.Thr2Ser	dominant	BVMD	Ex 1	Missense	[3,26,27]	
c.8T>A	p.Ile3Asn	dominant	BVMD	Ex 1	Missense	[28]	
c.8T>C	p.Ile3Thr	dominant	BVMD	Ex 1	Missense	[29]	
c.10A>G	p.Thr4Ala	dominant	BVMD	Ex 1	Missense	[30,31]	
c.11C>T	p.Thr4Ile	dominant	BVMD	Ex 1	Missense	[25,32–35]	
c.15C>A	p.Tyr5*	recessive	Bestrophinopathy	Ex 1	Nonsense	[36]	
c.16A>G	p.Thr6Ala	dominant	BVMD	Ex 1	Missense	[37]	
c.16A>C	p.Thr6Pro	dominant/ recessive	BVMD/Bestrophinopathy	Ex 1	Missense	[8,29,38–40]	
c.17C>G	p.Thr6Arg	dominant	BVMD	Ex 1	Missense	[9,35]	
c.17C>T	p.Thr6Ile	dominant	BVMD	Ex 1	Missense	[34,41,42]	
c.20G>A	p.Ser7Asn	dominant	BVMD	Ex 1	Missense	[23,35]	
c.25G>C	p.Val9Leu	dominant	BVMD	Ex 1	Missense	[11]	
c.25G>A	p.Val9Met	dominant	BVMD	Ex 1	Missense	[8,42–46]	
c.26T>C	p.Val9Ala	dominant	BVMD	Ex 1	Missense	[30,31,47–49]	
c.26T>G	p.Val9Gly	dominant	BVMD	Ex 1	Missense	[25]	
c.28G>A	p.Ala10Thr	dominant	BVMD	Ex 1	Missense	[5,8,31,43,50–54]	
c.29C>T	p.Ala10Val	dominant/ recessive	BVMD/Bestrophinopathy	Ex 1	Missense	[2,6,14,31,47]	
c.32A>T	p.Asn11Ile	dominant	BVMD	Ex 1	Missense	[49,55]	
c.33T>G	p.Asn11Lys	dominant	BVMD	Ex 1	Missense	[56]	
c.35C>A	p.Ala12Asp	dominant	BVMD	Ex 1	Missense	[57]	
c.37C>G	p.Arg13Gly	dominant/ recessive	BVMD/Bestrophinopathy	Ex 1	Missense	[34,58]	
c.37C>T	p.Arg13Cys	dominant/ recessive	BVMD/Bestrophinopathy	Ex 1	Missense	[20,32,42,46,49,59,60]	
c.38G>A	p.Arg13His	dominant/ recessive	BVMD/Bestrophinopathy	Ex 1	Missense	[3,26,35,61–64]	
c.43G>C	p.Gly15Arg	dominant	BVMD	Ex 1	Missense	[65]	
c.44G>A	p.Gly15Asp	dominant	BVMD	Ex 1	Missense	[25,30,34,49,58,66]	
c.47C>T	p.Ser16Phe	dominant	BVMD	Ex 1	Missense	[4,5,25,35,49,67–70]	
c.47C>A	p.Ser16Tyr	dominant	BVMD	Ex 1	Missense	[39]	
c.49T>A	p.Phe17Ile	dominant	BVMD	Ex 1	Missense	[27]	
c.50T>G	p.Phe17Cys	dominant	BVMD	Ex 1	Missense	[4,9]	
c.52dup	p.Ser18Phefs*34	recessive	Bestrophinopathy	Ex 1	Frameshift	[3]	Also published as: p.Ser19Phefs153
c.55C>T	p.Arg19Cys	dominant	BVMD	Ex 1	Missense	[71]	
c.58C>G	p.Leu20Val	dominant	BVMD	Ex 1	Missense	[3,26,72,73]	
c.61C>G	p.Leu21Val	dominant	BVMD	Ex 1	Missense	[8,74]	
c.62T>G	p.Leu21Arg	dominant	BVMD	Ex 1	Missense	[42]	
c.72G>T	p.Trp24Cys	dominant	BVMD	Ex 1	Missense	[5,9,34,43,58,75]	
c.72G>A	p.Trp24*	recessive	Bestrophinopathy	Ex 1	Nonsense	[76]	

c.73C>T	p.Arg25Trp	dominant	BVMD	Ex 1	Missense	[8,9,15,23,30,31,51,54,70,71,77–83]	
c.74G>A	p.Arg25Gln	dominant/ recessive	BVMD/Bestrophinopathy	Ex 1	Missense	[6,14,16,25,43,84]	
c.74G>T	p.Arg25Leu	dominant	BVMD	Ex 1	Missense	[27]	
c.76G>A	p.Gly26Ser	dominant	BVMD	Ex 1	Missense	[85]	
c.76G>C	p.Gly26Arg	dominant	BVMD	Ex 1	Missense	[55]	
c.80G>C	p.Ser27Thr	dominant	BVMD	Ex 1	Missense	[25,82]	
c.81C>G	p.Ser27Arg	dominant	BVMD	Ex 1	Missense	[8]	
c.82A>C	p.Ile28Leu	dominant	BVMD	Ex 1	Missense	[49]	
c.83T>A	p.Ile28Asn	recessive	Bestrophinopathy	Ex 1	Missense	[20]	
c.85T>C	p.Tyr29His	dominant	BVMD	Ex 1	Missense	[55]	
c.86A>G	p.Tyr29Cys	dominant	BVMD	Ex 1	Missense	[25,86]	
c.87C>G	p.Tyr29*	recessive	Bestrophinopathy	Ex 1	Nonsense	[87,88]	
c.88A>G	p.Lys30Glu	dominant	BVMD	Ex 1	Missense	[89]	
c.89A>G	p.Lys30Arg	dominant	BVMD	Ex 1	Missense	[9,53,58,76,90–93]	
c.90G>C	p.Lys30Asn	dominant	BVMD	Ex 1	Missense	[70]	
c.91C>A	p.Leu31Met	dominant/ recessive	BVMD/Bestrophinopathy	Ex 1	Missense	[94–96]	
c.97T>C	p.Tyr33His	recessive	Bestrophinopathy	Ex 1	Missense	[35]	
c.102C>T	p.Gly34Gly	recessive	Bestrophinopathy	Ex 1	Splicing	[14,59,64,81,91,97]	splicing- alters exonic splicing regulatory region, creates new splice site
c.103G>A	p.Glu35Lys	recessive	Bestrophinopathy	Ex 1	Missense	[71,91,95,98]	
c.113T>G	p.Ile38Ser	dominant	AVMD	Ex 1	Missense	[99]	
c.119T>C	p.Leu40Pro	recessive	Bestrophinopathy	Ex 1	Missense	[71,100]	
c.122T>C	p.Leu41Pro	dominant/ recessive	BVMD/Bestrophinopathy	Ex 1	Missense	[14,20,27,55,98,101,102]	
c.130T>C	p.Tyr44His	recessive	Bestrophinopathy	Ex 1	Missense	[35,71]	
c.139C>T	p.Arg47Cys	recessive	Bestrophinopathy	Ex 1	Missense	[70,78,103104]	
c.140G>A	p.Arg47His	dominant/ recessive	AVMD/BVMD/Bestrophinopathy	Ex 1	Missense	[8,64,67,71,73,96,105,106]	
c.155T>C	p.Leu52Pro	recessive	Bestrophinopathy	Ex 2	Missense	[64]	
c.169G>T	p.Glu57*	recessive	Bestropinopathy	Ex 2	Nonsense	[14]	
c.172_173dupCA	p.Gln58Hisfs*4	recessive	Bestrophinopathy	Ex 2	Frameshift	[107]	
c.173A>T	p.Gln58Leu	dominant	AVMD	Ex 2	Missense	[44]	
c.174_176del	p.Gln59del	recessive	Bestrophinopathy	Ex 2	Inframe	[63]	Also published as: c.174_176del (p.Q158del)
c.175_176dup	p.Gln59Hisfs*3	recessive	Bestrophinopathy	Ex 2	Frameshift	[16]	
c.176A>T	p.Gln59Leu	dominant	BVMD	Ex 2	Missense	[8]	
c.199_200del	p.Leu67Valfs*164	recessive	Bestrophinopathy	Ex 2	Frameshift	[96]	
c.205T>C	p.Cys69Arg	dominant	BVMD	Ex 2	Missense	[108]	
c.209A>G	p.Asp70Gly	recessive	Bestropinopathy	Ex 2	Missense	[109]	
c.214T>C	p.Tyr72His	dominant	BVMD	Ex 2	Missense	[57]	
c.215A>T	p.Tyr72Phe	dominant	BVMD	Ex 2	Missense	[49]	
c.217A>C	p.Ile73Leu	dominant	BVMD	Ex 2	Missense	[78]	
c.217A>T	p.Ile73Phe	dominant	BVMD	Ex 2	Missense	[11]	
c.218T>A	p.Ile73Asn	dominant	BVMD	Ex 2	Missense	[4,6,53]	
c.219C>G	p.Ile73Met	dominant	BVMD	Ex 2	Missense	[71]	
c.220C>T	p.Gln74*	recessive	Bestrophinopathy	Ex 2	Nonsense	[71]	
c.223C>T	p.Leu75Phe	dominant/ recessive	BVMD/Bestrophinopathy	Ex 2	Missense	[24]	
c.224T>C	p.Leu75Pro	dominant	BVMD	Ex 2	Missense	[3,26,49]	
c.226A>G	p.Ile76Val	dominant	BVMD	Ex 2	Missense	[45]	
c.227T>A	p.Ile76Asn	dominant	BVMD	Ex 2	Missense	[3]	
c.227T>C	p.Ile76Thr	dominant	BVMD	Ex 2	Missense	[71]	
c.228C>G	p.Ile76Met	dominant	BVMD	Ex 2	Missense	[71]	

c.229C>T	p.Pro77Ser	dominant	BVMD	Ex 2	Missense	[110]	
c.235dup	p.Ser79Phefs*153	dominant	BVMD	Ex 2	Frameshift	[3]	Also published as: c.232_233insT, 1 patient only, no segregation
c.236C>A	p.Ser79Tyr	dominant/ recessive	BVMD/Bestrophinopathy	Ex 2	Missense	[3,26]	
c.238T>G	p.Phe80Val	dominant	BVMD	Ex 2	Missense	[11]	
c.238T>A	p.Phe80Ile	dominant	BVMD	Ex 2	Missense	[111]	
c.239T>G	p.Phe80Cys	dominant	BVMD	Ex 2	Missense	[78]	
c.240C>A	p.Phe80Leu	dominant	BVMD	Ex 2	Missense	[5,9,23]	
c.240C>G	p.Phe80Leu	dominant	BVMD	Ex 2	Missense	[112]	
c.241G>A	p.Val81Met	dominant	BVMD	Ex 2	Missense	[3,23,35,45]	
c.241G>C	p.Val81Leu	dominant	BVMD	Ex 2	Missense	[26]	
c.241G>T	p.Val81Leu	recessive	Bestropinopathy	Ex 2	Missense	[3,26]	
c.244C>G	p.Leu82Val	dominant	BVMD	Ex 2	Missense	[2,29,48,72]	
c.247+2T>G	IVS2+2T>G	recessive	Bestrophinopathy	Int 2	Splicing	[62]	
c.247+2del	IVS2+2del	dominant	macular dystrophy	Int 2	Splicing	[34]	No segregation test, only one family member checked
c.248G>A	p.Gly83Asp	dominant	ADVIRC	Ex 3	Splicing	[113,114]	splicing
c.250T>G	p.Phe84Val	dominant	BVMD	Ex 3	Missense	[5]	
c.253T>C	p.Tyr85His	dominant	BVMD	Ex 3	Missense	[11,38,48,70,72,73,115]	
c.256G>A	p.Val86Met	dominant	ADVIRC	Ex 3	Splicing	[56,116–119]	splicing- alters exonic splicing regulatory region
c.263_279delTGGTCGTGACCCGCTGG	p.Leu88del17	recessive	Bestrophinopathy	Ex 3	Frameshift	[120]	
c.266T>C	p.Val89Ala	dominant	BVMD	Ex 3	Missense	[48,88,116]	
c.272C>T	p.Thr91Ile	dominant	BVMD	Ex 3	Missense	[9,30,31,47,106]	
c.274C>A	p.Arg92Ser	dominant	BVMD	Ex 3	Missense	[8,44]	
c.274C>T	p.Arg92Cys	dominant/ recessive	BVMD/Bestrophinopathy	Ex 3	Missense	[2,25,30,31,47,53,64,121]	
c.274C>G	p.Arg92Gly	dominant	BVMD	Ex 3	Missense	[30]	
c.275G>A	p.Arg92His	dominant	BVMD	Ex 3	Missense	[4,48,72]	
c.277T>C	p.Trp93Arg	dominant	BVMD	Ex 3	Missense	[105]	
c.278G>A	p.Trp93*	recessive	Bestrophinopathy	Ex 3	Nonsense	[14]	
c.278G>C	p.Trp93Ser	dominant	BVMD	Ex 3	Missense	[25,71]	
c.279G>C	p.Trp93Cys	dominant	BVMD	Ex 3	Missense	[5,38,61,122–124]	
c.281G>T	p.Trp94Leu	dominant	BVMD	Ex 3	Missense	[71]	
c.286C>G	p.Gln96Glu	dominant	BVMD	Ex 3	Missense	[91]	
c.287A>G	p.Gln96Arg	dominant	BVMD	Ex 3	Missense	[49,72,73,100,125,126]	
c.287A>T	p.Gln96Leu	dominant	BVMD	Ex 3	Missense	[3,100,127]	
c.287_298del	p.Gln96_Asn99del	recessive	Bestrophinopathy	Ex 3	Inframe	[14,17,18,59,96]	Also published as: c.107_118delAGTACGAGAACC (Gln36_Asn39del)
c.288G>C	p.Gln96His	dominant	BVMD	Ex 3	Missense	[2]	
c.289T>C	p.Tyr97His	dominant	BVMD	Ex 3	Missense	[71]	
c.291C>G	p.Tyr97*	recessive	Bestrophinopathy	Ex 3	Nonsense	[14]	
c.292G>A	p.Glu98Lys	recessive	Bestrophinopathy	Ex 3	Missense	[3,128]	
c.294G>C	p.Glu98Asp	dominant	BVMD	Ex 3	Missense	[6,49]	
c.295A>G	p.Asn99Asp	dominant/ recessive	BVMD/Bestrophinopathy	Ex 3	Missense	[34,71]	
c.295A>C	p.Asn99His	dominant	BVMD	Ex 3	Missense	[72,73]	
c.295A>T	p.Asn99Tyr	dominant	BVMD	Ex 3	Missense	[27,45]	
c.297C>A	p.Asn99Lys	dominant/ recessive	AVMD/BVMD/Bestrophinopathy	Ex 3	Missense	[8,56,129]	
c.299T>G	p.Leu100Arg	dominant	BVMD	Ex 3	Missense	[8,46]	
c.301C>A	p.Pro101Thr	dominant/ recessive	BVMD/Bestrophinopathy	Ex 3	Missense	[9,11,25,104]	
c.302C>T	p.Pro101Leu	dominant/	BVMD/Bestrophinopathy	Ex 3	Missense	[59,91,130,131]	

		recessive						
c.304T>C	p.Trp102Arg	dominant	AVMD	Ex 3	Missense	[8,44]		
c.310G>C	p.Asp104His	dominant	BVMD	Ex 3	Missense	[3,8,105,132]		
c.312C>A	p.Asp104Glu	dominant	BVMD	Ex 3	Missense	[38,48]		
c.313C>G	p.Arg105Gly	dominant	BVMD	Ex 3	Missense	[35,65,70]		
c.313C>T	p.Arg105Cys	dominant	AMD	Ex 3	Missense	[9]		
c.324C>G	p.Ser108Arg	dominant	BVMD	Ex 3	Missense	[6,25]		
c.325C>T	p.Leu109Tyr	recessive	Bestrophinopathy	Ex 3	Missense	[71]		
c.332C>T	p.Ser111Leu	recessive	Bestrophinopathy	Ex 3	Missense	[133]		
c.339C>G	p.Phe113Leu	dominant	BVMD	Ex 3	Missense	[134]		
c.343G>T	p.Glu115*	recessive	Bestrophinopathy	Ex 3	Nonsense	[71]		
c.346_355dup	p.Glu119Glyfs*116	dominant/ recessive	best/bestrophinopathy	Ex 3	Frameshift	[3,26,96]	Also published as: p.Glu115Glufs*120, c.345_346insGGCAAGGACG	
c.352G>C	p.Asp118His	recessive	Bestrophinopathy	Ex 3	Missense	[96]		
c.353A>C	p.Asp118Ala	recessive	Bestrophinopathy	Ex 3	Missense	[98]		
c.353_362dup	p.Leu123Alafs*112	recessive	Bestrophinopathy	Ex 3	Frameshift	[35]	Also published as: c.347_356dupGCAAGGACGA	
c.355G>C	p.Glu119Gln	dominant	AVMD/BVMD/AMD	Ex 3	Missense	[9,11,135,136]		
c.362G>C	p.Gly121Ala	recessive	RP	Ex 3	Missense	[137,138]		
c.380C>T	p.Thr127Met	recessive	Bestrophinopathy	Ex 3	Missense	[63]		
c.388C>A	p.Arg130Ser	recessive	Bestrophinopathy	Ex 3	Missense	[11,15,33,139]		
c.389G>T	p.Arg130Leu	recessive	Bestrophinopathy	Ex 3	Missense	[3,35,62]		
c.392A>G	p.Tyr131Cys	recessive	Bestrophinopathy	Ex 3	Missense	[106]		
c.393C>A	p.Tyr131*	recessive	Bestrophinopathy	Ex 3	Nonsense	[33]		
c.397A>G	p.Asn133Asp	recessive	Bestrophinopathy	Ex 3	Missense	[63]		
c.397A>C	p.Asn133His	recessive	Bestrophinopathy	Ex 3	Missense	[34,58]		
c.399C>G	p.Asn133Lys	dominant	BVMD	Ex 3	Missense	[9,91,93]		
c.400C>G	p.Leu134Val	recessive	Bestrophinopathy/CRD	Ex 3	Missense	[27,34,39,58,96,121,140–143]		
c.403G>A	p.Gly135Ser	dominant	BVMD	Ex 3	Missense	[2,9,45,49,90]		
c.404G>A	p.Gly135Asp	recessive	Bestrophinopathy	Ex 3	Missense	[6,59]		
c.416T>A	p.Ile139Asn	dominant	BVMD	Ex 3	Missense	[3]		
c.418C>G	p.Leu140Val	dominant/ recessive	Bestrophinopathy/RP/RD	Ex 3	Missense	[14,27,73,98,144]		
c.419T>A	p.Leu140Gln	recessive	Bestrophinopathy	Ex 3	Missense	[96]		
c.419T>C	p.Leu140Pro	dominant/ recessive	BVMD/Bestrophinopathy	Ex 3	Missense	[49,96]		
c.419T>G	p.Leu140Arg	dominant	BVMD	Ex 3	Missense	[9]		
c.421C>A	p.Arg141Ser	recessive	Best	Ex 3	Missense	[14,145,146]		
c.422G>A	p.Arg141His	dominant/ recessive	AVMD/BVMD/Bestrophinopathy	Ex 3	Missense	[8,9,14–16,20,34,46,48,56,58,73,87,90,91,95,96,98,101,103,107,115,121,130,131,143,145,147–151]		
c.422_429del	p.Arg141Glnfs*88	recessive	Bestrophinopathy	Ex 3	Frameshift	[26]		
c.424A>G	p.Ser142Gly	dominant	BVMD	Ex 3	Missense	[64]		
c.427G>T	p.Val143Phe	dominant	BVMD	Ex 3	Missense	[64]		
c.428T>C	p.Val143Ala	dominant	BVMD	Ex 3	Missense	[41,42]		
c.430A>G	p.Ser144Gly	dominant	BVMD	Ex 3	Missense	[36]		
c.431G>A	p.Ser144Asn	dominant	BVMD	Ex 3	Missense	[3,24,67]		
c.436G>A	p.Ala146Thr	recessive	Bestrophinopathy	Ex 3	Missense	[64]		
c.436G>T	p.Ala146Ser	dominant	BVMD	Ex 3	Missense	[108]		
c.436-437GCdelinsAA	p.Ala146Lys	dominant	AVMD	Ex 3	Missense	[135,136]		
c.445A>T	p.Lys149*	recessive	AVMD/AMD	Ex 3	Nonsense	[9]		
c.449G>C	p.Arg150Pro	recessive	Bestrophinopathy	Ex 3	Missense	[106]		
c.449G>T	p.Arg150Leu	recessive	Bestrophinopathy	Ex 3	Missense	[127,152]		
c.452T>G	p.Phe151Cys	recessive	Bestrophinopathy	Ex 3	Missense	[153]		
c.454C>G	p.Pro152Ala	recessive	Bestrophinopathy	Ex 3	Missense	[14,20,27,101,103]		

c.454C>T	p.Pro152Ser	recessive	Bestrophinopathy	Ex 3	Missense	[96]	
c.468C>G	p.His156Gln	recessive	Bestrophinopathy	Ex 3	Missense	[14]	
c.473G>A	p.Arg158His	dominant	BVMD	Ex 3	Missense	[3]	
c.475C>T	p.Gln159*	recessive	Bestrophinopathy	Ex 3	Nonsense	[14,107]	
c.478G>C	p.Ala160Pro	recessive	Bestrophinopathy	Ex 3	Missense	[57,81,153]	
c.488T>G	p.Met163Arg	recessive	Bestrophinopathy	Ex 4	Missense	[35,62,64,71]	
c.499G>A	p.Glu167Lys	recessive	Bestrophinopathy	Ex 4	Missense	[3]	
c.500A>G	p.Glu167Gly	recessive	Bestrophinopathy	Ex 4	Missense	[63,95,154]	
c.519delA	p.Lys173Asnfs*2	recessive	Bestrophinopathy	Ex 4	Frameshift	[35]	
c.521_522del	p.Leu174Glnfs*57	recessive	Bestrophinopathy	Ex 4	Frameshift	[155,156]	
c.524del	p.Ser175Thrfs*19	recessive	Bestrophinopathy	Ex 4	Frameshift	[96]	
c.530C>T	p.Pro177Leu	recessive	Bestrophinopathy	Ex 4	Missense	[14]	
c.532_534del	p.His178del	recessive	Bestrophinopathy	Ex 4	Inframe	[45]	
c.533A>C	p.His178Pro	recessive	Bestrophinopathy	Ex 4	Missense	[14,107]	
c.535A>G	p.Asn179Asp	dominant	BVMD	Ex 4	Missense	[25]	
c.536_538del	p.Asn179del	recessive	Bestrophinopathy	Ex 4	Inframe	[11,104,157]	Also published as: c.625delAAC (p.Asn179del)
c.544T>C	p.Trp182Arg	dominant	BVMD	Ex 4	Missense	[25]	
c.550C>T	p.Pro184Ser	recessive	Bestrophinopathy	Ex 4	Missense	[14,107]	
c.563T>C	p.Phe188Ser	recessive	Bestrophinopathy	Ex 4	Missense	[142]	
c.572T>C	p.Leu191Pro	recessive	Bestrophinopathy	Ex 4	Missense	[14,86,97,147]	
c.583_584insTGG	p.Lys194_Ala195insVal	dominant	Multifocal vitelliform dystrophy	Ex 4	Inframe	[39]	1 patient only, no segregation, father with mutation but normal fundus and EOG
c.584C>T	p.Ala195Val	dominant/ recessive	AVMD/BVMD/Bestrophiopathy/RP	Ex 4	Missense	[3,9,14,15,20,23,34,35,39,42,46,55–58,63,64,81,90,91,95,96,100,103,120,127,132,147,150,151,158–161]	
c.590_615del	p.Leu197Profs*26	recessive	Bestrophinopathy	Ex 4	Frameshift	[56,96]	
c.598C>T	p.Arg200*	recessive	Bestrophinopathy	Ex 4	Nonsense	[14,21,25,63,101,162,163]	
c.602T>C	p.Ile201Thr	dominant/ recessive	BVMD/Bestrophinopathy	Ex 4	Missense	[9,14,27,33,45,54,70,102,103,146,149,162,164]	
c.604C>T	p.Arg202Trp	dominant/ recessive	BVMD/Bestrophinopathy	Ex 4	Missense	[3,95,98]	
c.605G>A	p.Arg202Gln	dominant	BVMD	Ex 4	Missense	[165]	
c.614T>C	p.Ile205Thr	dominant	BVMD/RP	Ex 4	Missense	[144,166,167]	
c.619C>A	p.Leu207Ile	recessive	Bestrophinopathy	Ex 4	Missense	[9,168]	
c.620T>A	p.Leu207His	recessive	Bestrophinopathy	Ex 4	Missense	[6,96]	
c.621_632del	p.Gln208_Leu211del	recessive	Bestrophinopathy	Ex 4	Inframe	[103]	Also published as: Ile205del12ATCCTGCTCCAGAG
c.624G>A	p.Gln208Gln	dominant	BVMD	Ex 4	Missense	[44,55]	
c.626G>A	p.Ser209Asn	dominant	BVMD	Ex 4	Missense	[8,74]	
c.636+1G>A	IVS4+1G>A	recessive	Bestrophinopathy	Int 4	Splicing	[20,42,169]	
c.636+1G>C	IVS4+1G>C	recessive	Bestrophinopathy	Int 4	Splicing	[8,14]	
c.637-2_637del	p.Glu213del	recessive	Bestrophinopathy	Int 4	Splicing	[33]	
c.637G>A	p.Glu213Lys	recessive	Bestrophinopathy	Ex 5	Splicing	[170]	Splicing
c.638A>G	p.Glu213Gly	recessive	Bestrophinopathy	Ex 5	Missense	[15,104]	
c.647C>T	p.Thr216Ile	dominant	AVMD/AMD	Ex 5	Missense	[135,136]	
c.651G>T	p.Leu217Phe	dominant	BVMD	Ex 5	Missense	[86]	
c.652C>T	p.Arg218Cys	dominant	AVMD/BVMD	Ex 5	Missense	[2–5,9,24–27,35,41,42,49,53,55,56,58,61,63,70,72,73,77,78,90,121,125,129,140,171–173]	
c.652C>G	p.Arg218Gly	dominant	BVMD	Ex 5	Missense	[51,77,78]	
c.652C>A	p.Arg218Ser	dominant	BVMD	Ex 5	Missense	[2,8,25,44,55,108]	
c.653G>A	p.Arg218His	dominant	BVMD	Ex 5	Missense	[3–6,9,11,15,23,27,40,45,49,50,52,53,64,73,76,91,93,140,174–176]	
c.654T>G	p.Arg218Gln	dominant	BVMD	Ex 5	Missense	[43]	
c.658C>T	p.Gln220*	recessive	Bestrophinopathy	Ex 5	Nonsense	[15,20,95,162]	
c.659A>C	p.Gln220Pro	recessive	Bestrophinopathy	Ex 5	Missense	[27]	

c.662G>T	p.Cys221Phe	dominant	BVMD	Ex 5	Missense	45	
c.663T>G	p.Cys221Trp	dominant	BVMD	Ex 5	Missense	50,177	
c.665G>A	p.Gly222Glu	dominant	BVMD	Ex 5	Missense	23,140	
c.665G>T	p.Gly222Val	dominant/ recessive	BVMD/Bestrophinopathy	Ex 5	Missense	6,9,64,91	
c.670C>A	p.Leu224Met	dominant	BVMD	Ex 5	Missense	8,34,150	
c.671T>C	p.Leu224Pro	dominant	BVMD	Ex 5	Missense	9,27,46	
c.671T>A	p.Leu224Gln	recessive	Bestrophinopathy	Ex 5	Missense	98	
c.679T>A	p.Tyr227Asn	dominant	BVMD	Ex 5	Missense	9,29,38,43,91–93,178	
c.680A>G	p.Tyr227Cys	dominant	BVMD/RP	Ex 5	Missense	5,9,43,91,144,179	
c.680A>T	p.Tyr227Phe	dominant	BVMD	Ex 5	Missense	129	
c.682G>A	p.Asp228Asn	dominant	RP	Ex 5	Missense	73,144	
c.682G>C	p.Asp228His	dominant	RP	Ex 5	Missense	180	
c.682G>T	p.Asp228Tyr	dominant	RP	Ex 5	Missense	181	
c.684C>G	p.Asp228Glu	dominant/ recessive	AVMD/BVMD/Bestrophinopathy	Ex 5	Missense	42,91	
c.685T>G	p.Trp229Gly	dominant	BVMD	Ex 5	Missense	182	
c.689T>C	p.Ile230Thr	dominant	BVMD	Ex 5	Missense	31,47	
c.692G>C	p.Ser231Thr	dominant	BVMD	Ex 5	Missense	49,68,140	
c.693T>G	p.Ser231Arg	dominant	BVMD	Ex 5	Missense	74	
c.693T>A	p.Ser231Arg	dominant	BVMD	Ex 5	Missense	8	
c.695T>A	p.Ile232Asn	dominant	BVMD	Ex 5	Missense	25,129	
c.697C>G	p.Pro233Ala	recessive	Bestrophinopathy	Ex 5	Missense	115	
c.698C>A	p.Pro233Gln	dominant	BVMD	Ex 5	Missense	33,140	
c.698C>T	p.Pro233Leu	dominant	BVMD	Ex 5	Missense	3,108	
c.701T>C	p.Leu234Pro	dominant	BVMD	Ex 5	Missense	50,52,176	
c.703G>T	p.Val235Leu	dominant	BVMD	Ex 5	Missense	25,34,58,90	
c.703G>C	p.Val235Leu	dominant	BVMD	Ex 5	Missense	4	
c.703G>A	p.Val235Met	dominant	BVMD	Ex 5	Missense	43	
c.704T>C	p.Val235Ala	dominant	ADVIRC	Ex 5	Splicing	183	splicing- alters exonic splicing regulatory region
c.707A>G	p.Tyr236Cys	dominant	BVMD/ADVIRC	Ex 5	Splicing	41,116	splicing
c.709A>T	p.Thr237Ser	dominant	BVMD	Ex 5	Missense	41,129	
c.710C>G	p.Thr237Arg	dominant	BVMD	Ex 5	Missense	8,55,74,129	
c.712del	p.Gln238Argfs*3	recessive	Bestrophinopathy	Ex 5	Frameshift	34,58	
c.713_714+13del	p.Gln238Argfs*30	recessive	Bestrophinopathy	Int 5	Splicing	158	
c.715G>A	p.Val239Met	dominant	ADVIRC	Ex 6	Splicing	116,119	splicing
c.717delG	p.Val240*	recessive	Bestrophinopathy	Ex 6	Frameshift	132,184	
c.722C>A	p.Thr241Asn	dominant	BVMD	Ex 6	Missense	8,91,93	
c.724G>A	p.Val242Met	dominant	BVMD	Ex 6	Missense	23,172	
c.727G>A	p.Ala243Thr	dominant	BVMD	Ex 6	Missense	5,9,25,50,52,53,80,92,176	
c.728C>A	p.Ala243Glu	recessive	Bestrophinopathy	Ex 6	Missense	14,21,25	
c.728C>T	p.Ala243Val	dominant	AVMD/BVMD	Ex 6	Missense	5,8,15,25,27,29,30,34,41,42,44,49,55,58,70,72–74,78,91,141,151,159,185	
c.752G>A	p.Cys251Tyr	recessive	Bestrophinopathy	Ex 6	Missense	186	
c.762delG	p.Arg255Glyfs*4	recessive	Bestrophinopathy	Ex 6	Frameshift	16	
c.763C>T	p.Arg255Trp	dominant/ recessive	BVMD/Bestrophinopathy	Ex 6	Missense	3,24,35,62–64,71,81,108,132,161,184,187	
c.764G>A	p.Arg255Gln	recessive	Bestrophinopathy	Ex 6	Missense	3,62,63	
c.767A>G	p.Gln256Arg	recessive	Bestrophinopathy	Ex 6	Missense	3	
c.779delC	p.Pro260Glnfs*29	recessive	Bestrophinopathy	Ex 6	Frameshift	19,34,55,58,91,188	Also published as: c.777delC, Asn259 del1aaC, p.Pro260fsX288
c.816_818delTGT	p.Val273del	recessive	Bestrophinopathy	Ex 6	Inframe	98	
c.817G>A	p.Val273Met	recessive	Bestrophinopathy	Ex 6	Missense	63	
c.821C>G	p.Pro274Arg	recessive	Bestrophinopathy	Ex 6	Missense	21,103,133	
c.823G>A	p.Val275Ile	dominant/	BVMD/Bestrophinopathy/AMD	Ex 6	Missense	9,47,106	

		recessive					
c.828C>G	p.Phe276Leu	dominant	BVMD	Ex 6	Missense	[9]	
c.830C>T	p.Thr277Met	recessive	Bestrophinopathy	Ex 6	Missense	[62,63,71,96,98,109,127,152,189]	
c.839A>C	p.Gln280Pro	recessive	Bestrophinopathy	Ex 6	Missense	[14]	
c.842T>C	p.Phe281Ser	recessive	Bestrophinopathy	Ex 6	Missense	[62]	
c.843C>A	p.Phe281Leu	recessive	Bestrophinopathy	Ex 6	Missense	[3]	
c.848_850delTTC	p.Phe283del	recessive	Bestrophinopathy	Ex 6	Inframe	[9,21,103,130,190]	Also published as: p.Phe281del3CAGTTC
c.851A>G	p.Tyr284Cys	dominant	BVMD	Ex 6	Missense	[34,45,127,191,192]	
c.853G>A	p.Val285Ile	dominant	BVMD	Ex 6	Missense	[49]	
c.860G>A	p.Trp287*	recessive	Bestrophinopathy	Ex 6	Nonsense	[35,71,86,147]	
c.867+4G>A	IVS6+4G>A	recessive	Bestrophinopathy	Int 6	Splicing	[103]	
c.868-2A>G	IVS6-2A>G	recessive	Bestrophinopathy	Int 6	Splicing	[35,71,103]	
c.868G>A	p.Val290Met	dominant	BVMD	Ex 7	Splicing	[193]	Splicing
c.872C>T	p.Ala291Val	dominant	BVMD	Ex 7	Missense	[35]	
c.874G>A	p.Glu292Lys	dominant	BVMD	Ex 7	Missense	[6,10,49,67,68,90,173]	
c.874G>C	p.Glu292Gln	dominant	BVMD	Ex 7	Missense	[25]	
c.875A>T	p.Glu292Val	dominant	BVMD	Ex 7	Missense	[34]	
c.877C>A	p.Gln293Lys	dominant	BVMD	Ex 7	Missense	[2,52,75,130]	
c.879G>C	p.Gln293His	dominant	BVMD	Ex 7	Missense	[35,140]	
c.880C>T	p.Leu294Phe	dominant	BVMD	Ex 7	Missense	[5,188]	
c.880C>G	p.Leu294Val	dominant	BVMD	Ex 7	Missense	[11,55,140]	
c.883A>C	p.Ile295Leu	recessive	Bestrophinopathy	Ex 7	Missense	[91]	
c.883A>G	p.Ile295Val	dominant	BVMD	Ex 7	Missense	[45]	
c.883_885delATC	p.Ile295del	dominant	BVMD	Ex 7	Inframe	[78]	
c.884T>C	p.Ile295Thr	dominant	BVMD	Ex 7	Missense	[3,11,26,53,55,58,121,194]	
c.884_886delTCA	p.Ile295del	dominant/ recessive	BVMD, AVMD, Bestrophinopathy	Ex 7	Inframe	[8,15,34,43,55,56,58,91,93,121,129]	
c.886A>C	p.Asn296His	dominant	BVMD	Ex 7	Missense	[9,164]	
c.886A>G	p.Asn296Asp	dominant	BVMD	Ex 7	Missense	[3,86]	
c.887A>G	p.Asn296Ser	dominant	BVMD	Ex 7	Missense	[4–6,45,50,53,67,71–73]	
c.888C>A	p.Asn296Lys	dominant	AVMD/BVMD	Ex 7	Missense	[3,25,39,49,195]	
c.888C>G	p.Asn296Lys	dominant	BVMD	Ex 7	Missense	[25]	
c.889C>G	p.Pro297Ala	dominant	BVMD	Ex 7	Missense	[9,43,92]	
c.889C>T	p.Pro297Ser	dominant/ recessive	BVMD/Bestrophinopathy	Ex 7	Missense	[34,49,58,59,135,160,196]	
c.889C>A	p.Pro297Thr	dominant	BVMD	Ex 7	Missense	[3,24]	
c.892T>G	p.Phe298Val	dominant	BVMD	Ex 7	Missense	[54,68,70]	
c.893T>G	p.Phe298Cys	dominant	BVMD	Ex 7	Missense	[11,15,25]	
c.893T>C	p.Phe298Ser	dominant	AVMD/BVMD	Ex 7	Missense	[39,44,55,78]	
c.895G>A	p.Gly299Arg	dominant	BVMD	Ex 7	Missense	[140]	
c.895G>C	p.Gly299Arg	dominant	BVMD	Ex 7	Missense	[29]	
c.896G>C	p.Gly299Ala	dominant	BVMD	Ex 7	Missense	[29]	
c.896G>A	p.Gly299Glu	dominant	BVMD	Ex 7	Missense	[38]	
c.898G>A	p.Glu300Lys	dominant/ recessive	BVMD/Bestrophinopathy	Ex 7	Missense	[3,8,26,67,74,91,93,105,121]	
c.899A>C	p.Glu300Ala	dominant	BVMD	Ex 7	Missense	[121]	
c.900G>C	p.Glu300Asp	dominant	BVMD	Ex 7	Missense	[9,27,53,61,91,92,135,196]	
c.901G>A	p.Asp301Asn	dominant	BVMD	Ex 7	Missense	[8]	
c.902A>G	p.Asp301Gly	dominant	BVMD	Ex 7	Missense	[24,35]	
c.903T>G	p.Asp301Glu	dominant	AVMD/BVMD	Ex 7	Missense	[3,5,8,25,42,44,55,61,64,105,197–199]	
c.904G>T	p.Asp302Asn	dominant	BVMD	Ex 7	Missense	[72]	
c.904G>A	p.Asp302Asn	dominant	BVMD	Ex 7	Missense	[73]	
c.904G>C	p.Asp302His	dominant	BVMD	Ex 7	Missense	[200]	
c.904_912delGATGATGAT	p.Asp302_Asp304del	dominant	BVMD	Ex 7	Inframe	[29,39]	
c.905A>G	p.Asp302Gly	dominant	BVMD	Ex 7	Missense	[9,49]	

c.905A>T	p.Asp302Val	dominant	BVMD	Ex 7	Missense	[9]	
c.905A>C	p.Asp302Ala	dominant	BVMD	Ex 7	Missense	[11,50,52,72,73,91–93]	
c.907G>A	p.Asp303Asn	dominant	BVMD	Ex 7	Missense	[49,78]	
c.907G>T	p.Asp303Tyr	dominant	BVMD	Ex 7	Missense	[15]	
c.908A>G	p.Asp303Gly	recessive	Bestrophinopathy	Ex 7	Missense	[81]	
c.908A>T	p.Asp303Val	dominant	BVMD	Ex 7	Missense	[6]	
c.909T>A	p.Asp303Glu	dominant	BVMD	Ex 7	Missense	[200]	
c.910G>A	p.Asp304Asn	dominant	BVMD	Ex 7	Missense	[5]	
c.910_912delGAT	p.Asp304del	dominant	BVMD	Ex 7	Inframe	[9,23,93,108,132,182]	Also published as: c.901_903delGAT, p.(Asp301del)
c.911A>G	p.Asp304Gly	dominant	BVMD	Ex 7	Missense	[78,201]	
c.913T>C	p.Phe305Leu	dominant	BVMD	Ex 7	Missense	[202]	
c.914T>C	p.Phe305Ser	dominant	BVMD	Ex 7	Missense	[27,43,70,203]	
c.915T>G	p.Phe305Leu	dominant	BVMD	Ex 7	Missense	[70,202]	
c.917A>G	p.Glu306Gly	dominant	BVMD	Ex 7	Missense	[9,49,140]	
c.918G>C	p.Glu306Asp	dominant	BVMD	Ex 7	Missense	[9,23]	
c.919A>G	p.Thr307Ala	dominant	BVMD	Ex 7	Missense	[9]	
c.920C>A	p.Thr307Asn	dominant	BVMD	Ex 7	Missense	[26,49,67]	
c.920C>T	p.Thr307Ile	dominant	BVMD	Ex 7	Missense	[3,9,61,93,125]	
c.923A>G	p.Asn308Ser	dominant	BVMD	Ex 7	Missense	[140,200]	
c.924C>A	p.Asn308Lys	dominant	BVMD	Ex 7	Missense	[49]	
c.925T>C	p.Trp309Arg	dominant	BVMD	Ex 7	Missense	[5]	
c.929T>C	p.Ile310Thr	dominant	BVMD	Ex 7	Missense	[5,8,45,74]	
c.932T>G	p.Val311Gly	dominant	BVMD	Ex 7	Missense	[8,34,58,74]	
c.934G>A	p.Asp312Asn	dominant/ recessive	AVMD/BVMD/Bestrophiopathy/Macular dystrophy	Ex 7	Missense	[8,16,20,27,41,42,58,70,96,101,104,141]	
c.936C>A	p.Asp312Glu	dominant/ recessive	AVMD/Bestrophiopathy	Ex 7	Missense	[48,72,73,139]	
c.937A>G	p.Arg313Gly	dominant	BVMD	Ex 7	Missense	[27]	
c.947A>C	p.Gln316Pro	dominant	AVMD	Ex 7	Splicing	[93]	Splicing
c.948+1delG	IVS7+1delG	recessive	Bestrophinopathy	Int 7	Splicing	[81]	
c.949-1G>A	IVS7-1G>A	recessive	Bestrophinopathy	Int 7	Splicing	[3,71]	
c.949G>A	p.Val317Met	dominant/ recessive	BVMD/Bestrophinopathy	Ex 8	Splicing	[35,101]	Splicing
c.950_955del	p.Ser318_Leu319del	recessive	Bestrophinopathy	Ex 8	Inframe	[63]	
c.956T>C	p.Leu319Pro	recessive	Bestretinopathy/Macular dystrophy	Ex 8	Missense	[20,41,42,46,59]	
c.964G>A	p.Val322Met	recessive	Bestrophinopathy	Ex 8	Missense	[27,204]	
c.974T>C	p.Met325Thr	recessive	Bestrophinopathy	Ex 8	Missense	[14,90,101,149]	
c.979del	p.Gln327Argfs*42	recessive	Bestrophinopathy	Ex 8	Frameshift	[33,205]	Also published as 978del
c.1010A>G	p.Tyr337Cys	dominant	BVMD	Ex 8	Missense	[27]	
c.1014G>A	p.Trp338*	recessive	Bestrophinopathy	Ex 8	Nonsense	[14]	
c.1014_1015delinsCT	p.Trp338_Asn339delinsCysTyr	recessive	Bestrophinopathy	Ex 8	Missense	[121]	
c.1030C>T	p.Gln344*	recessive	Bestrophinopathy	Ex 8	Nonsense	[73]	
c.1037C>A	p.Pro346His	dominant	BVMD/RP	Ex 8	Missense	[3,23,105,127,138,206]	
c.1038dupC	p.Tyr347Leufs*54	recessive	Bestrophinopathy	Ex 8	Frameshift	[14,107]	
c.1066C>T	p.Arg356*	recessive	Bestrophinopathy	Ex 8	Nonsense	[14,62,63,70,91,107,162]	
c.1070C>T	p.Ala357Val	recessive	Bestrophinopathy	Ex 8	Missense	[62,127,158]	
c.1100+1G>A	IVS8+1G>A	recessive	Bestrophinopathy	Int 8	Splicing	[19,70,155]	
c.1100+1_1100+10delCAGGTGTGGC	p.Ile366fs*18	recessive	Bestrophinopathy	Int 8	Splicing	[103,148]	Also published as: Ile366delCAGGTGTGGC
c.1120dupG	p.Glu374Glyfs*27	recessive	Bestrophinopathy	Ex 9	Frameshift	[91,188]	Also published as: c.1118_1119insG
c.1193C>T	p.Ser398Phe	dominant	AMD	Ex 9	Missense	[207]	
c.1212delC	p.Arg405Glyfs*77	recessive	Bestrophinopathy	Ex 9	Frameshift	[170,208]	
c.1315C>T	p.Gln439*	recessive	Bestrophinopathy	Ex 9	Nonsense	[34]	
c.1339T>C	p.Ser447Pro	dominant	AMD	Ex 9	Missense	[207]	

c.1370C>G	p.Pro457Arg	dominant	BVMD	Ex 9	Missense	[20]	
c.1397G>C	p.Ser466Thr	dominant	BVMD	Ex 9	Missense	[49]	
c.1403C>T	p.Pro468Leu	recessive	Bestrophinopathy	Ex 9	Missense	[109]	
c.1415delT	p.Leu472ProfsX10	recessive	BVMD	Ex 9	Frameshift	[6,12]	
c.1470_1471delCA	p.His490Glnfs*24	dominant/ recessive	BVMD/ Bestrophiopathy	Ex 9	Frameshift	[14,61,97,103]	Also published as p.His490fsX514
c.1515_1518del	p.Ser506Leufs*16	dominant	AVMD	Ex 9	Frameshift	[27]	No segregation test, only one family member checked
c.1550C>G	p.Ser517*	recessive	Bestrophinopathy	Ex 9	Nonsense	[62,94]	
c.1699C>T	p.Leu567Phe	dominant/ recessive	AVMD/AMD	Ex 9	Missense	[74,135,136]	
c.1699del	p.Glu557AsnfsX52	recessive	Bestrophinopathy	Ex 9	Frameshift	[104]	
c.1739+1G>A	IVS9+1G>A	dominant	BVMD	Int 9	Splicing	[63]	No segregation test, only one family member checked
deletion of 9348 bases (61729891e61733239)		dominant	RP	Ex 10		[209]	The deletion begins within exon 10 of the *BEST1* gene and extends beyond exon 11 resulting in a frame shift causing deletion of 146aa from Best1, and extending into the adjacent ferritin heavy chain (FTH) gene on the opposite strand of DNA. Published as: H422fsX431

Supplementary Table S6: Preservation of domains and inheritance pattern of missense mutations

Start	End	#AAs	Domain	Average conservation	Mutation prevalence	# Missense mutations	# Dominant mutations	% Dominant mutations	# Recessive mutations	% Recessive mutations
1	30	30	intracellular	85.83	1.8	54	49	90.74	11	20.37
31	50	20	transmembranal	74.16	0.55	11	5	45.45	9	81.81
51	70	20	extracellular	73.33	0.3	6	3	50	3	50
71	94	24	transmembranal	92.01	1.625	39	36	92.31	6	15.38
95	234	140	intracellular	80.11	0.85	119	74	62.18	58	48.73
235	257	23	transmembranal	92.02	0.739	17	13	76.47	5	29.41
258	268	11	extracellular	79.54	0	0	0	0	0	0
269	287	19	transmembranal	87.28	0.578	11	4	36.36	8	72.72
288	323	36	intracellular	94.21	1.833	66	62	93.93	9	13.63
324	585	262	intracellular	51.49	0.057	15	7	46.66	9	60

Supplementary Table S7: Preservation of domains and disease type associated with different mutations in *BEST1*

Start	End	#AAs	Domain	Average conservation	Mutation prevalence	# of mutations	# BVMD mutations	% BVMD mutations	# Bestrophinopathy mutations	% Bestrophinopathy mutations
1	30	30	intracellular	85.83	2.033	61	49	80.32	18	29.51
31	50	20	transmembranal	74.16	0.55	11	4	36.36	9	81.81
51	70	20	extracellular	73.33	0.6	12	2	16.66	9	75
71	94	24	transmembranal	92.01	1.708	41	35	85.36	7	17.07
95	234	140	intracellular	80.11	0.971	136	72	52.94	71	52.21
235	257	23	transmembranal	92.02	0.913	21	11	52.38	9	42.85
258	268	11	extracellular	79.54	0.091	1	0	0	1	100
269	287	19	transmembranal	87.28	0.684	13	4	30.76	10	76.92
288	323	36	intracellular	94.21	2.083	75	65	86.66	18	24
324	585	262	intracellular	51.49	0.106	28	5	17.85	19	67.85

Supplementary Methods:

Clinical Examination

Clinical evaluation included a detailed family history and pedigree, a comprehensive ophthalmologic exam, EOG, full-field electroretinography (ffERG), color vision testing using the Farnsworth D-15 Panel and Ishihara tests, optical coherence tomography (OCT), color and infrared fundus photos, fundus autofluorescence (FAF) imaging and fluorescein angiography (FA), performed as previously described.[210] Briefly, ffERGs were recorded using monopolar corneal electrodes (Henkes-type, Medical Workshop B.V., Groningen, The Netherlands) and a computerized system (UTAS 3000; LKC, Gaithersburg, MD). In the dark-adapted state, a rod response to a dim blue flash and a mixed cone-rod response to a white flash were acquired. Cone responses to 30-Hz flashes of white light were acquired under a background light of 21 cd/m^2. Between 2-4 sets of responses were recorded in each condition to verify repeatability. All ERG responses were filtered at 0.3 to 500 Hz, and signal averaging was used. EOG was performed according to the International Society for Clinical Electrophysiology of Vision (ISCEV) standard using bilateral skin electrodes on both canthi, and the Arden ratio (light peak to dark trough) was derived. The average amplitude between the two eyes as measured on the earliest ERG and EOG tests performed in each of the patients was included in our analysis. OCT imaging was performed using the Zeiss Stratus OCT3 system or the Heidelberg Spectralis OCT. All scans presented are horizontal scans through the center of the fovea. Fundus autofluorescence images were obtained using a Heidelberg Retina Angiograph II (Heidelberg Engineering, Heidelberg, Germany).

Supplementary Results:

<u>Best disease patients' distribution</u>

Out of the total of 134 patients diagnosed with Best disease, 41 were not included in the genetic analysis, but were reported by their clinicians to have retinal manifestations of BVMD (22 of them belongs to families with identified BEST1 mutation and probably have the same mutation as other family members). Additional 6 patients were identified with mutations in other genes that were previously reported to cause AVMD and BVMD (PRPH2 and IMPG2)[211,212], in 11 patients mutation was not identified by the time of the end of this study. Likely pathogenic mutations in BEST1 were identified in a total of 76 patients (43 families). Distribution of the patients is presented in Supplementary Figure S3, list of all participants in this study and their genetic and clinical findings are presented in Supplementary Tables S2 and S4.

<u>Clinical Characterization of Families with *BEST1* Mutations</u>

AD Best disease with typical features

Family MOL0335 with dominant inheritance consists of three generations of affected subjects: a grandfather, his daughter and her two children who were clinically diagnosed with the typical form of Best disease, based on fundus, OCT, EOG, and ffERG findings. The index case MOL0335-1 (Mother), who was diagnosed with multifocal Best disease at the age of 10, proven by a low Arden ratio on EOG testing (115% RE, 135% LE). By the age of 36 VA deteriorated to 1 LogMAR and distinct fundoscopic lesions were present compatible with multifocal Best disease (See Supplementary Figure

S4). This led to examination of her two children who showed very mild pre-vitelliform changes at the ages of 8 and 10 with preserved VA, but EOG as well as genetic testing proved presence of disease (See Supplementary Table S2). The three patients were found to harbour heterozygous novel missense mutation, c.294G>C (p.Glu98Asp). Further genetic testing in the family revealed that the maternal grandfather (MOL0335-5) is also heterozygous for this mutation, but apparently had a relatively mild course of Best disease.

AD BVMD associated with multifocal lesions and marked subretinal and intraretinal fluid accumulation

The index case MOL443-1 is an 18-year-old male, noted to have visual impairment since age 4. The diagnosis of BVMD was made at the age of 8 years, following an EOG test which showed a severely reduced Arden ratio in BE (105% RE, 104% LE) in the presence of practically normal ffERG responses. Genetic analysis revealed a novel heterozygous mutation (c.404G>A) in *BEST1*. Following diagnosis in this index case, his young sister (MOL443-4) was found to have similar manifestations already at the age of 5 years. Interestingly, the father (MOL443-5), who is also heterozygous for the same mutation, had normal vision and normal funduscopic and OCT findings at the age of 44. The combination of clinical and genetic findings suggests an autosomal dominant BVMD with partial penetrance. However, based on the clinical phenotype, this family may suffer from ARB as copy variant sequences (CVS) or deep intronic mutations are not identified by Sanger sequencing. On ocular exam, the two siblings had normal anterior segments, no cataracts, but fundus exams revealed vitelliform lesions in the foveas together with yellow, round subretinal lesions along the superior and inferior arcades. This was accompanied by widespread subretinal (SRF) and intraretinal fluid (IRF), extending to the arcades and beyond (See Supplementary Figure S5), which proved to be refractory to treatment: in the older brother, at the age of 12 years, OCT imaging showed subretinal fluid in both eyes without clear leakage on fluorescein angiography. Later, IRF cysts appeared and he was therefore treated with topical and later systemic carbonic anhydrase inhibitors (CAI) up to 625mg per day without any improvement. In addition, treatment with one anti-VEGF injection (Bevacizumab) was attempted without effect. Recently, a trial with per os spironolactone 75 mg/day was prescribed for two months based on previous case reports which showed limited efficacy in the treatment of macular edema associated with retinal degeneration. This also did not show any effect on the extent of SRF and IRF. His visual acuity recently, at the age of 17, was 0.4 LogMAR in BE (See Supplementary Table S4). Findings in his sister were similar (See Supplementary Figure S5). Her recent VA, at the age of 8 years old, was 0.7 in the RE and 0.4 LogMAR in the LE (See Supplementary Table S4).

AD Best Disease showing variable penetrance and associated advanced cicatricial changes complicated with choroidal neovascularization

Two non-consanguineous Muslim Arab families (MOL1171 and MOL1371), which later appeared to be related, include five young affected subjects (ages 8-12 years old when first diagnosed, as shown in Supplementary Figure S6). Genetically they are all heterozygous for a novel *BEST1* mutation, c.908A>T (p.Asp303Val), which shows partial penetrance:

interestingly, the three fathers (who are brothers), are not affected while they are harboring the same mutation (See Supplementary Figure S6). MOL1171-1, who also manifests Turner Syndrome, presented at the age of 9 years with advanced BVMD that already included cicatricial changes in the macula of BE and choroidal neovascularization with subretinal hemorrhage in the RE (See Supplementary Figure S6). Her visual acuity at the time was 0.3 (RE) and 0.4 (LE) LogMAR. ffERG responses were within normal limits, while the EOG Arden ratio was severely reduced (RE-100%, LE-122%). Her brother, MOL1171-3, has ADHD but otherwise is systemically healthy. He presented at the age of 8 years with a vitelliform lesion in the RE, and cicatricial changes and subretinal hemorrhage caused CNV in the LE (See Supplementary Figure S6). His VA at the time was 0.15 in the RE and 1 in the LE (LogMAR). Electrophysiological testing was attempted, but poor compliance did not allow reliable ERG and EOG recordings. MOL1371-1 presented at the age of 11 with a pseudohypopion lesion in the RE and macular atrophy with subretinal hemorrhage in the LE. Her Arden ratio on EOG testing was severely reduced while ffERG responses were WNL, and initial VA was 0.4 LogMAR in both eyes. Thus, all affected subjects had severely advanced BVMD already in childhood accompanied by choroidal neovascularization (CNV) (See Supplementary Figure S6B-E). They were all treated with multiple intravitreal anti-VEGF injections. In patient MOL1371-1, at the age of 14, VA was relatively preserved- 0.5 in the RE and 0.15 LogMAR in the LE after 14 intravitreal Avastin injections.

AD RP/AVMD

Patient MOL1735-1 is descending from a non-consanguineous Jewish Ashkenazi family with a family history of RP. She had melanoma on her right eye long time ago with no other complains. Presented initially to examination at 72 years old, complaining of severe reduction in visual acuity (VA of 0.4 LogMAR). Fundus exam showed peripheral retinal atrophy mixed with bone-spicule pigmentations (BSPs). This patient was initially diagnosed with RP, mainly due to the typical peripheral BSPs and foveal sparing (See Supplementary Figure S7). Color vision test was normal. ffERG showed slight reduction of the mixed cone-rod b-wave responses. Rod and cone amplitudes were mildly reduced in BE with normal implicit time. Arden ratio was severely reduced in EOG (OD-non measurable, severe ptosis, OS-133%). Genetic work up identified heterozygous mutation in *BEST1* (c.1415delT), establishing the diagnosis of RP.

Typical and multifocal BVMD/ AVMD in two different families harboring same AD mutation (TB829, MOL2213-1, LG001, FAM44002, ME-001SB)

Members of those families were clinically and genetically diagnosed in different medical centers and associated to the same pedigree only during data analysis of the current article. TB829-R1455 is a 40 YO female, diagnosed with classic BVMD already at her teenage. Her last BCVA at the age of 33 was 0.69 RE and 0 LE (LogMAR).

Her brother, LG001, is 42 YO who was diagnosed with BVMD following routine eye exam at the age of 12 years. He has been asymptomatic up until recently when he developed difficulties with near vision. He has no nyctalopia but complaining of photophobia and floaters. His most recent exam reveals

BCVA of 0.17 in the right eye and 0.3 (LogMAR) in the left eye, with ametropia. Ishihara color vision test and ffERG were normal, while EOG responses were moderately reduced in the right (104%) and left eye (166%), respectively (Normal average - 250%, lower threshold - 186%). On examination, anterior segments within normal limits, and fundoscopy show round, white elevated lesion in the macula. In SD-OCT scans, discontinuity of the outer retinal layers and subretinal fluid in both eyes was seen, suspecting in choroidal neovascularization, which was confirmed by fluorescein angiography (FA) (See Supplementary Figure S8D, E). The patient has been referred to treatment by anti-VEGF injections.

Her cousin, MOL2213-1, complained of blurred central vision at older age. She was initially diagnosed with chronic central serous chorioretinopathy (CSCR) due to persistent macular subretinal fluid at age 65. She also received multiple intravitreal anti-VEGF injections for suspected choroidal neovascularization in both eyes. As the subretinal fluid did not resolve, treatment was suspended. Only following genetic testing of herself and other family members, she was diagnosed with AVMD. BCVA was 0.52 RE and 0.3 LE (logMAR) at the age of 70 years, with average refraction of +4.75 Dpt. On examination, mild lens nuclear sclerosis with pseudoexofoliation on the anterior segment, normal optic discs, atrophic changes in both maculas. Well defined atrophic area in the superotemporal margin of the macula in the RE was observed also (See Supplementary Figure S8F, G). SD-OCT showed shallow, hyporeflective subretinal fluid and overlying mixture of partial thickening and disruption of the interdigitation layer (See Supplementary Figure S8H, I). In FAF, the macula appeared hypoautofluorescent with surrounding bright hyperautofluorescent speckles. In the right eye, the additional atrophic area appeared with complete hypoautofluorescence (See Supplementary Figure S8F, G).

ME-001SB, is a 11 YO girl from another family, underwent ophthalmic examination due to central scotoma she had noticed few days ago. Several family members from that Jewish-Turkish family had previous history of BVMD (See Supplementary Figure S8B). BCVA at the age of 18 YO: 0.09 RE, 0 LE. Fundus exam showed a RE macular scar and atrophy (See Supplementary Figure S8J-M). ffERG was within normal limits and EOG Arden ratio 1.1 for BE. The visual field demonstrated small central scotoma in each eye. Genetic workup revealed *BEST1* mutation c.887A>G (p.Asn296Ser) heterozygous mutation.

References:

1. Singh Grewal S, Smith JJ, Carr A-JF. Bestrophinopathies: perspectives on clinical disease, Bestrophin-1 function and developing therapies. *Ther Adv Ophthalmol*. 2021;13. doi:10.1177/2515841421997191
2. Bakall B, Marknell T, Ingvast S, et al. The mutation spectrum of the bestrophin protein - Functional implications. *Hum Genet*. 1999;104(5). doi:10.1007/s004390050972
3. Gao FJ, Gao FJ, Gao FJ, et al. Mutation spectrum of the bestrophin-1 gene in a large Chinese cohort with bestrophinopathy. *Br J Ophthalmol*. 2020;104(6). doi:10.1136/bjophthalmol-2019-314679
4. Marchant D, Gogat K, Boutboul S, et al. Identification of novel VMD2 gene mutations in patients with best vitelliform macular dystrophy. *Hum Mutat*. 2001;17(3). doi:10.1002/humu.9
5. Alapati A, Goetz K, Suk J, et al. Molecular diagnostic testing by eyeGENE: Analysis of

patients with hereditary retinal dystrophy phenotypes involving central vision loss. *Investig Ophthalmol Vis Sci*. 2014;55(9). doi:10.1167/iovs.14-14359
6. Sharon D, Ben-Yosef T, Goldenberg-Cohen N, et al. A nationwide genetic analysis of inherited retinal diseases in Israel as assessed by the Israeli inherited retinal disease consortium (IIRDC). *Hum Mutat*. Published online 2020. doi:10.1002/humu.23903
7. Ner D Ben, Sher I, Hamburg A, et al. Chromatic pupilloperimetry for objective diagnosis of best vitelliform macular dystrophy. *Clin Ophthalmol*. 2019;13. doi:10.2147/OPTH.S191486
8. Krämer F, White K, Pauleikhoff D, et al. Mutations in the VMD2 gene are associated with juvenile-onset vitelliform macular dystrophy (Best disease) and adult vitelliform macular dystrophy but not age-related macular degeneration. *Eur J Hum Genet*. 2000;8(4). doi:10.1038/sj.ejhg.5200447
9. Lotery AJ, Munier FL, Fishman GA, et al. Allelic variation in the VMD2 gene in best disease and age-related macular degeneration. *Investig Ophthalmol Vis Sci*. 2000;41(6).
10. Sohn EH, Francis PJ, Duncan JL, et al. Phenotypic variability due to a novel Glu292Lys variation in exon 8 of the BEST1 gene causing best macular dystrophy. *Arch Ophthalmol*. 2009;127(7). doi:10.1001/archophthalmol.2009.148
11. Meunier I, Sénéchal A, Dhaenens CM, et al. Systematic screening of BEST1 and PRPH2 in juvenile and adult vitelliform macular dystrophies: A rationale for molecular analysis. *Ophthalmology*. 2011;118(6). doi:10.1016/j.ophtha.2010.10.010
12. Bitner H, Mizrahi-Meissonnier L, Griefner G, Erdinest I, Sharon D, Banin E. A homozygous frameshift mutation in BEST1 causes the classical form of best disease in an autosomal recessive mode. *Investig Ophthalmol Vis Sci*. 2011;52(8). doi:10.1167/iovs.11-7174
13. Ellingford JM, Campbell C, Barton S, et al. Validation of copy number variation analysis for next-generation sequencing diagnostics. *Eur J Hum Genet*. 2017;25(6). doi:10.1038/ejhg.2017.42
14. Casalino G, Khan KN, Armengol M, et al. Autosomal Recessive Bestrophinopathy: Clinical Features, Natural History, and Genetic Findings in Preparation for Clinical Trials. *Ophthalmology*. 2021;128(5). doi:10.1016/j.ophtha.2020.10.006
15. Maggi J, Koller S, Bähr L, et al. Long-range pcr-based ngs applications to diagnose mendelian retinal diseases. *Int J Mol Sci*. 2021;22(4). doi:10.3390/ijms22041508
16. Boon CJF, Van Den Born LI, Visser L, et al. Autosomal recessive bestrophinopathy: Differential diagnosis and treatment options. *Ophthalmology*. 2013;120(4). doi:10.1016/j.ophtha.2012.09.057
17. Turro E, Astle WJ, Megy K, et al. Whole-genome sequencing of patients with rare diseases in a national health system. *Nature*. 2020;583(7814). doi:10.1038/s41586-020-2434-2
18. Carss K, Arno G, Erwood M, et al. Comprehensive Rare Variant Analysis via Whole-Genome Sequencing to Determine the Molecular Pathology of Inherited Retinal Disease. *Am J Hum Genet*. Published online 2017. doi:10.1016/j.ajhg.2016.12.003
19. Zanolli M, Oporto JI, Verdaguer JI, et al. Genetic testing for inherited ocular conditions in a developing country. *Ophthalmic Genet*. 2020;41(1). doi:10.1080/13816810.2020.1734944
20. Birtel J, Gliem M, Herrmann P, MacLaren RE, Bolz HJ, Charbel Issa P. Peripapillary Sparing in Autosomal Recessive Bestrophinopathy. *Ophthalmol Retin*. 2020;4(5). doi:10.1016/j.oret.2019.12.008
21. Fung AT, Yzer S, Goldberg N, et al. New BEST1 mutations in autosomal recessive bestrophinopathy. *Retina*. 2015;35(4). doi:10.1097/IAE.0000000000000387
22. Shi Y, Tian J, Han Y, Oatts J, Wang N. Pathogenic role of the vitreous in angle-closure glaucoma with autosomal recessive bestrophinopathy: A case report. *BMC Ophthalmol*. 2020;20(1). doi:10.1186/s12886-020-01543-5
23. Katagiri S, Hayashi T, Ohkuma Y, et al. Mutation analysis of BEST1 in Japanese patients with Best's vitelliform macular dystrophy. *Br J Ophthalmol*. 2015;99(11). doi:10.1136/bjophthalmol-2015-306830
24. Wong RLM, Hou P, Choy KW, et al. Novel and homozygous best1 mutations in chinese patients with best vitelliform macular dystrophy. *Retina*. 2010;30(5). doi:10.1097/IAE.0b013e3181c700c1
25. Frecer V, Iarossi G, Salvetti AP, et al. Pathogenicity of new BEST1 variants identified

in Italian patients with best vitelliform macular dystrophy assessed by computational structural biology. *J Transl Med*. 2019;17(1). doi:10.1186/s12967-019-2080-3
26. Guo J, Gao F, Tang W, et al. NOVEL BEST1 MUTATIONS DETECTED by NEXT-GENERATION SEQUENCING in A CHINESE POPULATION with VITELLIFORM MACULAR DYSTROPHY. *Retina*. 2019;39(8). doi:10.1097/IAE.0000000000002183
27. Shah M, Shah M, Broadgate S, et al. Association of Clinical and Genetic Heterogeneity with BEST1 Sequence Variations. *JAMA Ophthalmol*. 2020;138(5). doi:10.1001/jamaophthalmol.2020.0666
28. Matson ME, Ly S V., Monarrez JL. Novel mutation in BEST1 associated with atypical best vitelliform dystrophy. *Optom Vis Sci*. 2015;92(8). doi:10.1097/OPX.0000000000000639
29. Boon CJF, Klevering BJ, Leroy BP, Hoyng CB, Keunen JEE, den Hollander AI. The spectrum of ocular phenotypes caused by mutations in the BEST1 gene. *Prog Retin Eye Res*. 2009;28(3). doi:10.1016/j.preteyeres.2009.04.002
30. Querques G, Zerbib J, Santacroce R, et al. Functional and clinical data of Best vitelliform macular dystrophy patients with mutations in the BEST1 gene. *Mol Vis*. 2009;15.
31. Querques G, Zerbib J, Georges A, et al. Multimodal analysis of the progression of best vitelliform macular dystrophy. *Mol Vis*. 2014;20.
32. Khan AO. PHENOTYPE-GUIDED GENETIC TESTING OF PEDIATRIC INHERITED RETINAL DISEASE IN THE UNITED ARAB EMIRATES. *Retina*. 2020;40(9). doi:10.1097/IAE.0000000000002675
33. Rodríguez-Muñoz A, Aller E, Jaijo T, et al. Expanding the Clinical and Molecular Heterogeneity of Nonsyndromic Inherited Retinal Dystrophies. *J Mol Diagnostics*. 2020;22(4). doi:10.1016/j.jmoldx.2020.01.003
34. Weisschuh N, Obermaier CD, Battke F, et al. Genetic architecture of inherited retinal degeneration in Germany: A large cohort study from a single diagnostic center over a 9-year period. *Hum Mutat*. 2020;41(9). doi:10.1002/humu.24064
35. Tian R, Yang G, Wang J, Chen Y. Screening for BEST1 gene mutations in Chinese patients with bestrophinopathy. *Mol Vis*. 2014;20.
36. Lacassagne E, Dhuez A, Rigaudière F, et al. Phenotypic variability in a French family with a novel mutation in the BEST1 gene causing multifocal best vitelliform macular dystrophy. *Mol Vis*. 2011;17.
37. Apushkin MA, Fishman GA, Taylor CM, Stone EM. Novel de novo mutation in a patient with best macular dystrophy. *Arch Ophthalmol*. 2006;124(6). doi:10.1001/archopht.124.6.887
38. Petrukhin K, Koisti MJ, Bakall B, et al. Identification of the gene responsible for best macular dystrophy. *Nat Genet*. 1998;19(3). doi:10.1038/915
39. Boon CJF, Klevering J, Den Hollander AI, et al. Clinical and genetic heterogeneity in multifocal vitelliform dystrophy. *Arch Ophthalmol*. 2007;125(8). doi:10.1001/archopht.125.8.1100
40. Booij JC, Bakker A, Kulumbetova J, et al. Simultaneous mutation detection in 90 retinal disease genes in multiple patients using a custom-designed 300-kb retinal resequencing chip. *Ophthalmology*. 2011;118(1). doi:10.1016/j.ophtha.2010.04.022
41. Gliem M, Müller PL, Birtel J, et al. Quantitative Fundus Autofluorescence and Genetic Associations in Macular, Cone, and Cone–Rod Dystrophies. In: *Ophthalmology Retina*. Vol 4. ; 2020. doi:10.1016/j.oret.2020.02.009
42. Birtel J, Eisenberger T, Gliem M, et al. Clinical and genetic characteristics of 251 consecutive patients with macular and cone/cone-rod dystrophy. *Sci Rep*. 2018;8(1). doi:10.1038/s41598-018-22096-0
43. Marquardt A, Stöhr H, Passmore LA, Krämer F, Rivera A, Weber BHF. Mutations in a novel gene, VMD2, encoding a protein of unknown properties cause juvenile-onset vitelliform macular dystrophy (Best's disease). *Hum Mol Genet*. 1998;7(9). doi:10.1093/hmg/7.9.1517
44. Renner AB, Tillack H, Kraus H, et al. Late onset is common in best macular dystrophy associated with VMD2 gene mutations. *Ophthalmology*. 2005;112(4). doi:10.1016/j.ophtha.2004.10.041
45. Cohn AC, Turnbull C, Ruddle JB, et al. Best's macular dystrophy in Australia: Phenotypic profile and identification of novel BEST1 mutations. *Eye*. 2011;25(2). doi:10.1038/eye.2010.180

46. Birtel J, Gliem M, Mangold E, et al. Next-generation sequencing identifies unexpected genotype-phenotype correlations in patients with retinitis pigmentosa. *PLoS One*. Published online 2018. doi:10.1371/journal.pone.0207958
47. Querques G, Atmani K, Bouzitou-Mfoumou R, Leveziel N, Massamba N, Souied EH. Preferential hyperacuity perimeter in best vitelliform macular dystrophy. *Retina*. 2011;31(5). doi:10.1097/IAE.0b013e3181f441c1
48. Schatz P, Bitner H, Sander B, et al. Evaluation of macular structure and function by OCT and electrophysiology in patients with vitelliform macular dystrophy due to mutations in BEST1. *Investig Ophthalmol Vis Sci*. 2010;51(9). doi:10.1167/iovs.10-5152
49. Augstburger E, Orès R, Mohand-Said S, et al. Outer Retinal Alterations Associated With Visual Outcomes in Best Vitelliform Macular Dystrophy. *Am J Ophthalmol*. 2019;208. doi:10.1016/j.ajo.2019.08.011
50. Duncker T, Greenberg JP, Ramachandran R, et al. Quantitative fundus autofluorescence and optical coherence tomography in best vitelliform macular dystrophy. *Investig Ophthalmol Vis Sci*. 2014;55(3). doi:10.1167/iovs.13-13834
51. Parodi MB, Zucchiatti I, Fasce F, Bandello F. Bilateral choroidal excavation in best vitelliform macular dystrophy. *Ophthalmic Surg Lasers Imaging Retin*. 2014;45(1). doi:10.3928/23258160-20140205-01
52. Ji C, Li Y, Kittredge A, et al. Investigation and Restoration of BEST1 Activity in Patient-derived RPEs with Dominant Mutations. *Sci Rep*. 2019;9(1). doi:10.1038/s41598-019-54892-7
53. Lima de Carvalho JR, Paavo M, Chen L, Chiang J, Tsang SH, Sparrow JR. Multimodal imaging in best vitelliform macular dystrophy. *Investig Ophthalmol Vis Sci*. 2019;60(6). doi:10.1167/iovs.19-26571
54. Hull S, Kiray G, Chiang JPW, Vincent AL. Molecular and phenotypic investigation of a New Zealand cohort of childhood-onset retinal dystrophy. *Am J Med Genet Part C Semin Med Genet*. 2020;184(3). doi:10.1002/ajmg.c.31836
55. Krämer F, Mohr N, Kellner U, Rudolph G, Weber BHF. Ten novel mutations in VMD2 associated with Best macular dystrophy (BMD). *Hum Mutat*. 2003;22(5). doi:10.1002/humu.9189
56. Nachtigal AL, Milenkovic A, Brandl C, et al. Mutation-dependent pathomechanisms determine the phenotype in the bestrophinopathies. *Int J Mol Sci*. 2020;21(5). doi:10.3390/ijms21051597
57. Chen TC, Huang DS, Lin CW, et al. Genetic characteristics and epidemiology of inherited retinal degeneration in Taiwan. *npj Genomic Med*. 2021;6(1). doi:10.1038/s41525-021-00180-1
58. Nowomiejska K, Nasser F, Stingl K, et al. Disease expression caused by different variants in the BEST1 gene: genotype and phenotype findings in bestrophinopathies. *Acta Ophthalmol*. 2022;100(3). doi:10.1111/aos.14958
59. Khojasteh H, Azarmina M, Ebrahimiadib N, et al. Autosomal Recessive Bestrophinopathy: Clinical and Genetic Characteristics of Twenty-Four Cases. *J Ophthalmol*. 2021;2021. doi:10.1155/2021/6674290
60. Pappalardo J, Heath Jeffery RC, Thompson JA, et al. A novel phenotype in a family with autosomal dominant retinal dystrophy due to c.1430A > G in retinoid isomerohydrolase (RPE65) and c.37C > T in bestrophin 1 (BEST1). *Doc Ophthalmol*. 2021;143(1). doi:10.1007/s10633-021-09819-x
61. Caldwell GM, Kakuk LE, Griesinger IB, et al. Bestrophin gene mutations in patients with best vitelliform macular dystrophy. *Genomics*. 1999;58(1). doi:10.1006/geno.1999.5808
62. Zhong Y, Guo X, Xiao H, et al. Flat anterior chamber after trabeculectomy in secondary angle-closure glaucoma with BEST1 gene mutation: Case series. *PLoS One*. 2017;12(1). doi:10.1371/journal.pone.0169395
63. Luo J, Lin M, Guo X, et al. Novel BEST1 mutations and special clinical characteristics of autosomal recessive bestrophinopathy in Chinese patients. *Acta Ophthalmol*. 2019;97(3). doi:10.1111/aos.13994
64. Gao T, Tian C, Hu Q, et al. Clinical and mutation analysis of patients with best vitelliform macular dystrophy or autosomal recessive bestrophinopathy in Chinese population. *Biomed Res Int*. 2018;2018. doi:10.1155/2018/4582816
65. Glavač D, Jarc-Vidmar M, Vrabec K, Ravnik-Glavač M, Fakin A, Hawlina M. Clinical

and genetic heterogeneity in Slovenian patients with BEST disease. *Acta Ophthalmol*. 2016;94(8). doi:10.1111/aos.13202
66. Kaden TR, Tan ACS, Feiner L, Freund KB. Unilateral best disease: A case report. *Retin Cases Br Reports*. 2017;11(1). doi:10.1097/ICB.0000000000000431
67. Liu J, Zhang Y, Xuan Y, Liu W, Wang M. Novel BEST1 Mutations and Special Clinical Features of Best Vitelliform Macular Dystrophy. *Ophthalmic Res*. 2016;56(4). doi:10.1159/000444681
68. Arora R, Khan K, Kasilian ML, et al. Unilateral BEST1-Associated Retinopathy. *Am J Ophthalmol*. 2016;169. doi:10.1016/j.ajo.2016.05.024
69. de Souza CPR, Mello LGM, Gomez F, Morizot E. Best vitelliform macular dystrophy in a large Brazilian family. *Int J Retin Vitr*. 2019;5(1). doi:10.1186/s40942-019-0156-0
70. Khan KN, Islam F, Holder GE, et al. Normal Electrooculography in Best Disease and Autosomal Recessive Bestrophinopathy. *Retina*. 2018;38(2). doi:10.1097/IAE.0000000000001523
71. Tian L, Sun T, Xu K, Zhang X, Peng X, Li Y. Screening of BEST1 gene in a chinese cohort with best vitelliform macular dystrophy or autosomal recessive bestrophinopathy. *Investig Ophthalmol Vis Sci*. 2017;58(9). doi:10.1167/iovs.17-21999
72. Bitner H, Schatz P, Mizrahi-Meissonnier L, Sharon D, Rosenberg T. Frequency, genotype, and clinical spectrum of best vitelliform macular dystrophy: Data from a national center in Denmark. *Am J Ophthalmol*. 2012;154(2). doi:10.1016/j.ajo.2012.02.036
73. Jespersgaard C, Fang M, Bertelsen M, et al. Molecular genetic analysis using targeted NGS analysis of 677 individuals with retinal dystrophy. *Sci Rep*. Published online 2019. doi:10.1038/s41598-018-38007-2
74. White K, Marquardt A, Weber BHF. VMD2 mutations in vitelliform macular dystrophy (Best disease) and other maculopathies. *Hum Mutat*. 2000;15(4). doi:10.1002/(SICI)1098-1004(200004)15:4<301::AID-HUMU1>3.0.CO;2-N
75. Chacon-Camacho OF, Camarillo-Blancarte L, Zenteno JC. OCT findings in young asymptomatic subjects carrying familial BEST1 gene mutations. *Ophthalmic Genet*. 2011;32(1). doi:10.3109/13816810.2010.524906
76. Consugar MB, Navarro-Gomez D, Place EM, et al. Panel-based genetic diagnostic testing for inherited eye diseases is highly accurate and reproducible, and more sensitive for variant detection, than exome sequencing. *Genet Med*. 2015;17(4). doi:10.1038/gim.2014.172
77. Sodi A, Passerini I, Simonelli F, Testa F, Menchini U, Torricelli F. A novel mutation in the VMD2 gene in an Italian family with Best maculopathy. *J Fr Ophtalmol*. 2007;30(6). doi:10.1016/S0181-5512(07)89667-7
78. Sodi A, Passerini I, Murro V, et al. BEST1 sequence variants in Italian patients with vitelliform macular dystrophy. *Mol Vis*. 2012;18.
79. Sodi A, Murro V, Caporossi O, et al. Long-Term Results of Photodynamic Therapy for Choroidal Neovascularization in Pediatric Patients with Best Vitelliform Macular Dystrophy. *Ophthalmic Genet*. 2015;36(2). doi:10.3109/13816810.2015.1009121
80. Sodi A, Mucciolo DP, Giorgio D, et al. Clinical and molecular findings in patients with pattern dystrophy. *Ophthalmic Genet*. 2021;42(5). doi:10.1080/13816810.2021.1938140
81. Nakanishi A, Ueno S, Hayashi T, et al. Clinical and Genetic Findings of Autosomal Recessive Bestrophinopathy in Japanese Cohort. *Am J Ophthalmol*. 2016;168. doi:10.1016/j.ajo.2016.04.023
82. Bernardis I, Chiesi L, Tenedini E, et al. Unravelling the Complexity of Inherited Retinal Dystrophies Molecular Testing: Added Value of Targeted Next-Generation Sequencing. *Biomed Res Int*. 2016;2016. doi:10.1155/2016/6341870
83. Audo I, Bujakowska KM, Léveillard T, et al. Development and application of a next-generation-sequencing (NGS) approach to detect known and novel gene defects underlying retinal diseases. *Orphanet J Rare Dis*. 2012;7(1). doi:10.1186/1750-1172-7-8
84. Ehrenberg M, Weiss S, Orenstein N, Goldenberg-Cohen N, Ben-Yosef T. The co-occurrence of rare non-ocular phenotypes in patients with inherited retinal degenerations. *Mol Vis*. 2019;25.
85. Campa C, Parmeggiani F, Spena R, Ognibene D, Passerini I, Gualandi F. A novel mutation of BEST1 gene in Best disease. *Eur J Ophthalmol*. 2021;31(3).

doi:10.1177/1120672120920536
86. Downs K, Zacks DN, Caruso R, et al. Molecular testing for hereditary retinal disease as part of clinical care. *Arch Ophthalmol*. 2007;125(2). doi:10.1001/archopht.125.2.252
87. Schatz P, Klar J, Andréasson S, Ponjavic V, Dahl N. Variant phenotype of Best vitelliform macular dystrophy associated with compound heterozygous mutations in VMD2. *Ophthalmic Genet*. 2006;27(2). doi:10.1080/13816810600677990
88. Eksandh L, Bakall B, Bauer B, Wadelius C, Andréasson S. Best's vitelliform macular dystrophy caused by a new mutation (val89ala) in the vmd2 gene. *Ophthalmic Genet*. 2001;22(2). doi:10.1076/opge.22.2.107.2226
89. Yang S, Li Z, Cheng W, et al. BEST1 novel mutation causes Bestrophinopathies in six families with distinct phenotypic diversity. *Mol Genet Genomic Med*. 2023;11(1). doi:10.1002/mgg3.2095
90. Holtan JP, Selmer KK, Heimdal KR, Bragadóttir R. Inherited retinal disease in Norway – a characterization of current clinical and genetic knowledge. *Acta Ophthalmol*. 2020;98(3). doi:10.1111/aos.14218
91. Stone EM, Andorf JL, Whitmore SS, et al. Clinically Focused Molecular Investigation of 1000 Consecutive Families with Inherited Retinal Disease. *Ophthalmology*. 2017;124(9). doi:10.1016/j.ophtha.2017.04.008
92. Chung MM, Oh KT, Streb LM, Kimura AE, Stone EM. Visual outcome following subretinal hemorrhage in best disease. *Retina*. 2001;21(6). doi:10.1097/00006982-200112000-00003
93. Kay CN, Abramoff MD, Mullins RF, et al. Three-dimensional distribution of the vitelliform lesion, photoreceptors, and retinal pigment epithelium in the macula of patients with best vitelliform macular dystrophy. *Arch Ophthalmol*. 2012;130(3). doi:10.1001/archophthalmol.2011.363
94. Chibani Z, Abid IZ, Molbaek A, Söderkvist P, Feki J, Hmani-Aifa M. Novel BEST1 gene mutations associated with two different forms of macular dystrophy in Tunisian families. *Clin Exp Ophthalmol*. 2019;47(8). doi:10.1111/ceo.13577
95. Habibi I, Falfoul Y, Todorova MG, et al. Clinical and genetic findings of autosomal recessive bestrophinopathy (ARB). *Genes (Basel)*. 2019;10(12). doi:10.3390/genes10120953
96. Hufendiek K, Hufendiek K, Jägle H, et al. Clinical heterogeneity in autosomal recessive bestrophinopathy with biallelic mutations in the BEST1 gene. *Int J Mol Sci*. 2020;21(24). doi:10.3390/ijms21249353
97. Davidson AE, Sergouniotis PI, Burgess-Mullan R, et al. A synonymous codon variant in two patients with autosomal recessive bestrophinopathy alters in vitro splicing of BEST1. *Mol Vis*. 2010;16.
98. Pfister TA, Zein WM, Cukras CA, et al. Phenotypic and genetic spectrum of autosomal recessive bestrophinopathy and best vitelliform macular dystrophy. *Investig Ophthalmol Vis Sci*. 2021;62(6). doi:10.1167/IOVS.62.6.22
99. Jun I, Lee JS, Lee JH, et al. Adult-Onset Vitelliform Macular Dystrophy caused by BEST1 p.Ile38Ser Mutation is a Mild Form of Best Vitelliform Macular Dystrophy. *Sci Rep*. 2017;7(1). doi:10.1038/s41598-017-09629-9
100. Lee JH, Oh JO, Lee CS. Induced pluripotent stem cell modeling of best disease and autosomal recessive bestrophinopathy. *Yonsei Med J*. 2020;61(9). doi:10.3349/ymj.2020.61.9.816
101. Burgess R, Millar ID, Leroy BP, et al. Biallelic Mutation of BEST1 Causes a Distinct Retinopathy in Humans. *Am J Hum Genet*. 2008;82(1). doi:10.1016/j.ajhg.2007.08.004
102. Zhao L, Grob S, Corey R, et al. A novel compound heterozygous mutation in the BEST1 gene causes autosomal recessive Best vitelliform macular dystrophy. *Eye*. 2012;26(6). doi:10.1038/eye.2012.27
103. Kinnick TR, Mullins RF, Dev S, et al. Autosomal recessive vitelliform macular dystrophy in a large cohort of vitelliform macular dystrophy patients. *Retina*. 2011;31(3). doi:10.1097/IAE.0b013e318203ee60
104. Sodi A, Menchini F, Manitto MP, et al. Ocular phenotypes associated with biallelic mutations in BEST1 in Italian patients. *Mol Vis*. 2011;17.
105. Wang P, Li S, Sun W, et al. An ophthalmic targeted exome sequencing panel as a powerful tool to identify causative mutations in patients suspected of hereditary eye diseases. *Transl Vis Sci Technol*. 2019;8(2). doi:10.1167/tvst.8.2.21
106. Nguyen TT, Poornachandra B, Verma A, et al. Next generation sequencing identifies

novel diseaseassociated BEST1 mutations in Bestrophinopathy patients. *Sci Rep*. 2018;8(1). doi:10.1038/s41598-018-27951-8
107. Dev Borman A, Davidson AE, O'Sullivan J, et al. Childhood-onset autosomal recessive bestrophinopathy. *Arch Ophthalmol*. 2011;129(8). doi:10.1001/archophthalmol.2011.197
108. Huang X, Xiao X, Jia X, et al. Mutation analysis of the genes associated with anterior segment dysgenesis, microcornea and microphthalmia in 257 patients with glaucoma. *Int J Mol Med*. 2015;36(4). doi:10.3892/ijmm.2015.2325
109. Jaffal L, Joumaa WH, Assi A, et al. Novel missense mutations in BEST1 Are associated with bestrophinopathies in lebanese patients. *Genes (Basel)*. 2019;10(2). doi:10.3390/genes10020151
110. Domingo-Prim J, Riera M, Abad-Morales V, Ruiz-Nogales S, Corcostegui B, Pomares E. Generation of Best disease-derived induced pluripotent stem cell line (FRIMOi006-A) carrying a novel dominant mutation in BEST1 gene. *Stem Cell Res*. 2019;40. doi:10.1016/j.scr.2019.101570
111. Gattoussi S, Boon CJF, Freund KB. Maintenance of Good Visual Acuity in Best Disease Associated with Chronic Bilateral Serous Macular Detachment. *Retin Cases Br Reports*. 2020;14(1). doi:10.1097/ICB.0000000000000618
112. Zampaglione E, Kinde B, Place EM, et al. Copy-number variation contributes 9% of pathogenicity in the inherited retinal degenerations. *Genet Med*. 2020;22(6). doi:10.1038/s41436-020-0759-8
113. Chen CJ, Kaufman S, Packo K, Stöhr H, Weber BHF, Goldberg MF. Long-Term Macular Changes in the First Proband of Autosomal Dominant Vitreoretinochoroidopathy (ADVIRC) Due to a Newly Identified Mutation in BEST1. *Ophthalmic Genet*. 2016;37(1). doi:10.3109/13816810.2015.1039893
114. Goldberg MF, McLeod S, Tso M, et al. Ocular Histopathology and Immunohistochemical Analysis in the Oldest Known Individual with Autosomal Dominant Vitreoretinochoroidopathy. *Ophthalmol Retin*. 2018;2(4). doi:10.1016/j.oret.2017.08.001
115. Wittström E, Ekvall S, Schatz P, Bondeson ML, Ponjavic V, Andréasson S. Morphological and functional changes in multifocal vitelliform retinopathy and biallelic mutations in BEST1. *Ophthalmic Genet*. 2011;32(2). doi:10.3109/13816810.2010.535890
116. Yardley J, Leroy BP, Hart-Holden N, et al. Mutations of VMD2 splicing regulators cause nanophthalmos and autosomal dominant vitreoretinochoroidopathy (ADVIRC). *Investig Ophthalmol Vis Sci*. 2004;45(10). doi:10.1167/iovs.04-0550
117. Vincent A, McAlister C, Vandenhoven C, Héon E. BEST1-related autosomal dominant vitreoretinochoroidopathy: A degenerative disease with a range of developmental ocular anomalies. *Eye*. 2011;25(1). doi:10.1038/eye.2010.165
118. Kellner S, Stöhr H, Fiebig B, et al. Fundus Autofluorescence and SD-OCT Document Rapid Progression in Autosomal Dominant Vitreoretinochoroidopathy (ADVIRC) Associated with a c.256G > A Mutation in BEST1. *Ophthalmic Genet*. 2016;37(2). doi:10.3109/13816810.2015.1033556
119. Boulanger-Scemama E, Sahel JA, Mohandsaid S, et al. Autosomal dominant vitreoretinochoroidopathy when molecular genetic testing helps clinical diagnosis. *Retina*. 2019;39(5). doi:10.1097/IAE.0000000000002041
120. Gerth C, Zawadzki RJ, Werner JS, Héon E. Detailed analysis of retinal function and morphology in a patient with autosomal recessive bestrophinopathy (ARB). *Doc Ophthalmol*. 2009;118(3). doi:10.1007/s10633-008-9154-5
121. Deák GG, Schmidt WM, Bittner RE, et al. Imaging of vitelliform macular lesions using polarization-sensitive optical coherence tomography. *Retina*. 2019;39(3). doi:10.1097/IAE.0000000000001987
122. Bakall B, Radu RA, Stanton JB, et al. Enhanced accumulation of A2E in individuals homozygous or heterozygous for mutations in BEST1 (VMD2). *Exp Eye Res*. 2007;85(1). doi:10.1016/j.exer.2007.02.018
123. Zolnikova I V., Strelnikov V V., Skvortsova NA, et al. Stargardt disease-associated mutation spectrum of a Russian Federation cohort. *Eur J Med Genet*. 2017;60(2). doi:10.1016/j.ejmg.2016.12.002
124. Frennesson CI, Wadelius C, Nilsson SEG. Best vitelliform macular dystrophy in a Swedish family: Genetic analysis and a seven-year follow-up of photodynamic

treatment of a young boy with choroidal neovascularization. *Acta Ophthalmol*. 2014;92(3). doi:10.1111/aos.12142
125. Pianta MJ, Aleman TS, Cideciyan A V., et al. In vivo micropathology of best macular dystrophy with optical coherence tomography. *Exp Eye Res*. 2003;76(2). doi:10.1016/S0014-4835(02)00280-4
126. Al-Abri M, Al-Hinai A, Al Zuhaibi S, Ganesh A, Al Ghafri A, Al-Thihli K. Normal electro-oculography in a young Omani male with genetically confirmed best disease complicated by choroidal neovascularization. *Oman J Ophthalmol*. 2019;12(1). doi:10.4103/ojo.OJO_74_2018
127. Liu X, Tao T, Zhao L, Li G, Yang L. Molecular diagnosis based on comprehensive genetic testing in 800 Chinese families with non-syndromic inherited retinal dystrophies. *Clin Exp Ophthalmol*. 2021;49(1). doi:10.1111/ceo.13875
128. Lin Y, Li T, Gao H, et al. Bestrophin 1 gene analysis and associated clinical findings in a Chinese patient with best vitelliform macular dystrophy. *Mol Med Rep*. 2017;16(4). doi:10.3892/mmr.2017.7174
129. Wabbels B, Preising MN, Kretschmann U, Demmler A, Lorenz B. Genotype-phenotype correlation and longitudinal course in ten families with Best vitelliform macular dystrophy. *Graefe's Arch Clin Exp Ophthalmol*. 2006;244(11). doi:10.1007/s00417-006-0286-6
130. Ellingford JM, Barton S, Bhaskar S, et al. Molecular findings from 537 individuals with inherited retinal disease. *J Med Genet*. Published online 2016. doi:10.1136/jmedgenet-2016-103837
131. Avela K, Sankila EM, Seitsonen S, et al. A founder mutation in CERKL is a major cause of retinal dystrophy in Finland. *Acta Ophthalmol*. 2018;96(2). doi:10.1111/aos.13551
132. Lin Y, Li T, Liu B, et al. Multimodal imaging and genetic analysis of adult-onset best vitelliform macular dystrophy in Chinese patients. *Exp Ther Med*. 2021;22(3). doi:10.3892/etm.2021.10466
133. Ramkumar HL, Gudiseva H V., Kishaba KT, et al. A report on molecular diagnostic testing for inherited retinal dystrophies by targeted genetic analyses. *Genet Test Mol Biomarkers*. 2017;21(2). doi:10.1089/gtmb.2016.0251
134. Li Y, Wang G, Dong B, et al. A novel mutation of the VMD2 gene in a Chinese family with best vitelliform macular dystrophy. *Ann Acad Med Singapore*. 2006;35(6). doi:10.47102/annals-acadmedsg.v35n6p408
135. Allikmets R, Seddon JM, Bernstein PS, et al. Evaluation of the Best disease gene in patients with age-related macular degeneration and other maculopathies. *Hum Genet*. 1999;104(6). doi:10.1007/s004390050986
136. Seddon JM, Afshari MA, Sharma S, et al. Assessment of mutations in the Best macular dystrophy (VMD2) gene in patients with adult-onset foveomacular vitelliform dystrophy, age-related maculopathy, and bull's-eye maculopathy. *Ophthalmology*. 2001;108(11). doi:10.1016/S0161-6420(01)00777-1
137. Huang L, Zhang Q, Huang X, et al. Mutation screening in genes known to be responsible for Retinitis Pigmentosa in 98 Small Han Chinese Families. *Sci Rep*. 2017;7(1). doi:10.1038/s41598-017-00963-6
138. Ng TK, Cao Y, Yuan XL, et al. Whole exome sequencing analysis identifies novel Stargardt disease-related gene mutations in Chinese Stargardt disease and retinitis pigmentosa patients. *Eye*. 2022;36(4). doi:10.1038/s41433-021-01525-x
139. Piñeiro-Gallego T, Álvarez M, Pereiro I, et al. Clinical evaluation of two consanguineous families with homozygous mutations in BEST1. *Mol Vis*. 2011;17.
140. Marchant D, Yu K, Bigot K, et al. New VMD2 gene mutations identified in patients affected by Best vitelliform macular dystrophy. *J Med Genet*. 2007;44(3). doi:10.1136/jmg.2006.044511
141. Martin-Merida I, Aguilera-Garcia D, Fernandez-San Jose P, et al. Toward the mutational landscape of autosomal dominant retinitis pigmentosa: A comprehensive analysis of 258 Spanish families. *Investig Ophthalmol Vis Sci*. 2018;59(6). doi:10.1167/iovs.18-23854
142. Hussain RN, Shahid FL, Empeslidis T, Chng SW. Use of Intravitreal Bevacizumab in a 9-Year-Old Child with Choroidal Neovascularization Associated with Autosomal Recessive Bestrophinopathy. *Ophthalmic Genet*. 2015;36(3). doi:10.3109/13816810.2014.962706

143. Gottwalt S, Bergmann A, Kautza-Lucht M, Roider JB, Treumer F. Extensive yellowish fundus changes in a 6-year-old child. *Ophthalmologe*. 2017;114(6). doi:10.1007/s00347-016-0336-3
144. Davidson AE, Millar ID, Urquhart JE, et al. Missense Mutations in a Retinal Pigment Epithelium Protein, Bestrophin-1, Cause Retinitis Pigmentosa. *Am J Hum Genet*. 2009;85(5). doi:10.1016/j.ajhg.2009.09.015
145. Iannaccone A, Kerr NC, Kinnick TR, Calzada JI, Stone EM. Autosomal recessive best vitelliform macular dystrophy: Report of a family and management of early-onset neovascular complications. *Arch Ophthalmol*. 2011;129(2). doi:10.1001/archophthalmol.2010.367
146. Carrigan M, Duignan E, Malone CPG, et al. Panel-Based population next-generation sequencing for inherited retinal degenerations. *Sci Rep*. 2016;6. doi:10.1038/srep33248
147. Macdonald IM, Gudiseva H V., Villanueva A, Greve M, Caruso R, Ayyagari R. Phenotype and genotype of patients with autosomal recessive bestrophinopathy. *Ophthalmic Genet*. 2012;33(3). doi:10.3109/13816810.2011.592172
148. Johnson AA, Bachman LA, Gilles BJ, et al. Autosomal recessive bestrophinopathy is not associated with the loss of bestrophin-1 anion channel function in a patient with a novel BEST1 mutation. *Investig Ophthalmol Vis Sci*. 2015;56(8). doi:10.1167/iovs.15-16910
149. Jansson RW, Berland S, Bredrup C, Austeng D, Andréasson S, Wittström E. Biallelic Mutations in the BEST1 Gene: Additional Families with Autosomal Recessive Bestrophinopathy. *Ophthalmic Genet*. 2016;37(2). doi:10.3109/13816810.2015.1020558
150. Todorova MG, Bojinova RI, Valmaggia C, Schorderet DF. Prominent Optic Disc Featured in Inherited Retinopathy. *Klin Monbl Augenheilkd*. 2017;234(4). doi:10.1055/s-0042-121335
151. Tiwari A, Lemke J, Altmueller J, et al. Identification of novel and recurrent disease-causing mutations in retinal dystrophies using whole exome sequencing (WES): Benefits and limitations. *PLoS One*. Published online 2016. doi:10.1371/journal.pone.0158692
152. Wang L, Zhang J, Chen N, et al. Application of whole exome and targeted panel sequencing in the clinical molecular diagnosis of 319 Chinese families with inherited retinal dystrophy and comparison study. *Genes (Basel)*. 2018;9(7). doi:10.3390/genes9070360
153. Yamada R, Takagi R, Iwamoto S, Shimada S, Kakehashi A. Novel BEST1 mutation in autosomal recessive bestrophinopathy in Japanese siblings. *Taiwan J Ophthalmol*. 2021;11(1). doi:10.4103/tjo.tjo_37_20
154. Wang S, Zhang Q, Zhang X, Wang Z, Zhao P. Clinical and genetic characteristics of Leber congenital amaurosis with novel mutations in known genes based on a Chinese eastern coast Han population. *Graefe's Arch Clin Exp Ophthalmol*. Published online 2016. doi:10.1007/s00417-016-3428-5
155. Pomares E, Burés-Jelstrup A, Ruiz-Nogales S, Corcóstegui B, González-Duarte R, Navarro R. Nonsense-mediated decay as the molecular cause for autosomal recessive bestrophinopathy in two unrelated families. *Investig Ophthalmol Vis Sci*. 2012;53(1). doi:10.1167/iovs.11-7964
156. Soto-Sierra M, Morillo-Sánchez MJ, Martín-Sánchez M, et al. Novel BEST1 mutations and clinical characteristics of autosomal recessive bestrophinopathy in a Spanish patient. *Eur J Ophthalmol*. 2022;32(5). doi:10.1177/11206721211010615
157. Cascavilla ML, Querques G, Stenirri S, Battaglia Parodi M, Querques L, Bandello F. Unilateral vitelliform phenotype in autosomal recessive bestrophinopathy. *Ophthalmic Res*. 2012;48(3). doi:10.1159/000338750
158. Sharon D, Al-Hamdani S, Engelsberg K, et al. Ocular phenotype analysis of a family with biallelic mutations in the BEST1 gene. *Am J Ophthalmol*. 2014;157(3). doi:10.1016/j.ajo.2013.12.010
159. Weisschuh N, Mayer AK, Strom TM, et al. Mutation detection in patients with retinal dystrophies using targeted next generation sequencing. *PLoS One*. 2016;11(1). doi:10.1371/journal.pone.0145951
160. Haer-Wigman L, Van Zelst-Stams WAG, Pfundt R, et al. Diagnostic exome sequencing in 266 Dutch patients with visual impairment. *Eur J Hum Genet*.

2017;25(5). doi:10.1038/ejhg.2017.9
161. Ma DJ, Lee HS, Kim K, et al. Whole-exome sequencing in 168 Korean patients with inherited retinal degeneration. *BMC Med Genomics*. 2021;14(1). doi:10.1186/s12920-021-00874-6
162. Introini U, Casalino G, Khan KN, et al. Clinical course of autosomal recessive bestrophinopathy complicated by choroidal neovascularization. *Ophthalmic Surg Lasers Imaging Retin*. 2018;49(11). doi:10.3928/23258160-20181101-10
163. Guerriero S, Preising MN, Ciccolella N, Causio F, Lorenz B, Fischetto R. Autosomal recessive bestrophinopathy: New observations on the retinal phenotype - Clinical and molecular report of an Italian family. *Ophthalmologica*. 2011;225(4). doi:10.1159/000324472
164. Bonilha VL, Bell BA, DeBenedictis MJ, Hagstrom SA, Fishman GA, Hollyfield JG. Cellular Changes in Retinas From Patients With BEST1 Mutations. *Front Cell Dev Biol*. 2020;8. doi:10.3389/fcell.2020.573330
165. Hitti-Malin RJ, Dhaenens CM, Panneman DM, et al. Using single molecule Molecular Inversion Probes as a cost-effective, high-throughput sequencing approach to target all genes and loci associated with macular diseases. *Hum Mutat*. 2022;43(12). doi:10.1002/humu.24489
166. Toto L, Boon CJF, Di Antonio L, et al. Bestrophinopathy: A spectrum of ocular abnormalities caused by the c.614T.C mutation in the BEST1 gene. *Retina*. 2016;36(8). doi:10.1097/IAE.0000000000000950
167. Kopsidas K, Javidi H, Kelly SP, Aslam T, Black G, Mahmood S. Unique Case of Bilateral Exudative Retinal Detachment following Creatine Supplementation in a Patient with Autosomal Dominant Bestrophinopathy. *Case Rep Ophthalmol*. 2019;10(3). doi:10.1159/000503853
168. Wang J, Zhang VW, Feng Y, et al. Dependable and efficient clinical utility of target capture-based deep sequencing in molecular diagnosis of retinitis pigmentosa. *Investig Ophthalmol Vis Sci*. Published online 2014. doi:10.1167/iovs.14-14936
169. Crowley C, Paterson R, Lamey T, et al. Autosomal recessive bestrophinopathy associated with angle-closure glaucoma. *Doc Ophthalmol*. 2014;129(1). doi:10.1007/s10633-014-9444-z
170. Fung A, Yzer S, Allikmets R. Clinical and genetic misdiagnosis of autosomal recessive bestrophinopathy. *JAMA Ophthalmol*. 2013;131(12). doi:10.1001/jamaophthalmol.2013.5363
171. Tracewska AM, Kocyła-Karczmarewicz B, Rafalska A, et al. Non-syndromic inherited retinal diseases in Poland: Genes, mutations, and phenotypes. *Mol Vis*. 2021;27.
172. Atchaneeyasakul LO, Jinda W, Sakolsatayadorn N, et al. Mutation analysis of the VMD2 gene in thai families with best macular dystrophy. *Ophthalmic Genet*. 2008;29(3). doi:10.1080/13816810802087394
173. Zhu J, Stephenson KAJ, Dockery A, et al. Electrophysiology-Guided Genetic Characterisation Maximises Molecular Diagnosis in an Irish Paediatric Inherited Retinal Degeneration Population. *Genes (Basel)*. 2022;13(4). doi:10.3390/genes13040615
174. Kousal B, Chakarova F, Black GC, Ramsden S, Langrova H, Liskova P. [Minimal ocular findings in a patient with Best disease caused by the c.653G>A mutation in BEST1]. [Czech]. *Cesk Slov Oftalmol*. 2011;67(5-6).
175. Hardin JS, Schaefer GB, Sallam AB, Williams MK, Uwaydat S. A unique case series of autosomal recessive bestrophinopathy exhibiting multigenerational inheritance. *Ophthalmic Genet*. 2017;38(6). doi:10.1080/13816810.2017.1318926
176. Moshfegh Y, Velez G, Li Y, Bassuk AG, Mahajan VB, Tsang SH. BESTROPHIN1 mutations cause defective chloride conductance in patient stem cell-derived RPE. *Hum Mol Genet*. 2016;25(13). doi:10.1093/hmg/ddw126
177. Palomba G, Rozzo C, Angius A, Pierrottet CO, Orzalesi N, Pirastu M. A novel spontaneous missense mutation in VMD2 gene is a cause of a best macular dystrophy sporadic case. *Am J Ophthalmol*. 2000;129(2). doi:10.1016/S0002-9394(99)00327-X
178. Mullins RF, Oh KT, Heffron E, Hageman GS, Stone EM. Late development of vitelliform lesions and flecks in a patient with best disease: Clinicopathologic correlation. *Arch Ophthalmol*. 2005;123(11). doi:10.1001/archopht.123.11.1588
179. Schönbach EM, Scholl HP. Fundus autofluorescence in a subclinical case of best disease. *Retin Cases Br Reports*. 2017;11. doi:10.1097/ICB.0000000000000390

180. van Huet RAC, Pierrache LHM, Meester-Smoor MA, et al. The efficacy of microarray screening for autosomal recessive retinitis pigmentosa in routine clinical practice. *Mol Vis*. 2015;21.
181. Hosono K, Nishina S, Yokoi T, et al. Molecular diagnosis of 34 Japanese families with leber congenital amaurosis using targeted next generation sequencing. *Sci Rep*. Published online 2018. doi:10.1038/s41598-018-26524-z
182. Lin Y, Gao H, Liu Y, et al. Two novel mutations in the bestrophin-1 gene and associated clinical observations in patients with best vitelliform macular dystrophy. *Mol Med Rep*. 2015;12(2). doi:10.3892/mmr.2015.3711
183. Burgess R, MacLaren RE, Davidson AE, et al. ADVIRC is caused by distinct mutations in BEST1 that alter pre-mRNA splicing. *J Med Genet*. 2009;46(9). doi:10.1136/jmg.2008.059881
184. Kubota D, Gocho K, Akeo K, et al. Detailed analysis of family with autosomal recessive bestrophinopathy associated with new BEST1 mutation. *Doc Ophthalmol*. 2016;132(3). doi:10.1007/s10633-016-9540-3
185. Pollack K, Kreuz FR, Pillunat LE. Best's disease with normal EOG. Case report of familial macular dystrophy. *Ophthalmologe*. 2005;102(9). doi:10.1007/s00347-004-1088-z
186. Sheng X, Chen X, Zhao K, Liu Y, Vollrath D, Zhao C. A novel homozygous BEST1 mutation correlates with complex ocular phenotypes. *Ophthalmology*. 2013;120(7). doi:10.1016/j.ophtha.2013.03.043
187. Oishi M, Oishi A, Gotoh N, et al. Comprehensive molecular diagnosis of a large cohort of Japanese retinitis pigmentosa and usher syndrome patients by next-generation sequencing. *Investig Ophthalmol Vis Sci*. 2014;55(11). doi:10.1167/iovs.14-15458
188. Preising MN, Pasquay C, Friedburg C, et al. Autosomal Recessive Bestrophinopathy (ARB): A Clinical and Molecular Description of Two Patients at Childhood. *Klin Monbl Augenheilkd*. 2012;229(10). doi:DOI 10.1055/s-0032-1327782
189. Zaneveld J, Siddiqui S, Li H, et al. Comprehensive analysis of patients with Stargardt macular dystrophy reveals new genotype-phenotype correlations and unexpected diagnostic revisions. *Genet Med*. 2015;17(4). doi:10.1038/gim.2014.174
190. Patel N, Aldahmesh MA, Alkuraya H, et al. Expanding the clinical, allelic, and locus heterogeneity of retinal dystrophies. *Genet Med*. 2016;18(6). doi:10.1038/gim.2015.127
191. Zenteno JC, García-Montaño LA, Cruz-Aguilar M, et al. Extensive genic and allelic heterogeneity underlying inherited retinal dystrophies in Mexican patients molecularly analyzed by next-generation sequencing. *Mol Genet Genomic Med*. 2020;8(1). doi:10.1002/mgg3.1044
192. Garza-Garza LA, León-Cachón RBR, Aguirre-Garza M, Garza-Leon M. "Novel p.Tyr284Cys BEST1 genotype–phenotype correlations of Vitelliform Macular Dystrophy in a family with incomplete penetrance." *Ophthalmic Genet*. 2020;41(2). doi:10.1080/13816810.2020.1744020
193. Sun W, Huang L, Xu Y, et al. Exome sequencing on 298 probands with early-onset high myopia: Approximately one-fourth show potential pathogenic mutations in RetNet genes. *Investig Ophthalmol Vis Sci*. 2015;56(13). doi:10.1167/iovs.15-17555
194. Yanagi Y, Sekine H, Mori M. Identification of a novel VMD2 mutation in Japanese patients with Best disease. *Ophthalmic Genet*. 2002;23(2). doi:10.1076/opge.23.2.129.2213
195. Kim MS, Joo K, Seong MW, et al. Genetic mutation profiles in Korean patients with inherited retinal diseases. *J Korean Med Sci*. 2019;34(21). doi:10.3346/jkms.2019.34.e161
196. Seddon JM, Sharma S, Chong S, Hutchinson A, Allikmets R, Adelman RA. Phenotype and genotype correlations in two best families. *Ophthalmology*. 2003;110(9). doi:10.1016/S0161-6420(03)00575-X
197. Ouyang YL, Zhang YJ, Xu GZ, Jiang R, Chen Q, Wang L. Clinical manifestations and gene analysis in one Chinese family with Best vitelliform macular dystrophy. *Chinese J Ophthalmol*. 2008;44(4).
198. Lin Y, Li T, Ma C, et al. Genetic variations in Bestrophin-1 and associated clinical findings in two Chinese patients with juvenile-onset and adult-onset best vitelliform macular dystrophy. *Mol Med Rep*. 2018;17(1). doi:10.3892/mmr.2017.7927
199. Li Y, Bracha P, Aleman TS, Brucker AJ. ADULT-ONSET BEST1 -VITELLIFORM

DYSTROPHY ASSOCIATED WITH ANGIOID STREAK-LIKE CHANGES IN TWO SIBLINGS. *Retin Cases Br Reports*. 2023;17(3). doi:10.1097/ICB.0000000000001164
200. Marchant D, Gogat K, Dureau P, et al. Use of denaturing HPLC and automated sequencing to screen the VMD2 gene for mutations associated with Best's vitelliform macular dystrophy. *Ophthalmic Genet*. 2002;23(3). doi:10.1076/opge.23.3.167.7880
201. Peiretti E, Caminiti G, Forma G, et al. Novel P.Asp304gly mutation in best1 gene associated with atypical best vitelliform macular dystrophy phenotype and high intrafamilial variability. *Retina*. 2016;36(9). doi:10.1097/IAE.0000000000000966
202. Testa F, Rossi S, Passerini I, et al. A normal electro-oculography in a family affected by best disease with a novel spontaneous mutation of the BEST1 gene. *Br J Ophthalmol*. 2008;92(11). doi:10.1136/bjo.2008.143776
203. Low S, Davidson AE, Holder GE, et al. Autosomal dominant Best disease with an unusual electrooculographic light rise and risk of angle-closure glaucoma: A clinical and molecular genetic study. *Mol Vis*. 2011;17.
204. Ye P, Xu J, Luo Y, Su Z, Yao K. Familial autosomal recessive bestrophinopathy: Identification of a novel variant in BEST1 gene and the specific metabolomic profile. *BMC Med Genet*. 2020;21(1). doi:10.1186/s12881-020-0951-3
205. Riera M, Navarro R, Ruiz-Nogales S, et al. Whole exome sequencing using Ion Proton system enables reliable genetic diagnosis of inherited retinal dystrophies. *Sci Rep*. Published online 2017. doi:10.1038/srep42078
206. Li T, Lin Y, Gao H, et al. Two heterozygous mutations identified in one Chinese patient with bilateral macular coloboma. *Mol Med Rep*. 2017;16(3). doi:10.3892/mmr.2017.6887
207. Kersten E, Geerlings MJ, Pauper M, et al. Genetic screening for macular dystrophies in patients clinically diagnosed with dry age-related macular degeneration. *Clin Genet*. 2018;94(6). doi:10.1111/cge.13447
208. Silva RA, Berrocal AM, Lam BL, Albini TA. Novel mutation in BEST1 associated with retinoschisis. *JAMA Ophthalmol*. 2013;131(6). doi:10.1001/jamaophthalmol.2013.2047
209. Dalvin LA, Pulido JS, Marmorstein AD. Vitelliform dystrophies: Prevalence in Olmsted County, Minnesota, United States. *Ophthalmic Genet*. 2017;38(2). doi:10.1080/13816810.2016.1175645
210. Beit-Ya'acov A, Mizrahi-Meissonnier L, Obolensky A, et al. Homozygosity for a novel ABCA4 founder splicing mutation is associated with progressive and severe Stargardt-like disease. *Investig Ophthalmol Vis Sci*. Published online 2007. doi:10.1167/iovs.07-0244
211. Brandl C, Schulz HL, Charbel Issa P, et al. Mutations in the Genes for Interphotoreceptor Matrix Proteoglycans, IMPG1 and IMPG2, in Patients with Vitelliform Macular Lesions. *Genes (Basel)*. 2017;8(7). doi:10.3390/GENES8070170
212. Felbor U, Schilling H, Weber BHF. Adult vitelliform macular dystrophy is frequently associated with mutations in the peripherin/RDS gene. *Hum Mutat*. 1997;10(4). doi:10.1002/(SICI)1098-1004(1997)10:4<301::AID-HUMU6>3.0.CO;2-J
